# Amazonian amphibians: diversity, spatial distribution patterns, conservation and sampling deficits

**DOI:** 10.3897/BDJ.12.e109785

**Published:** 2024-10-01

**Authors:** Marcos Penhacek, Thadeu Sobral Souza, Jessie Pereira Santos, Vinicius Guerra, Rodrigo Antônio Castro-Souza, Domingos de Jesus Rodrigues

**Affiliations:** 1 Programa de Pós-Graduação em Ecologia e Conservação da Biodiversidade, Universidade Federal do Mato Grosso, Rua Fernando Corrêa da Costa 2367, 78060-900, Cuiabá, Brazil Programa de Pós-Graduação em Ecologia e Conservação da Biodiversidade, Universidade Federal do Mato Grosso, Rua Fernando Corrêa da Costa 2367, 78060-900 Cuiabá Brazil; 2 Centro de Biodiversidade, Instituto de Biociências, Departamento de Botânica e Ecologia, Universidade Federal do Mato Grosso, Rua Fernando Corrêa da Costa 2367, 78060-900, Cuiabá, Brazil Centro de Biodiversidade, Instituto de Biociências, Departamento de Botânica e Ecologia, Universidade Federal do Mato Grosso, Rua Fernando Corrêa da Costa 2367, 78060-900 Cuiabá Brazil; 3 Programa de Pós-Graduação em Ecologia e Conservação da Biodiversidade, Universidade Federal do Mato Grosso, Rua Fernando Corrêa da Costa 2367, Cuiabá, MT, Brazil Programa de Pós-Graduação em Ecologia e Conservação da Biodiversidade, Universidade Federal do Mato Grosso, Rua Fernando Corrêa da Costa 2367 Cuiabá, MT Brazil; 4 Universidade Estadual de Campinas (UNICAMP), Departamento de Biologia Animal, Instituto de Biologia, Campinas, SP, Brazil Universidade Estadual de Campinas (UNICAMP), Departamento de Biologia Animal, Instituto de Biologia Campinas, SP Brazil; 5 Instituto de Ciências Naturais, Humanas e Sociais, Acervo Biológico da Amazônia Meridional, Universidade Federal de Mato Grosso, Sinop, Brazil Instituto de Ciências Naturais, Humanas e Sociais, Acervo Biológico da Amazônia Meridional, Universidade Federal de Mato Grosso Sinop Brazil; 6 Instituto Boitatá de Etnobiologia e Conservação da Fauna, Goiânia, GO, Brazil Instituto Boitatá de Etnobiologia e Conservação da Fauna Goiânia, GO Brazil; 7 Instituto de Ciências Naturais, Humanas e Sociais, Acervo Biológico da Amazônia Meridional, Universidade Federal de Mato Grosso, Sinop, MT, Brazil Instituto de Ciências Naturais, Humanas e Sociais, Acervo Biológico da Amazônia Meridional, Universidade Federal de Mato Grosso Sinop, MT Brazil

**Keywords:** Amphibians, Amazon Basin, biodiversity, sample completeness, sample gaps, Neotropical Region

## Abstract

The Amazon biome is home to the largest tropical forest on the planet and has the greatest global biodiversity on Earth. Despite this, several less charismatic taxonomic groups, such as amphibians, lack comprehensive studies on their species richness and spatial distribution in the Amazon Region. In this study, we investigated: i) patterns of richness and endemism of Amazonian amphibians across geopolitical and biogeographic divisions, ii) similarities between different Amazonian bioregions, iii) temporal trends in amphibian sampling, iv) conservation status of amphibians according to assessments of the IUCN and v) the importance of diverse data sources in building a robust database of amphibian occurrences. We aggregated data from four different sources: publicly accessible platforms, peer-reviewed articles, grey literature and fieldwork inventories spanning 15 years (2007–2021), ultimately compiling 160,643 records of 947 species across 7,418 sampled sites. The greatest diversity of species was found in Peru, Brazil and Ecuador, with notable amphibian diversity and endemism in regions such as the western basins and the Tapajós River Basin in the central-southern Amazon. Geographical analysis of species diversity revealed four distinct groups defined by latitudinal (the Amazon River) and longitudinal (the Juruá, Madeira and Tapajós Rivers) gradients, with low species similarity (< 40%), particularly in the basins of north-western Amazonia. Amphibian sampling in the Amazon has intensified since the 1950s with the establishment of important research centres such as INPA and the GOELD Museum in the Brazilian Amazon. Approximately 18% of Amazonian amphibian species face extinction risk, according to IUCN assessments, highlighting the need for comprehensive data sources to understand and conserve species in this megadiverse region. Our findings suggest that river systems likely influence Amazonian amphibian species composition due to biogeographic history, emphasising the need for robust taxonomic and spatial databases. This study, therefore, contributes a valuable large-scale dataset for Amazonian amphibians, guiding future research and strategies for amphibian conservation.

## Introduction

The Amazon is a megadiverse region critical for maintaining global hydrological and climatic cycles (Fearnside 1999, Stuart et al. 2004, Sobral-Souza et al. 2015, Nobre et al. 2021, Albert et al. 2023). It currently holds nearly 50% of the world's remaining tropical forest (Santos et al. 2009), encompassing the largest freshwater reservoir (Amazon Basin) and harbouring over 10% of the planet's known biodiversity (Dirzo and Raven 2003, Tisseuil et al. 2013, Tedesco et al. 2017, Oberdorff et al. 2019, Guayasamin et al. 2021). The Amazonian biota has been diversifying and structuring for over 100 million years through dynamic combinations of geological, climatic and evolutionary processes (Musher et al. 2019, Guayasamin et al. 2021, Almeida-Silva et al. 2024). Over time, geological and climatic events have altered geographical connections and consequently gene flow amongst populations, impacting adaptation rates, speciation and species dispersal throughout the Neotropical Region (Dexter et al. 2017, Antonelli et al. 2018, Musher et al. 2019). Amongst the significant geological events that shaped the Amazonian biota, the uplift of the northern Andes (occurring over the last 20 million years) altered the entire Amazonian River system, giving rise to its current state (Latrubesse et al. 2017, Oberdorff et al. 2019, da Silva-Caminha et al. 2020, Espinosa et al. 2021, Leandro et al. 2022). This event separated the western tropical forests from the lowlands of the Amazon and created new environmental conditions for speciation (Cradle), colonisation (Museum) and species dispersal/extinction (Grave) of Amazonian megadiversity (Dexter et al. 2017, Musher et al. 2019).

However, just over 20,000 years ago, human populations began to colonise and modify the Amazon, accelerating after the Industrial Revolution (18^th^ century) and more intensely from the 1970s with colonisation incentives, now representing existential threats to all Amazonian ecosystems (Guayasamin et al. 2021). Industrialisation, urbanisation, dam construction, mining and especially agricultural frontier expansion have accelerated forest fragmentation, increasing the risk of Amazonian biodiversity loss and the loss of fundamental ecosystem services for global balance (Eterovick et al. 2005, Silvano and Segalla 2005, Keller et al. 2011, WWF-BRASI 2013, Ramos Neto 2019, Yanai et al. 2020). Currently, the region is increasingly exposed to unprecedented pressures resulting from rising temperatures, extreme droughts, deforestation and fires, causing a loss of forest resilience and increasing the risk of critical transitions towards development of savannahs (Nobre et al. 2021, Boulton et al. 2022, Flores et al. 2024). Therefore, to conserve the Amazon, it is imperative to prioritise the documentation and monitoring of its rich biodiversity, including the biogeographic distributions of its species, their abundance, phylogenetic diversity, physiological trait characteristics, interactions and ecosystem functions (Guayasamin et al. 2021).

During human expansion and colonisation of the Amazon, many records of biodiversity have been accumulated in different databases, natural history museums, scientific collections, herbaria and networks of citizen scientists (Anderson et al. 2020). Several initiatives have recently emerged to aggregate biodiversity data into digitally accessible platforms, such as the Global Biodiversity Information Facility GBIF (www.gbif.org), eBird (www.ebird.org) or iNaturalist (www.inaturalist.org), where scientists and the general public worldwide share field observations (Gábor et al. 2023). These digital biodiversity data have been widely used in ecology, evolution and conservation biology to develop species distribution models, predict invasive species invasions, prioritise conservation areas and forecast potential impacts of climate change on biodiversity and ecosystem services (Peterson et al. 2011, Hortal et al. 2015, Hallgren et al. 2016). However, despite its megadiversity and extensive network of navigable rivers, much of the Amazon remains inaccessible to researchers due to economic (high access costs) and legal (restrictive legislation, especially in Indigenous Territories) factors (Tuomisto 1998, Dos Santos et al. 2015, Carvalho et al. 2023). As a result, biological surveys in the Amazon have been heavily biased, spatially focused on easily accessible areas and/or those near major urban centres, leaving vast sampling gaps throughout the Amazon, hindering understanding of species distribution patterns (Giannini et al. 2012, Dos Santos et al. 2015, Oliveira et al. 2016, Guerra et al. 2020, Freitas et al. 2021, Andrade‐Silva et al. 2022).

Amphibians are the third richest group of terrestrial vertebrates, with 8,738 described species (Frost 2024). The structuring of amphibian communities is a complex process that involves positive interactions between biotic and abiotic factors, including water resources and temperature (Cortwright and Nelson 1990, Godinho and Da Silva 2018). Their semi-permeable skin and poikilothermic physiology make them highly dependent on moisture and stable temperatures, explaining their great diversity in the Neotropical Region, especially in humid forests, such as the Amazon (Wells 2007). Despite a wide range of reproductive modes, most amphibian species depend on lentic or lotic water bodies for reproduction. Consequently, their distribution tends to be restricted to environments near waterbodies, which serve as breeding grounds (Haddad and Prado 2005) and/or limit their distribution due to geographic barriers (dias‐Terceiro et al. 2015, Moraes et al. 2016, Godinho and Da Silva 2018). The dependence on aquatic and terrestrial environments in different life stages exposes amphibians to a wide range of environmental changes, making them excellent bioindicators (Becker et al. 2010, Amaral et al. 2019). Additionally, behavioural characteristics, such as limited mobility and the formation of reproductive congregations, can contribute to disease spread, such as chytridiomycosis caused by the fungus *Batrachochytriumdendrobatidis* and susceptibility to predation (Toledo 2009, Luedtke et al. 2023), placing them on the International Union for Conservation of Nature's Red List with the highest extinction risk rate amongst vertebrates, at 40.7% (Stuart et al. 2004, IUCN 2023, Luedtke et al. 2023).

Therefore, recognising the importance of adequate knowledge about biodiversity distribution patterns to support conservation efforts, our aim here was to compile a robust database with records of amphibian occurrences in the Amazon, aiming to: (i) document the richness and endemism of amphibian species in the Amazon, showing their spatial distribution patterns considering geopolitical divisions (countries) and biogeographic regions (drainage sub-basins); (ii) evaluate the temporal increase in the number of occurrences produced; (iii) identify the number of species in each extinction threat category (assessed by the IUCN (IUCN 2023); and (iv) demonstrate the importance of diverse data sources for understanding amphibian distribution patterns in the Amazon.

## Material and Methods

### Study area

**Figure 1.**
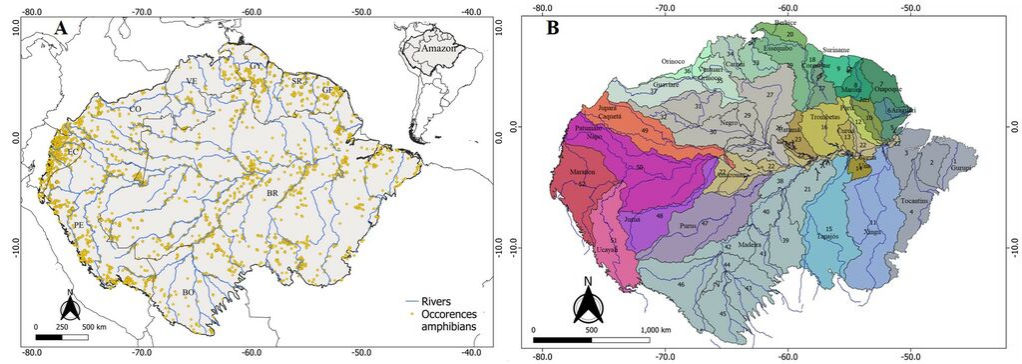
Biogeographic delimitation of the Amazon Ecoregion. **A** - Amazon basin studied, delimited by (WWF 2019). The yellow dots are occurrences of amphibians; **B** - Location of the 52 hydrographic sub-basins and the 30 rivers that drain the most water in the Amazon region. The name of each sub-basin is based on its main tributary river (see Suppl. material 1) .

In this study, we used the Amazon Ecoregion division proposed by the World Wildlife Fund - WWF (WWF 2019). The Amazon encompasses approximately 6.5 million km² extending across nine countries — Bolivia, Brazil, Colombia, Ecuador, Guyana, French Guiana, Peru, Suriname and Venezuela (WWF 2019) — with about 60% within Brazil (IBGE 2021). Located between latitudes 8º N and 17º S, it is bounded to the west by the Andes Mountains, to the north by the Guiana Plateau, to the south by the Central Plateau and to the east by the Atlantic Ocean (Sobral-Souza 2016, WWF 2019) (Fig. 1A).

### Amazon sub-regions

To describe the spatial patterns of species richness in amphibians and their main zones of endemism, we divided the Amazon into 52 drainage sub-basins (Fig. 1B, Suppl. material 1). Each sub-basin was classified using the HydroBASINS structure (https://www.hydrosheds.org/products/hydrobasins), a subset of the HydroSHEDS database. The sub-basins correspond to a set of vectorised polygon layers built with a digital elevation model that provides 12 hierarchically nested divisions of global sub-basins (Lehner and Grill 2013). As the sub-basins tend to decrease in size (km²) from west to east, to avoid very large variations in sub-basin size, we combined three different levels of HydroBASINS to retain only basins with areas > 30,000 km² (following; Oberdorff et al. (2019)). Thus, we selected level five basins for the western region, level 4 for the central region and level 3 for the eastern region. Subsequently, we clipped the basins to the boundaries of the Amazon and recalculated their areas using the field calculator tool in the QGIS software. The 52 sub-basins were then grouped into a single map and named according to their main drainage tributaries (Fig. 1B, Suppl. material 1). All procedures were performed using QGIS 3.16.4 "Hannover" (https://www.qgis.org/pt_BR/site/forusers/download.html).

### Amphibian occurrences

**Digital biodiversity information**

Amphibian known occurrences in the Amazon were obtained from five digitally accessible databases: SpeciesLink (SpeciesLink 2021), (https://splink.cria.org.br), VertNet (VertNet 2021), (http://vertnet.org/), Global Information System on GBIF (GBIF 2021), (www.GBIF.org), Information System on Brazilian Biodiversity - SiBBr (SiBBr 2021), (https://www.sibbr.gov.br) and Authorization and Information System on Biodiversity - SISBIO (SISBIO 2021), (https://www.gov.br/br/pt-br). Data collection was carried out between February and June 2021. Searches of GBIF, Specieslink and VertNet used filters by locality “South America” and taxon “Amphibia”. Searches of SiBBr and SISBIO obtained all records of Amphibia and/or Lissamphibia. These platforms together incorporated species records from 107 sources (collections, museums etc.) spread across America, Europe and Oceania (Suppl. material 2).

**Scientific papers**

Searches for scientific papers were performed using AmphibiaWeb (https://amphibiaweb.org/amphibian/newspecies.html), Google Scholar (https://scholar.google.com.br/?hl=pt) and Web of Science (https://www.webofknowledge.com) platforms with the keywords “Amphibia” AND “Amazon” AND “Checklist” OR “Herpetofauna” OR “New Record” OR “New species”. We considered scientific papers that describe new species, increasing spatial distribution or provide species lists (Check List) published in peer-reviewed scientific journals that presented accurate descriptions of sampled sites, including geographic coordinates. Searches were made for more recent articles (apart from 2010) assuming that data from previous studies would already be incorporated in the previously searched platforms. We compiled 150 scientific papers published in indexed journals (Suppl. material 3).

**Grey literature**

In Google Scholar, we conducted a search for grey literature (Grey Literature 1999) (specifically theses, dissertations, books and technical reports) using the keywords "Amphibia" AND "Amazon" AND "EIA/Rima" OR "Herpetofauna" OR "Monitoring". Through this search, we compiled one thesis, three dissertations, four books and four technical reports (Suppl. material 3) .

**Fieldwork**

Data from 15 years of field research carried out by the authors (2007–2021) in two of the Amazon sub-basins (Tapajós and Xingu Rivers) were also integrated into the dataset. The fieldwork employed sampling methods including visual and auditory active searches, as well as pitfall trapping (according to Vanzolini and Papavero (1967), Campbell and Christman (1982) and Heyer et al. (1994)). Samplings were conducted in standardised plots within permanent modules of the PPBio Biodiversity Research Program (Magnusson et al. 2005). Additionally, searches were conducted at reproductive sites and during occasional encounters. Unpublished data from partner researchers and from fauna rescue operations at the Sinop Hydroelectric Power Plant were also taken into account. Data were further compiled from the Herpetological Collection of the Biological Collection of Southern Amazonia (ABAM) at the Federal University of Mato Grosso (Campus Sinop), which includes unique records for the Amazon, such as the recently-described species *Pristimantispluvian* and *Pristimantispictus* (de Oliveira et al. 2020).

### Data processing

**Validation through taxonomic filter**

All occurrence records were checked for accuracy in taxonomic identification. Records with inaccuracies, such as "cf" (confer), "gr" (group), "aff" (affine), and "sp" (species uncertain), were removed from the database when they could not be confirmed through voucher review in storage collections. Subsequently, the taxonomy of all recorded species was reviewed and updated according to Amphibian Species of the World (Frost 2023), grouping all synonyms under the current valid scientific name.

**Validation through geographic filter**

The geographic coordinates present in the compiled data, whether published on online platforms, peer-reviewed articles or in grey literature, were verified and converted when necessary to decimal degrees in a geographic coordinate system with Datum WGS84 (World Geodetic System 1984), through the conversion tool of the SpeciesLink platform (https://splink.cria.org.br/conversor?criaLANG=pt). Occurrences with geographic coordinates in the original databases described with precision at the level of municipality, state, country or with a radius greater than 20 km, were excluded from the database, aiming to avoid the inclusion of peripheral records to the boundaries of the Amazon.

**Biogeographic filter validation**

After taxonomic validation and geographical coordinate validation, we applied our filter for biogeographic delimitation. In this filter, occurrences were plotted on a map to validate the boundaries within the domain of the Amazon defined according to the World Wildlife Fund (https://services2.arcgis.com/j80Jz20at6Bi0thr/arcgis/rest/services/Amazon_Rainforest/FeatureServer), using the QGIS software (address). Subsequently, all species that passed through the three filters were individually evaluated in terms of their currently known distribution. For this, we relied on the authors' experience in the area and consultations with anonymous expert partners to diagnose species of dubious occurrence in the Amazon. In this assessment, we found a total of 220 species judged to be dubious in their occurrence in the Amazon Region by the evaluators (Suppl. material 4). Subsequently, each species in this set was individually evaluated in three biodiversity data sources, Amphibian Species of the World (Frost 2023), AmphibiaWeb (AmphibiaWeb 2023) and the International Union for Conservation of Nature IUCN (IUCN 2023), as well as publications attached to these platforms. In this evaluation, 82 species (Supplementary Material 5) were excluded from our database for not having any mention of their distribution in the Amazon in all the sources surveyed, with the remaining species being incorporated into our data.

### IUCN assessments, statistical analysis and cartographic projections

We compared species richness, endemism and the number of records (sampling effort) amongst the Amazonian countries, as well as the temporal distribution of studies and the contribution of different sources to the final database (number of species, occurrences and sampled locations). Observed species richness (from records) and estimated (Jackknife Estimator 2) were evaluated by the species accumulation curve considering years as samples, using the R software version 3.6.2 (RStudio Team 2020) with the 'poolaccum' function from the vegan package (Oksanen 2019). Cartographic projections of species richness with grids of 50 x 50 km were performed in the RStudio environment. The relationship between sub-basin area and species richness was assessed by a Generalised Linear Model (GLM) with Poisson distribution. Similarity in species composition amongst the 52 sub-basins was evidenced by cluster analysis using the Jaccard Index for species richness data.

For descriptions of species distribution in our database, we evaluated our species set, based on three globally comprehensive and updated biodiversity platforms: Amphibia Species World (Frost 2023), AmphibiaWeb (AmphibiaWeb 2023) and the International Union for Conservation of Nature - IUCN (IUCN 2023). We classified species according to the biogeographic regions in which they were recorded on these platforms and in articles attached to them, as follows: AM - Amazon, AF - Atlantic Forest, AN - Andes, CA - Caatinga, CE - Cerrado, CH - Chaco, CB - Caribbean, PA - Paramos, PM - Pampas, PN - Pantanal and SPA - Subparamo.

Endemism was defined for species reported exclusively in the Amazon biome on both platforms. To define the number of endemic species within each sub-basin, we considered only Amazon endemic species, quantifying them for each sub-basin. Using software QGIS 3.16.4 "Hannover" (https://www.qgis.org/pt_BR/site/forusers/download.html), we clipped occurrences referring to Amazon endemic species using sub-basin shapefiles and subsequently compared species lists to locate species unique to each biogeographic region (sub-basins).

Maps were constructed with polygons representing drainage sub-basins, including colour palettes ranging from white to dark green representing values of richness and endemism in each polygon to highlight sub-basins with high species richness and endemism. Species were classified according to extinction risk following IUCN categories (IUCN 2023) as: Critically Endangered (CR), Endangered (EN), Vulnerable (VU), Near Threatened (NT), Least Concern (LC), Data Deficient (DD) and Not Evaluated (NA).

## Results

**General data**

Our initial database consisted of 902,986 occurrences obtained from four research sources. Online platforms contributed the majority, with 856,985 occurrences (approximately 95%). Specifically, GBIF accounted for 324,582, SiBBr for 234,203, SpeciesLink for 180,625, VertNet for 68,570 and SISBIO for 49,005 occurrences. Grey literature, peer-reviewed articles and personal data contributed 39,045 (~ 4.3%), 3,496 (~ 0.4%) and 3,460 (~ 0.4%) occurrences, respectively (Suppl. material 5). Amongst these occurrences, 220,326 (~ 24%) were excluded due to lack of taxonomic refinement at the species level (filter 1 - taxonomic refinement). The absence of geographic coordinates in the occurrences (filter 2 - geographic coordinate deficits) led to the exclusion of 356,309 (~ 39.5%) of the data and another 165,708 (~ 18.4%) occurrence data were eliminated because they were outside the established limits for the Amazon (filter 3 - biogeographic limits). Thus, 742,343 occurrences (~ 82%) of the initial occurrence data were excluded due to not meeting the three filters established here. The data sources with the lowest rate of validated records after the three filters were SiBBr (1.6%) and VertNet (5.5%). In the end, our database consisted of 160,643 occurrences (Suppl. material 5).

### Amphibian diversity patterns in the Amazon

**Figure 2.**
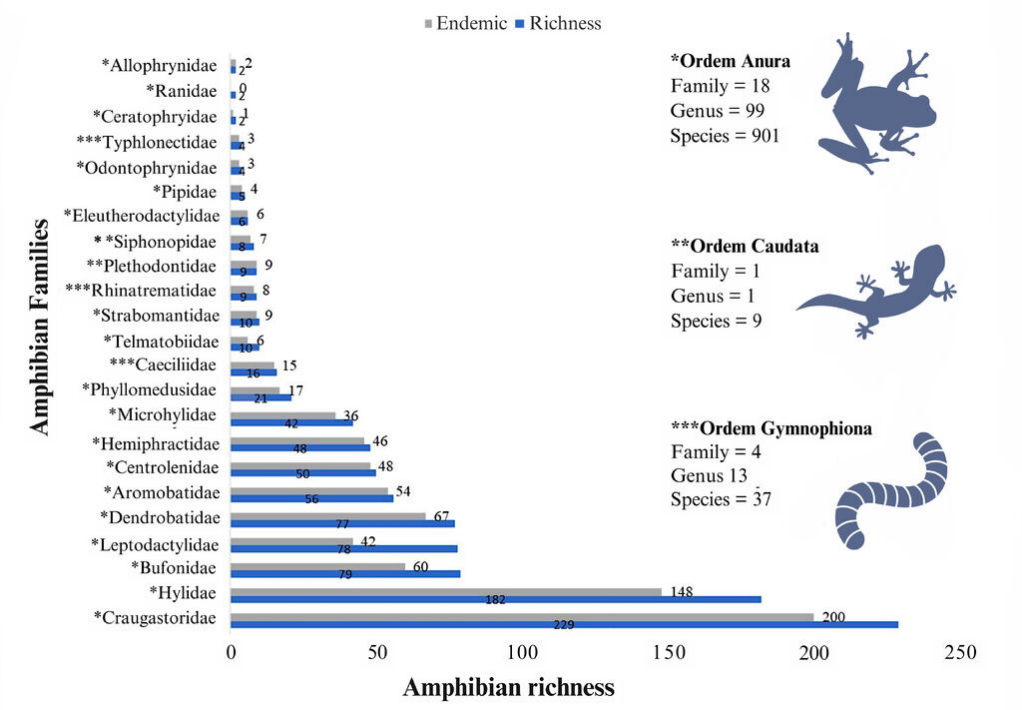
Distribution of species richness and endemism amongst amphibian families recorded in the Amazon.

The 160,643 occurrences selected from this study’s database are distributed across the three orders of amphibians, comprising 23 families, 113 genera and 947 species, of which 775 species (81.8%) are endemic to the Amazon (Suppl. material 6). Amongst the three orders, Anura is represented by 18 families, 99 genera and 901 species; Gymnophiona by four families, 13 genera and 37 species; and Caudata by one family, one genus and nine species. The 10 most representative amphibian families include 864 species (91%) of the total for the Amazon (Fig. 2). The ten species with the highest number of records were *Rhinellamarina* (Linnaeus, 1758) (966 records), *Osteocephalustaurinus* Steindachner, 1862 (806), *Rhinellamargaritifera* (Laurenti, 1768) (737), *Scinaxruber* Laurenti, 1768 (653), *Adenomeraandreae* Müller, 1923 (578), *Pristimantisfenestratus* Steindachner, 1864 (514), *Boanaboans* (Linnaeus, 1758) (476), *Trachycephalustyphonius* Linnaeus, 1758 (463), *Allobatesfemoralis* (Boulenger, 1884) (458) and *Leptodactyluspetersi* (Steindachner, 1864) (458). However, 493 species (52%) showed a low number of occurrences (< 20) and registered locations (≤ 5) for the Amazon, with 198 singleton species and 106 doubleton species regarding the number of occurrences (Suppl. material 6).

**Figure 3.**
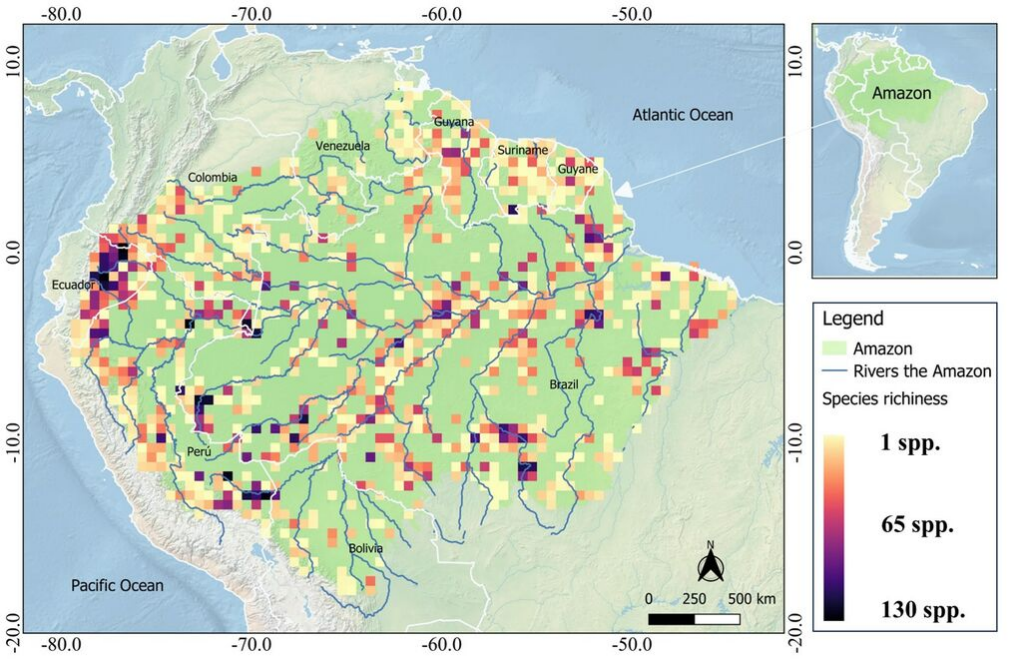
General distribution of amphibian species richness in the Amazon represented by cells with a resolution of 50 x 50 km.

The species richness of amphibians in the Amazon is high, totalling 947 species, which represents approximately 11% of the global species richness. However, this richness is not evenly distributed in space. Some areas have high species richness, with over 65 species per cell (50 x 50 km grid), while extensive regions have low species richness, with fewer than 33 species per cell (Fig. 3). Cells with high richness are concentrated in western regions, mainly in Ecuador, in the hydrographic basins of Marañon, Patumaio and Napo; in western Brazil, in the lower portion of the Patumaio, Purus and Juruá Basins; in southern Peru and south-western Bolivia, in the Beni Basin; as well as in some cells in the central region of the Brazilian Amazon, in the Amazon River Basin near Manaus, in the State of Amazonas and in the eastern region, mainly in the Araguari, Belém and lower Xingu Basins, both in the Brazilian State of Pará. On the other hand, the entire northern, north-western, southern and south-eastern regions have low richness per cell (Fig. 3).

### Richness pattern and geopolitical and biogeographic divisions

**Figure 4.**
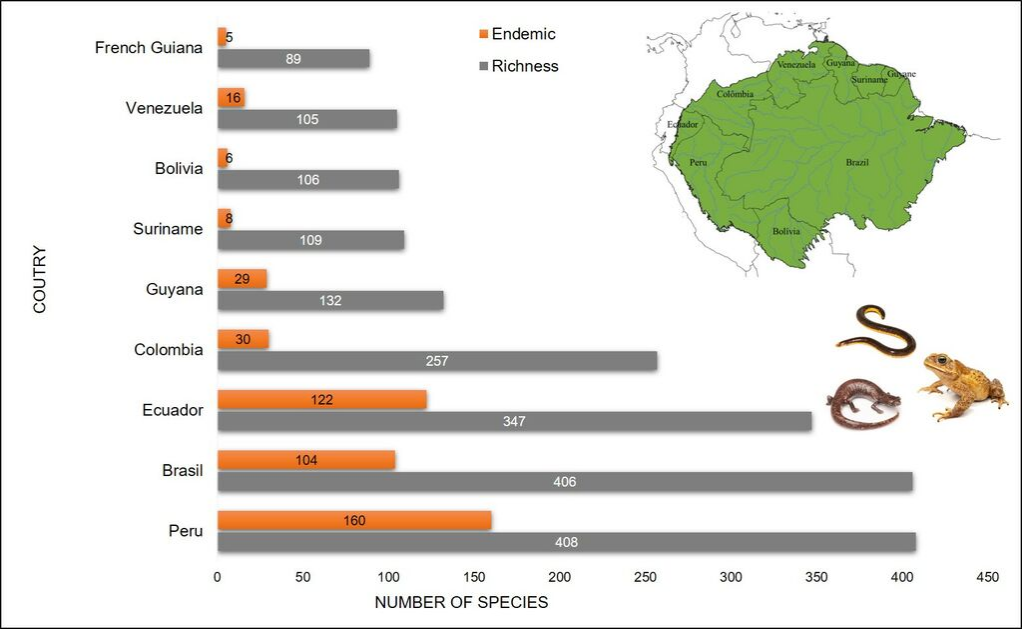
Distribution of amphibian species in the Amazon. Richness (blue) and endemism (grey) by country (A) and location of countries in the Amazon (B).

Species richness and endemism, as well as the number of records, differs amongst South American countries (Fig. 4, Suppl. material 7). Peru, Brazil, Ecuador and Colombia have the highest richness and endemism, with up to three times more species when compared to the other countries (Fig. 4). Sampling effort is also biased towards Brazil, Ecuador and Peru (Suppl. material 7), which together account for 81% of the sampled sites and 87% of the total number of occurrence records.

**Figure 5.**
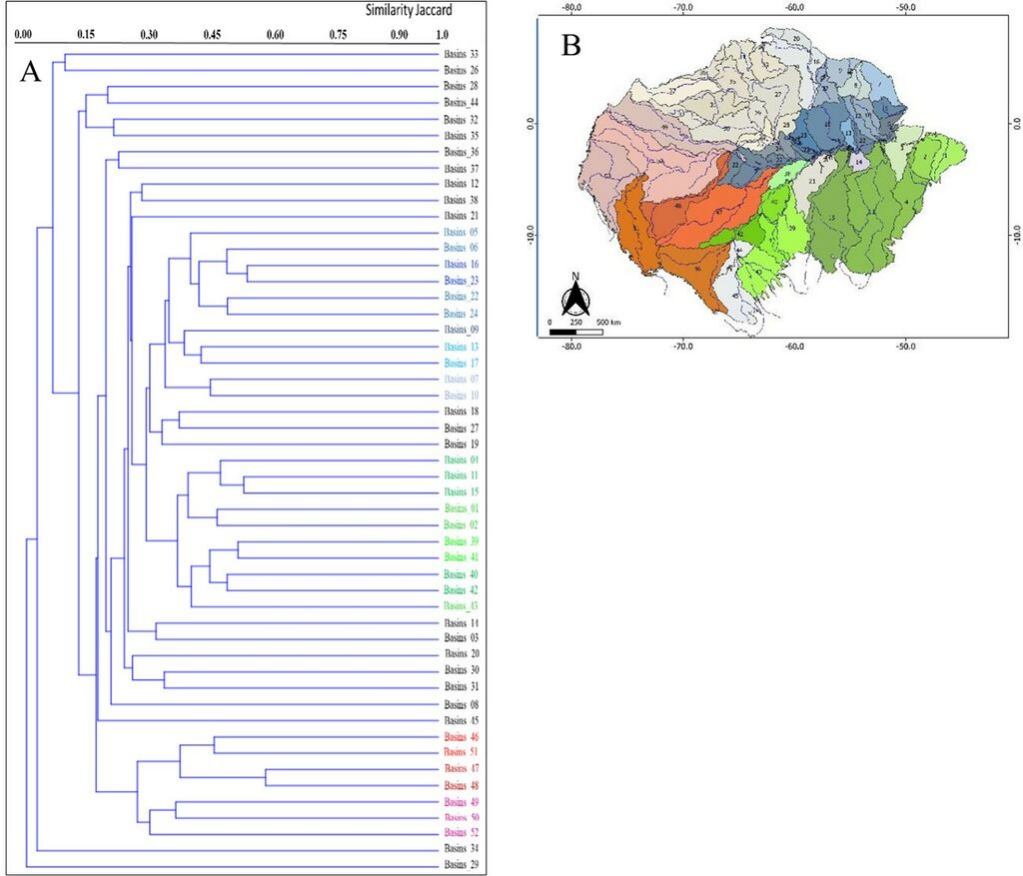
Similarity in amphibian species composition amongst the 52 Amazonian drainage sub-basins. **A** - Clustering based on species similarity amongst sub-basins (Jaccard Index); **B** - Location of the drainage sub-basins in the Amazon Region. Light grey shades represent sub-basins with similarity less than 40%. Shades of blue, green and red represent similarity greater than 40%. Names of the sub-basins are detailed in Suppl. material 1.

Overall, the Amazonian River drainage sub-basins showed high species richness of amphibians, with an average of 86.9 ± 72 (1 to 351) species per sub-basin (Suppl. material 8) and low similarity in species composition (Fig. 5A). The results indicate positive relationships between sub-basin size and richness (z = 41.55, p < 0.001) and endemism (z = 25.44, p < 0.001) (Suppl. material 9). Sub-basins generally exhibit low similarity (< 40%) in species composition (Fig. 5A). The only basins with greater than 50% similarity are the middle portion of the Purus and Juruá with 59%, Uatumã and Araguari with 54%, Uatumã and Trombetas with 54%, Tapajós and Xingu with 53%, Ji-Paraná and Roosevelt with 52% and middle Purus and middle Madeira with 51% (Fig. 5A). The findings also suggest the existence of at least four groups of sub-basins with similar species composition, the first consisting of the drainage from the left channel of the Amazon River to the Oiapoque, encompassing part of the drainage of the Negro and Trombetas Rivers. The second is formed by the southeast sub-basins, the right channel of the Amazon River formed by the drainage of the Tapajós, Xingu, Capim Guama and Gurupi Rivers. The third is the central-southern region formed by the middle portion of the Madeira River and the fourth, located further east, is formed by the drainage of the Purus and Juruá Rivers. On the other hand, there is an extensive area of low similarity (< 40%) mainly concentrated throughout the northwest extension of the Amazon (Fig. 5B). Therefore, latitudinal and longitudinal separation was observed in the regions in the formation of clusters (Fig. 5B).

**Figure 6.**
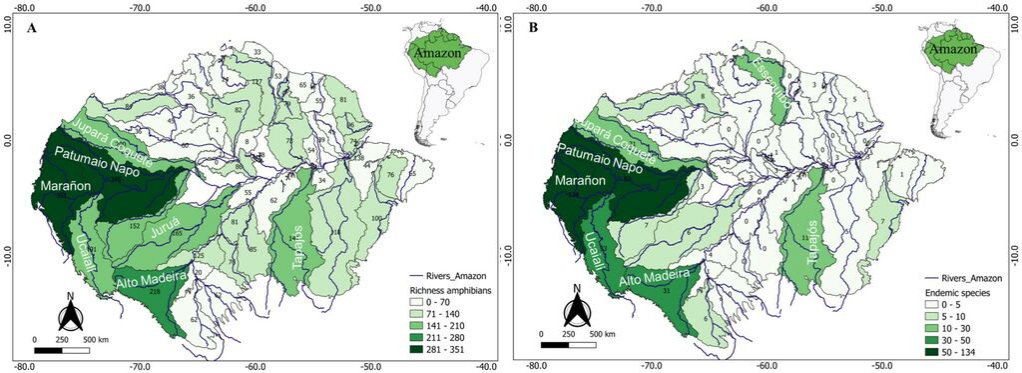
Number of occurrences of amphibian species in the biogeographic divisions of the Amazon (drainage sub-basins). **A** - Species richness; **B** - Endemic species.

The richness and endemism of amphibian species per sub-basin exhibit a biogeographically biased pattern (Fig. 6). The western portion of the Amazon showed higher values for both metrics compared to the eastern portion. The Marañon, Patumaio Napo, Ucaiali, Juruá, Jupará Coquete, Upper Madeira and Tapajós sub-basins stand out for species richness, all with more than 140 species (Fig. 6A), while Marañon, Putumaio Napo, Jupará Coquetá, Ucaiali, Alto Madeira, Essequibo and Tapajós stand out for endemism, all with more than 10 endemic species (Fig. 6B).

### Data Temporal increment

**Figure 7.**
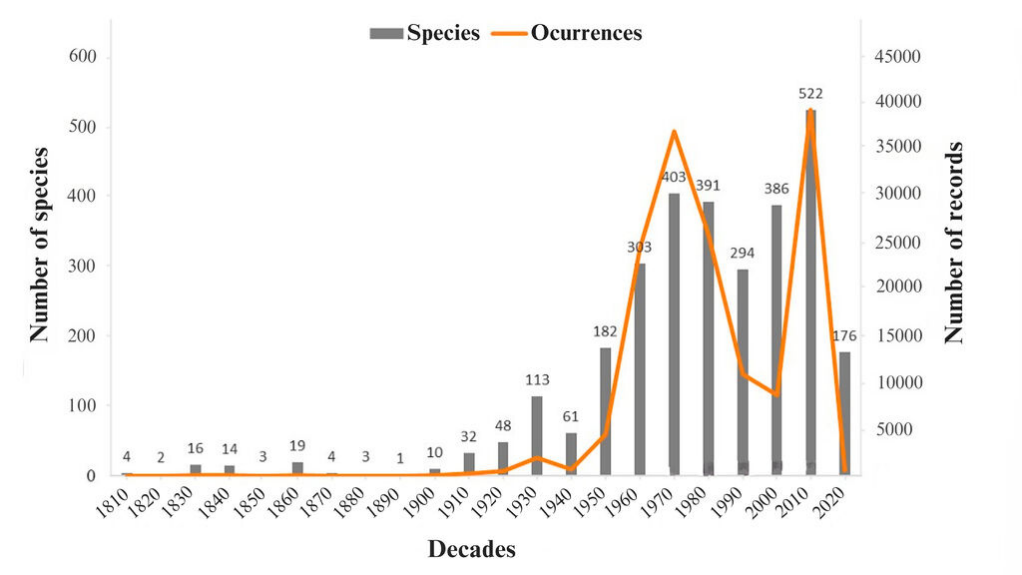
History of occurrence records (orange line) alongside the corresponding number of species (grey bars) compiled from data on amphibians recorded in the Amazon. Data are presented as the sum of occurrence records and number of species for each decade.

**Figure 8.**
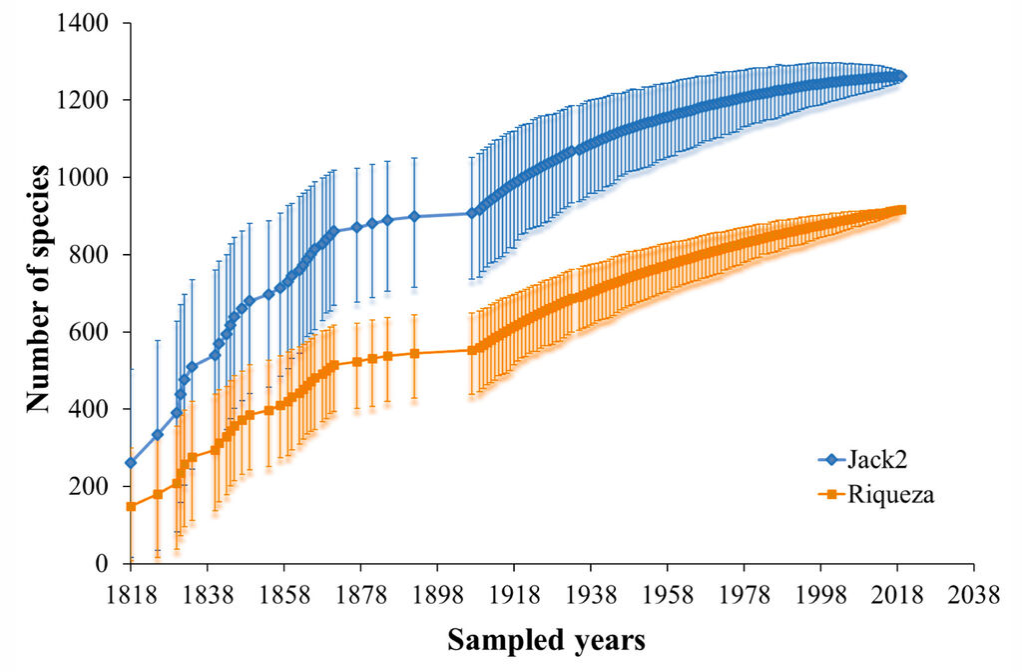
Observed (presence records) and estimated (Jackknife 2) richness of Amazonian amphibians.

Of the total occurrences of amphibians in our database (160,643), 95% refer to the year in which they were sampled. The first occurrence was dated 1818 for four species. Both species richness and the number of occurrences remained low during the 19^th^ century, increasing considerably throughout the 20^th^ century, mainly from the 1950s onwards (Fig. 7). The rarefaction results highlight an increase in species records over time and the observed richness (947 species) represented 75% of the estimated richness (Jackknife 2; 1,262 species), indicating that the Amazon is under-sampled and its richness is underestimated (Fig. 8).

### Conservation Status

**Figure 9.**
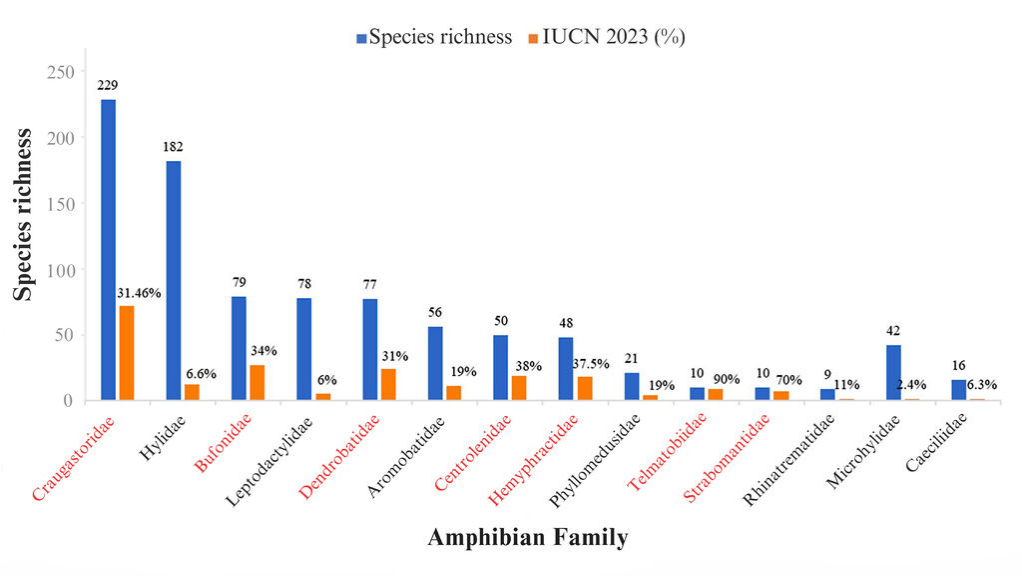
Amphibian species richness by family, along with the respective proportions of species categorised under different levels of extinction risk according to the IUCN Red List of Threatened Species (IUCN 2023). Families highlighted in red present more than 30% of the species classified in the extinction risk categories (CR, VU, EN), according to an assessment by IUCN (IUCN 2023).

Of the 947 amphibian species recorded for the Amazon, 841 species (88.7%) were assessed by the International Union for Conservation of Nature - IUCN (IUCN 2023), while 39 species (4.1%) were not assessed (NA) and 68 species (7.2%) were considered Data Deficient (DD). Of the total Amazonian amphibian species, 589 species (62%) were classified as Least Concern (LC). However, 208 (22%) are classified as threatened with extinction, with 48 species (5.1%) Critically Endangered (CR), 104 species (11%) Endangered (EN) and 56 species (5.9%) Vulnerable (VU). Another 44 species (4.6%) are Near Threatened (NT) (Suppl. material 6). Of the 23 amphibian families assessed by the IUCN, 14 presented some species at risk of extinction (CR, EN or VU). Of these, seven families had over 30% of their species in these threat categories, with Strabomantidae presenting 70% and Telmatobiidae 90% of threatened species (Fig. 9).

### Representativeness of search sources

**Figure 10.**
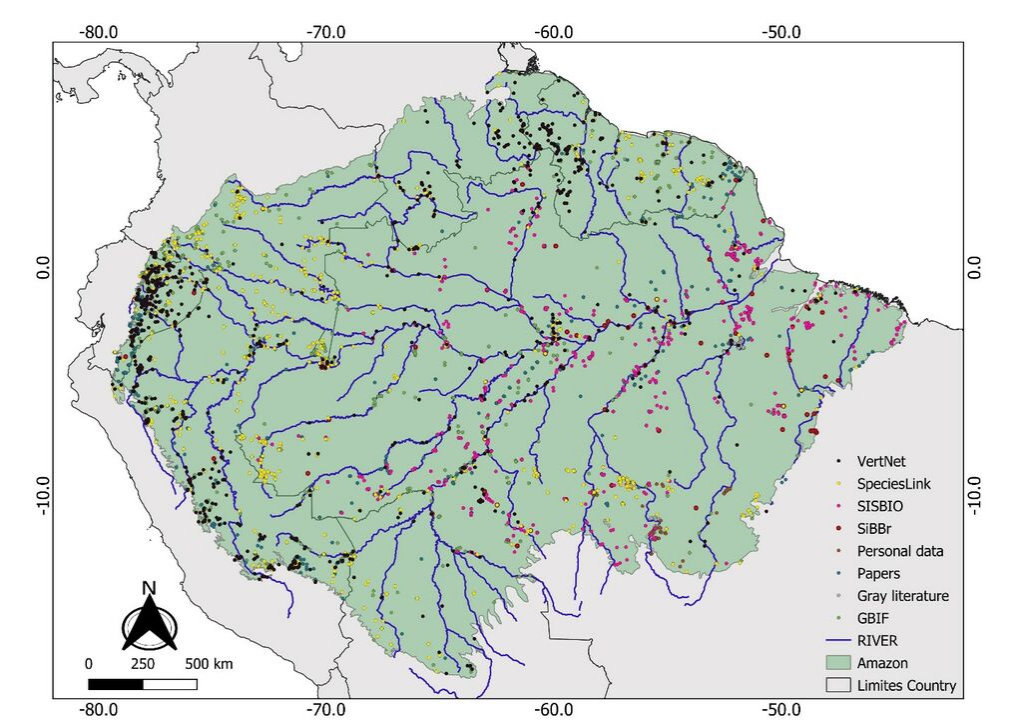
Complementarity of the different data sources researched on amphibian occurrences in the Amazon. The sum of the recording rate in terms of number of occurrences, species richness and sampled locations is available in descending order from the highest contributing source to the lowest contributing source in Suppl. material 10.

The varied data sources used in this study contributed differently, but complementarily to the integrity of the compiled dataset (Fig. 10). The greatest contribution of species occurrences came from the GBIF, VertNet and SpeciesLink databases, totalling 75% of all records and 83.8% of all species. However, these three databases together represent just over 54% of the sampled locations (Suppl. material 10). Although the data from SiBBr added just over 1% to species richness and 5% of the number of records, they made a significant contribution (~ 25%) to the number of sampled locations. Published scientific works also deserve attention because this source accounted for approximately 7% of sampled locations and 12% of the total number of species, although it added just over 2% to the number of records (Suppl. material 10).

## Discussion

### Amphibian diversity

Our results reveal that the Amazon houses approximately 11% of the currently described amphibian species (Frost 2023), establishing itself as the tropical rainforest with the highest amphibian diversity in the world, surpassing significant biodiversity hotspots such as the Southeast Asian rainforests (with ~ 700 species) (Das and Dijk 2013, Pratihar et al. 2014) and the Atlantic Forest of South America (with 625 species) (Vancine et al. 2018). In addition to this documented abundant diversity, our estimate (Jaccard Index) suggests a richness exceeding 1200 species, indicating that the number of amphibian species in the Amazon, although already considerable, is still significantly underestimated and poorly documented (Fouquet et al. 2007, Funk et al. 2011, Ferrão et al. 2016, Motta et al. 2018, Stropp et al. 2020).

The high biological diversity found in the Amazon was shaped over millions of years as a result of a combination of factors involving bioclimatic heterogeneity, complex landscape features and multiple biogeographical barriers (Dexter et al. 2017, Antonelli et al. 2018, Musher et al. 2019, Carvalho et al. 2023, Almeida-Silva et al. 2024). Just as these factors have fostered significant diversification in the Amazon, structural, economic and social factors, such as inaccessibility, anti-environmental policies, underdeveloped scientific infrastructure and restrictive legislation for data access and dissemination in Indigenous Lands, are some of the factors responsible for the knowledge deficits still observed in the Amazon Region and other tropical forest domains around the world (Tuomisto 1998, Dos Santos et al. 2015, Oliveira et al. 2016, Magnusson et al. 2018, Carvalho et al. 2023).

The Amazon not only presents a remarkable species richness, but also a high rate of endemism, surpassing 82% of all amphibian species recorded in this study. This figure aligns with estimates by Vacher et al. (Vacher et al. 2020), who identified similar levels of endemism for amphibians in the eastern Guiana Shield. This rate of endemism exceeds that observed for amphibians in the Atlantic Forest, which is recognised as one of the major biodiversity hotspots in the world (Vancine et al. 2018). Although species richness is traditionally the most investigated metric as a central component of biological diversity (Schall and Pianka 1978, Pearson and Cassola 1992, Myers et al. 2000, Nneji et al. 2023), the rate of endemism serves as an important indicator of species specificity and vulnerability to extinction threats (Kraus et al. 2023, Shipley and McGuire 2023). Endemic species tend to be restricted to limited geographic areas (Kraus et al. 2023, Nneji et al. 2023, Shipley and McGuire 2023), often used to delineate biogeographic regions (Morrone 2014) and establish priorities in conservation plans (da Silva et al. 2005, Adams and Sandbrook 2013, Nori et al. 2016, Noroozi et al. 2018). Endemic species and those with restricted distributions, such as the majority of species documented in this study (52% with less than five occurrence points) (Suppl. material 6), are ecologically and evolutionarily distinct, presenting specific ecological niches, making them more vulnerable to extinction (Shipley and McGuire 2023). The high rate of endemism in the region, combined with the limited biological knowledge about most species, highlights the importance of intensifying research on biodiversity in the Amazon, especially given the rapid advance of forest degradation and land use in the region, resulting in species loss (Fearnside 2015, Winemiller et al. 2016, Latrubesse et al. 2017, Stropp et al. 2020). Furthermore, resources need to be directed towards conservation, prioritising threatened, little/poorly known and rare species due to their greater vulnerability to extinction (Nori et al. 2016, Kraus et al. 2023).

### Spatial patterns

The high amphibian diversity in the Amazon, characterised by a large number of species (richness) and a high index of biome-exclusive species (endemism), exhibited a strongly-biased distribution pattern, with increased richness and endemism in the east-west longitudinal direction. Although the sub-basin area is positively correlated with species richness, greater amphibian diversity occurred in western Amazonia, primarily in Bolivia, Colombia, Ecuador and Peru, from the Beni River Basin in Bolivia to the Coquetá River Basin in Colombia. This pattern persisted even after standardising the study area in 50 x 50 km grid cells (Fig. 3). Similar patterns have been observed in several studies reporting high richness for western Amazonia in different taxa, such as passerines (Rangel et al. 2018, Crouch et al. 2019), mammals (Rangel et al. 2018), plants (Alvez-Valles et al. 2018, Rangel et al. 2018, Blundo et al. 2021), amphibians (Fonseca et al. 2022) and fish (Oberdorff et al. 2019). The difference in richness and species composition in the Amazon is related to a set of historical and spatial factors that have shaped the current Amazonian environment over millions of years, providing differences in heterogeneity and climatic stability across space and time (Hoorn et al. 2010, Rangel et al. 2018, Rahbek et al. 2019).

Western Amazonia, encompassing specific areas of Bolivia, Colombia, Ecuador and Peru, is influenced by the presence of the Andes Mountains. This region features a high altitudinal gradient with significant topographic variation, providing greater climatic and environmental heterogeneity, which favours speciation and species co-existence (Rosenzweig 1995, Rangel et al. 2018, Oberdorff et al. 2019). The progressive uplift of the Andes, which began about 25 million years ago (Gregory-Wodzicki 2000), has had a significant impact on the regional climate, being crucial in shaping the Amazonian landscape, reconfiguring drainage patterns and creating a vast influx of oceanic sediments into the Amazon (Hoorn et al. 2010, Rahbek et al. 2019). Throughout its historical uplift, it is believed that the environmental and climatic heterogeneity of the Andes has driven species diversification (Rangel et al. 2018). The fragmented environmental heterogeneity of the Andes, influenced by topographic complexity and altitudinal climatic gradients (altitude valleys), has favoured speciation, acting as "cradles" (areas of especially rapid species origin), while also serving as a climatic refuge against extinction, in contrast to lower altitudes that have acted as "museums" (areas of long-term species persistence). Conversely, regions with lower altitudes tend to act as "graveyards" for biodiversity, due to greater climatic fluctuations, resulting in particularly high extinction rates (Rangel et al. 2018, Rahbek et al. 2019).

Since amphibians generally have low mobility, topographic heterogeneity can act as a geographical barrier, promoting higher speciation rates, with the reduced size of species' habitats leading to a higher species-area relationship (Godinho and Da Silva 2018). Thus, beneficial and well-developed climates over time, such as those found in Andean altitudinal valleys, can contribute to resource stability and, consequently, to the persistence of amphibian species over time (Tedesco et al. 2017, Rangel et al. 2018, Oberdorff et al. 2019), increasing the stochastic richness and endemism observed in these regions.

In the Brazilian Amazon, areas of highest richness and endemism are located in the western region, primarily within the Juruá and Purus River Basins, likely due to Andean influence as previously mentioned. Another region with high richness and endemism is located in the southeast, within the Tapajós River Basin. The Tapajós River lies in the biogeographical transition zone between the Solimões sedimentary basin (western margin) and the Brazilian Shield (eastern margin) (Sombroek 2000, Moraes et al. 2016). This region exhibits species overlap, where the diverse fauna of the dense forests of western Amazonia, influenced by the Andes and the sedimentary basin, interacts with elements of the drier forests found in eastern Amazonia (Amazon-Cerrado transition) (Moraes et al. 2016). The Tapajós River Basin contains biotic components from both western and eastern Amazonia, forming a secondary contact zone (Maximiano et al. 2020), which may explain the observed high species richness. Overall, the observed pattern of endemism and species richness highlights the importance of historical, topological and climatic factors in shaping the distribution of Amazonian amphibians (Godinho and Da Silva 2018, Maximiano et al. 2020).

In addition to the projected pattern of increasing species richness and endemism in the east-west longitunal direction observed in the Amazon, we observed low similarity in species composition between Amazonian sub-basins, particularly in the northwest and north regions. Furthermore, there was a strong separation in species composition between basins separated latitudinally by the Amazon River and longitudinally by the Tapajós, Madeira, Purus and Juruá Rivers (Fig. 5B). Other studies have shown similar patterns of community similarity in Amazonian biodiversity for amphibians (for example, Funk et al. (2007), dias‐Terceiro et al. (2015), Moraes et al. (2016), Godinho and Da Silva (2018)), birds (Oliveira et al. 2017, Maximiano et al. 2020), mammals (Bonvicino et al. 2003, Boubli et al. 2008, Boubli et al. 2015) and plants (Nazareno et al. 2017). This finding suggests that major Amazonian rivers may act as semi-permeable biogeographic barriers (limiting the passage of different species in numbers of genetically viable individuals to establish themselves on both river banks (Santorelli Jr et al. 2018, Junior et al. 2022). Furthermore, other environmental factors such as environmental heterogeneity, river width, river formation history, amongst others, can affect community structuring depending on the spatial scale tested (Jones et al. 2006, Juen and De Marco 2012, Santorelli Jr et al. 2018, Alves-Martins et al. 2019, Maximiano et al. 2020, Junior et al. 2022) and the characteristics of the taxonomic groups and species studied (Moraes et al. 2016, Maximiano et al. 2020.

It is also important to highlight that the transition of Amazonian rivers underwent significant changes during the Miocene geological period (between 23 and 5 million years ago), primarily due to the aforementioned uplift of the Andes (Hoorn and Vonhof 2006, Hoorn et al. 2010, Oberdorff et al. 2019). These changes in the configuration of the Amazonian landscape may have played a crucial role in the current patterns of species distribution, richness and endemism (Shephard et al. 2010, Latrubesse et al. 2017, Rangel et al. 2018, Oberdorff et al. 2019, da Silva-Caminha et al. 2020, Espinosa et al. 2021, Leandro et al. 2022). Therefore, we understand that rivers are disproportionately influential in shaping species distribution patterns, which directly depend on their historical (formation period) and physical characteristics (e.g. size, width, depth, geological formation, amongst others) (Nazareno et al. 2017, Santorelli Jr et al. 2018). Additionally, the ecological, morphological and physiological characteristics of species also influence spatial community distribution patterns (dias‐Terceiro et al. 2015, Moraes et al. 2016, Nazareno et al. 2017, Godinho and Da Silva 2018, Maximiano et al. 2020). Thus, the barriers formed by rivers are not the only possible explanation for community dissimilarity (Godinho and Da Silva 2018, Moreno et al. 2024). Therefore, we encourage future investigations to precisely unravel the factors that have shaped and are shaping distribution patterns across different scales in the Amazon.

### Temporal scale of data

The first scientific expeditions into the Amazon took place more than 150 years ago (19^th^ century), following the course of the Amazon River (Nonato and Pereira 2013). The oldest record in the present database is from 1818, which was followed by a long period of stagnation in both the number of records and number of species, extending for about 100 years until the beginning of the 20^th^ century. Later, beginning in the middle of the 20^th^ century, there was a significant increase in scientific knowledge of Amazonian biodiversity. This increase was encouraged, in part, by the institutionalisation of the Museu Paraense Emilio Goeldi and the creation of the Instituto Nacional de Pesquisa da Amazônia INPA (Faulhaber 2005) and the Universidad Nacional de La Amazonia Peruana, amongst other important research centres. More recently, numerous inventories and fauna monitoring reports for infrastructure, mining and energy production works throughout the Amazon have contributed to expanding knowledge about its biodiversity (Ávila and Kawashita-Ribeiro 2011, Vaz-Silva et al. 2015). Nonetheless, knowledge of Amazonian biodiversity remains underestimated, as evidenced by the projected amphibian richness of 1261 species, that only about 75% of what is expected to exist has been described and catalogued in the database assembled here.

Guerra et al. (2020) highlight the Amazon as amongst the biomes with the lowest number of described species when compared to other South American biomes and, therefore, more species can be expected to be discovered with increased sampling and taxonomic efforts. Many other studies have also suggested that the biodiversity of Amazonian amphibians is severely underestimated (e.g. Funk et al. (2011), Ferrão et al. (2016), Vacher et al. (2020)). Knowing the true richness of amphibians has been a great challenge for researchers, but it is also crucial for establishing conservation priorities and understanding biogeographical patterns.

### Conservation status

Amphibians are the group of vertebrates with the highest percentage of species considered at risk of extinction globally at 41% (IUCN 2023). For the Amazon, we found 22% of amphibian species classified as at some risk of extinction: however, another 11% have not yet been evaluated or are considered Data Deficient, that is, with little or no ecological and natural history data to support their placement any threat category. When compared to the IUCN assessment (IUCN 2018), only 17% of species were considered threatened; therefore, there was an increase of 5% between assessments. Natural history studies, in association with assessments by herpetologists, will provide more consistent threat assessments, with the number of species in threatened categories expected to increase.

The present study found that 52% of Amazonian amphibian species have a known distribution restricted to just five or fewer sampling points. Given the high rate of endemism of Amazonian amphibians (~ 82%), the low number of sample points at which many species have been recorded in the present database could suggest reduced distributions for these species, aggravating their extinction risk with habitat loss. Furthermore, the Amazon Region is highly vulnerable to climate change, mainly due to the large number and proportion of species that are sensitive to changes in habitats and abiotic factors (Foden et al. 2013, Luedtke et al. 2023). Given the high rate of forest cover loss, in addition to soil, air and water contamination by agricultural, industrial and urban waste and the construction of large infrastructure projects (hydroelectric plants, transmission lines, road paving etc.), the risk of extinction is high, especially for the more sensitive species (Collins and Crump 2009, Luedtke et al. 2023). Furthermore, it is possible that many species may be going extinct without even being known to science.

Special attention should be given to more sensitive species that have been suffering serious population declines, such as those of the genus *Telmatobius* (Angulo 2008) and *Atelopus* (La Marca 2005). Some amphibians are of high priority due to their susceptibility to the invasive fungal pathogen *Batrachochytriumdendrobatidis* (La Marca 2005, Toledo 2009, Luedtke et al. 2023). The situation may be even more drastic for salamanders (*Bolitoglossa* spp.) and caecilians (Gymnophiona) since knowledge about the biology of most species is lacking (Azevedo-Ramos and Galatti 2002, Semlitsch et al. 2017).

### Sampling deficits

The advances in prediction and computational simulation models enable us to trace global biodiversity patterns, identify and monitor species displacement (Michelot et al. 2016, Borowicz et al. 2019, Song et al. 2023) and further predict the impacts of human activities and climate change on ecosystems (Hopkins 2007, Dos Santos et al. 2015, Stropp et al. 2020, Albuquerque et al. 2021, Carvalho et al. 2023) However, it is worth noting that the application and robustness of these models depend on the quality and quantity of the data used to feed them and that ignoring these variables will lead to misconceptions of the observed patterns (Ballesteros‐Mejia et al. 2013, Anderson et al. 2020.

The result of the present study highlights strong limitations in primary biodiversity data available from different sources (Suppl. material 5). Over 80% of the initially accessed data could not be used due to some deficit, with the two most limiting factors being the absence of taxonomic refinement (Linnaean deficit) and the lack of geographic coordinates (Wallacean deficit) (Hortal et al. 2015). Although online platforms contributed to the majority of occurrences in our database (Suppl. material 5), important data providers for these platforms, such as the Museu Paraense Emílio Goeldi (MPEG), the Instituto Nacional de Pesquisa da Amazônia (INPA) and the Museu de Zoologia da Universidade de São Paulo (MZUSP), exhibited high Linnaean and Wallacean deficits in their occurrence records, making their use here unfeasible. These two deficits are consistently identified as a barrier to the use of museum and biological collection biodiversity data (Lomolino 2004, Whittaker et al. 2005, Beaman and Cellinese 2012, Hortal et al. 2015, Peterson et al. 2015, Anderson et al. 2020). We therefore emphasise the great importance of recording geographical coordinates during field sampling, as well as hiring technical biologists in zoological collections for better taxonomic validation of species, digitisation and dissemination of records on online platforms or scientific publications that facilitate their use by the scientific community and decision-makers in conservation.

Another important action would be to require scientific disclosure of data collected during environmental impact studies. Licensing studies conducted for potentially polluting activities, such as infrastructure works, energy generation and mining, generate large databases, many of them with high spatial-temporal sampling effort (Vaz-Silva et al. 2015). However, there is little or no incentive for companies and/or local environmental agencies to make these data available and disclose them. In the present study, for example, we were unable to access species registration data for the majority of operating or under-installation hydroelectric plants and mines in the Amazon, data that could enrich our knowledge about remote areas of the Amazon where these works/activities have been operating.

In this sense, we praise important cooperation initiatives between researchers such as the "Censo da Biodiversidade" developed by the Museu Paraense Emilio Goeud, which aims to validate and disseminate biodiversity records in the Brazilian Amazon (Hoogmoed and Galatti 2023). We would like to encourage other institutions and researchers to become involved in similar projects, enabling an adequate completeness of knowledge about Amazonian biodiversity.

## Conclusion and future perspectives

(1) The Amazon is the biome with the greatest richness in amphibian species in the world, which has been shaped over millions of years by factors such as bioclimatic heterogeneity, landscape complexity and biogeographic barriers. Considering that our estimates suggest a greater richness (1,200 species) than currently known (947 species), the knowledge about this diversity is still underestimated both in relation to the distribution of species (Wallacean shortfall) and the number of species described (Linnean shortfall). The inaccessibility of many areas, political conflicts and lack of scientific investment are factors that hinder the advancement of knowledge about biodiversity in the Amazon.

(2) Our study reveals a longitudinal gradient in richness and endemism, with greater diversity observed in the western region, influenced by the Andes Mountains. The large Amazon rivers (e.g., Amazonas, Juruá, Madeira, Purus, Tapajós) acted as biogeographic barriers, influencing the low similarity in species composition between sub-basins.

(3) Despite the increase in scientific knowledge about Amazon amphibians in recent decades, unfavorable political changes threaten research and biodiversity conservation. According to an IUCN assessment (2023), around 18% of Amazonian amphibian species are threatened with extinction, while 26% of species lack adequate assessment, which represents significant gaps in the knowledge of their biogeographical distributions, population dynamics and history. Furthermore, the high rate of endemism (~ 82%) and species with low abundance and restricted distribution (~ 52%) observed in this study, may have their populations reduced by emerging anthropogenic and climate changes and may become extinct in the future.

(4) This study represents a significant effort to understand the biogeographic patterns of amphibian diversity in the Amazon, directing future research to uncover the ecological and evolutionary mechanisms that drove current biogeographic patterns, as well as assisting in biodiversity conservation measures in the Amazon.

## Supplementary Material

17EEC580-4514-5373-8901-9925BB3D7BE2 10.3897/BDJ.12.e109785.suppl1 Supplementary material 1 Map showing the 52 drainage sub-basins within the Amazon biome domain Data type Figure Brief description Map showing the 52 drainage sub-basins within the Amazon biome domain. The name of each basin was based on the main tributaries: 1 = Gurupi, 2 = Capim Guama, 3 = Belem, 4 = Tocantis, 5 = Vila Nova, 6 = Araguari, 7 = Oyapok, 8 = Maroni, 9 = Suriname, 10 = Jaru, 11 = Xingu, 12 = Pari, 13 = Curua, 14 = Curua Uma, 15 = Tapajos, 16 = Trombetas, 17 = Courentyne, 18 = Berbice, 19 = Essequibo, 20 = Amacuru Aruta, 21 = Maués Açu, 22 = Amazonas, 23 = Uatumã, 24 = Negro (low), 25 = Unini, 26 = Jauoperi, 27 = Rio Branco, 28 = Negro Demini, 29 = Aracã, 30 = Negro (tall), 31 = Negro (medium) , 32 = Uapés, 33 = Caroni, 34 = Caura, 35 = Orinoco Ventuani, 36 = Orinoco (medium), 37 = Guaviare, 38 = Madeira (low), 39 = Roosevelt, 40 = Madeira (medium), 41 = Jiparaná , 42 = Madeira (tall), 43 = Itenez O Guaporé, 44 = Mamoré, 45 = Mamoré the Great, 46 = Beni, 47 = Purus, 48 = Jurua, 49 = Jupará Coquetá, 50 = Patumaio, 51 = Ucayali, 52 = Maranon. File: oo_1030472.docx https://binary.pensoft.net/file/1030472 Marcos Penhacek, Thadeu Sobral de Souza, Jessie Pereira dos Santos, Vinicius Guerra, Domingos de Jesus Rodrigues

0AAA31B0-4BE3-5E2F-8F1E-D4C001B12864 10.3897/BDJ.12.e109785.suppl2 Supplementary material 2 Scientific collections Data type Table Brief description Scientific collections that have records of amphibians stored on online database platforms and that made up the database of the present study. File: oo_1030473.docx https://binary.pensoft.net/file/1030473 Marcos Penhacek, Thadeu Sobral de Souza, Jessie Pereira dos Santos, Vinicius Guerra & Domingos de Jesus Rodrigues

64F9FBDF-D2FB-5CDF-B9CA-E687329E6E93 10.3897/BDJ.12.e109785.suppl3 Supplementary material 3 Main inventories Data type Table Brief description Main inventories on amphibian diversity in the Amazon used in this database. File: oo_1030474.docx https://binary.pensoft.net/file/1030474 Marcos Penhacek, Thadeu Sobral de Souza, Jessie Pereira dos Santos, Vinicius Guerra & Domingos de Jesus Rodrigues

73DC6C32-CCF4-5305-9003-9DFE56D1C6F1 10.3897/BDJ.12.e109785.suppl4 Supplementary material 4 Disregarded species Data type Text Brief description Species disregarded due to inconsistencies in their known distributions. File: oo_1036624.docx https://binary.pensoft.net/file/1036624 Marcos Penhacek, Thadeu Sobral de Souza, Jessie Pereira dos Santos, Vinicius Guerra & Domingos de Jesus Rodrigues

DA760FF0-1C2A-55E5-9E57-55E6327455D7 10.3897/BDJ.12.e109785.suppl5 Supplementary material 5 Number of amphibian records (presence only) for the Amazon Data type Table Brief description Number of amphibian records (presence only) for the Amazon obtained from searches of different data sources and after each filtering phase, as well as percentage of valid records, number of sample sites and richness. File: oo_1036626.docx https://binary.pensoft.net/file/1036626 Marcos Penhacek, Thadeu Sobral de Souza, Jessie Pereira dos Santos, Vinicius Guerra & Domingos de Jesus Rodrigues

E6DC6FFD-2C0F-59C0-9E91-6B17DE59C1FC 10.3897/BDJ.12.e109785.suppl6 Supplementary material 6 Amphibian species diversity in the Amazon Data type Table Brief description Amphibian species diversity in the Amazon. Conservation status: Least concern (LC), Not Applicable (NA), Data Deficient (DD), Near Threatened (NT), Vulnerable (VU), Endangered (EN), Critically Endangered (CR). Distribution biome: Atlantic Forest (AF), Amazon (AM), Andes (AN), Caatinga (CA), Cerrado (CE), Chaco (CH), Caribbean (CB), North of South America (NSA**) Northwest of South America (NW*), Paramo (PA), Subparamo (SPA), Pacifico (PC), Pampas (PM), Pantanal (PN). * species distributed in different biomes in the northwest of South and Central America, reaching as far as the southern United States, ** species distributed in the north of South America, reaching islands in Central America such as Trinidad and Tobago. Countries: BO = Bolívia, BR =Brasil, CO = Colômbia, EC = Equador, GY = Guiana, FG = French Guiana, PE = Peru, SR = Suriname and VE = Venezuela. File: oo_1036627.docx https://binary.pensoft.net/file/1036627 Marcos Penhacek, Thadeu Sobral de Souza, Jessie Pereira dos Santos, Vinicius Guerra & Domingos de Jesus Rodrigues

40A54747-62AA-5E3D-A91F-92ACEA6C8EF3 10.3897/BDJ.12.e109785.suppl7 Supplementary material 7 Number of amphibians from the Amazon domain Data type Figure Brief description Number of amphibians from the Amazon domain. Sample sites (A) and records (B). File: oo_1036628.docx https://binary.pensoft.net/file/1036628 Marcos Penhacek, Thadeu Sobral de Souza, Jessie Pereira dos Santos, Vinicius Guerra & Domingos de Jesus Rodrigues

E6E6C7D8-F1F4-5A21-B0AA-FA2C0501314E 10.3897/BDJ.12.e109785.suppl8 Supplementary material 8 Richness and endemism of amphibian species Data type Table Brief description Richness and endemism of amphibian species, area size (km²), main rivers and country(ies) of each of the 52 drainage sub-basins considered in this study for the Amazon domain. File: oo_1036630.docx https://binary.pensoft.net/file/1036630 Marcos Penhacek, Thadeu Sobral de Souza, Jessie Pereira dos Santos, Vinicius Guerra & Domingos de Jesus Rodrigues

0E3065E4-425A-5DD2-BADF-B85E78126F72 10.3897/BDJ.12.e109785.suppl9 Supplementary material 9 Relationship between the total richness and number of endemic species Data type Figure Brief description Relationship between the total richness and number of endemic species and the area (m²) of the 52 Amazon drainage sub-basins. File: oo_1036632.docx https://binary.pensoft.net/file/1036632 Marcos Penhacek, Thadeu Sobral de Souza, Jessie Pereira dos Santos, Vinicius Guerra & Domingos de Jesus Rodrigues

7EA41EE5-D443-582F-AFB9-A42903972F33 10.3897/BDJ.12.e109785.suppl10 Supplementary material 10 Sum of occurrences for richness Data type Figure Brief description Sum of occurrences for richness, distribution and abundance of amphibians in the Amazon. Sources: GBIF=1, VertNet=2, SpeciesLink=3, SiBBr=4, SISBIO=5, Scientific Articles=6, Grey Literature=7 and Fieldwork=8. File: oo_1036633.docx https://binary.pensoft.net/file/1036633 Marcos Penhacek, Thadeu Sobral de Souza, Jessie Pereira dos Santos, Vinicius Guerra & Domingos de Jesus Rodrigues
