# Supplementary material for: Amazonian amphibians: diversity, spatial distribution patterns, conservation and sampling deficits
Source: Biodivers Data J. 2024 Oct 1;12:e109785. doi: 10.3897/BDJ.12.e109785 (PMC11471977; doi:10.3897/BDJ.12.e109785)
Supplement: Supplementary material 1 — Map showing the 52 drainage sub-basins within the Amazon biome domain [file bdj-12-e109785-s001.docx]

**Supplementary Material 1**

**Amazon amphibians: diversity, distribution patterns, conservation and sampling deficits**

Marcos Penhacek, Thadeu Sobral de Souza, Jessie Pereira dos Santos, Vinicius Guerra & Domingos de Jesus Rodrigues

**Figure S2.** Map showing the 52 drainage sub-basins within the Amazon biome domain. The name of each basin was based on the main tributaries: 1 = Gurupi, 2 = Capim Guama, 3 = Belem, 4 = Tocantis, 5 = Vila Nova, 6 = Araguari, 7 = Oyapok, 8 = Maroni, 9 = Suriname, 10 = Jaru, 11 = Xingu, 12 = Pari, 13 = Curua, 14 = Curua Uma, 15 = Tapajos, 16 = Trombetas, 17 = Courentyne, 18 = Berbice, 19 = Essequibo, 20 = Amacuru Aruta, 21 = Maués Açu, 22 = Amazonas, 23 = Uatumã, 24 = Negro (low), 25 = Unini, 26 = Jauoperi, 27 = Rio Branco, 28 = Negro Demini, 29 = Aracã, 30 = Negro (tall), 31 = Negro (medium) , 32 = Uapés, 33 = Caroni, 34 = Caura, 35 = Orinoco Ventuani, 36 = Orinoco (medium), 37 = Guaviare, 38 = Madeira (low), 39 = Roosevelt, 40 = Madeira (medium), 41 = Jiparaná , 42 = Madeira (tall), 43 = Itenez O Guaporé, 44 = Mamoré, 45 = Mamoré the Great, 46 = Beni, 47 = Purus, 48 = Jurua, 49 = Jupará Coquetá, 50 = Patumaio, 51 = Ucayali, 52 = Maranon. **
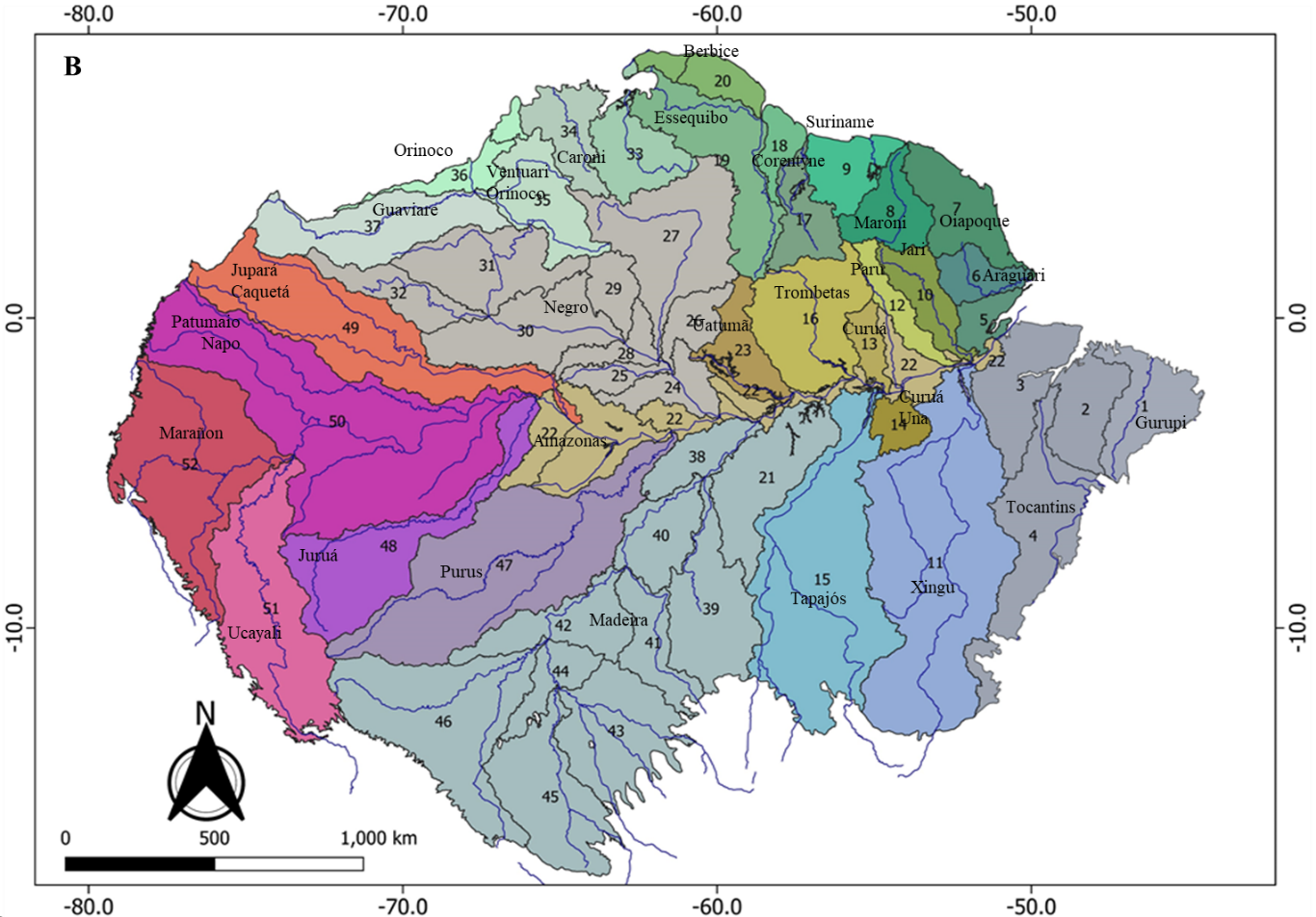
**
