# Supplementary material for: Amazonian amphibians: diversity, spatial distribution patterns, conservation and sampling deficits
Source: Biodivers Data J. 2024 Oct 1;12:e109785. doi: 10.3897/BDJ.12.e109785 (PMC11471977; doi:10.3897/BDJ.12.e109785)
Supplement: Supplementary material 2 — Scientific collections [file bdj-12-e109785-s002.docx]

**Supplementary Material 2**

**Amazon amphibians: diversity, distribution patterns, conservation and sampling deficits**

Marcos Penhacek, Thadeu Sobral de Souza, Jessie Pereira dos Santos, Vinicius Guerra & Domingos de Jesus Rodrigues

**Table S1.** Scientific collections that have records of amphibians stored on online database platforms and that made up the database of the present study.

| **Institution** | **Code** | **Country** |
| --- | --- | --- |
| American Museum of Natural History | AMNH | USA |
| Auburn University Museum of Natural History | AUM | USA |
| Banco de Vida/ CJ Museum/ Amphibian collection | CJ | Ecuador |
| Brigham Young University | BYU | USA |
| California Academy of Sciences | CAS | USA |
| Carnegie Museum of Natural History | CM | USA |
| Centro de Ornitología y Biodiversidad | CORBIDI | Peru |
| Coastal Plains Institute and Land Conservancy, USA | CPI | USA |
| Coleção Célio F. B. Haddad, Universidade Estadual Paulista | CFBH | Brazil |
| Coleção de Tecidos de Genética Animal, Sisbiota Tapajós, margem direita | CTGANSISTA D | Brazil |
| Coleção Tecidos Universidade Federal do Amazonas | CTGA-N | Brazil |
| Coleção Zoológica de Referência da Universidade Federal de Mato Grosso do Sul | ZUFMS-AMP | Brazil |
| Coleccion de Referência de Anfibios del Museo de Ciencias de la Universidad El Bosque | MCUB | Colombia |
| Collection of the Fauna of Amapá | IEPA | Brazil |
| Cornell University Museum of Vertebrates | CUMV | USA |
| División de Herpetología, Instituto Nacional de Biodiversidad | DHMECN | Ecuador |
| Fauna de Caño Cristales, Sierra de La Macarena, Meta - Colombia | CORMACARENA | Colombia |
| Florida Museum of Natural History | UF | USA |
| Fonoteca Neotropical Jacques Vielliard | FNJV | Brazil |
| Fundación Puerto Rastrojo - Colômbia | FPR-COLOMBIA | Colombia |
| Herpetological Collection of the Universidade Federal do Amapá | CECCAMPOS | Brazil |
| Herpetology Collection - Royal Ontario Museum | ROM | Canadá |
| Herpetology Collection NRM | NRM | Brazil |
| Instituto Chico Mendes de Conservação da Biodiversidade | ICMBio | Brazil |
| Instituto de Investigación de Recursos Biológicos Alexander von Humboldt Colombia - Colección de Anfibios | IAvH | Colombia |
| Instituto Nacional de Pesquisas da Amazônia - Herpetology Collection | INPA | Brazil |
| Laboratório de Zoologia de Altamira | LZATM | Brazil |
| Laboratório de Zoologia Adriano Giorgi at the Universidade Federal do Para | IZAG | Brazil |
| Louisiana State University Museum of Natural Science | LSUMZ | USA |
| Macaulay Library Audio and Video Collection | CLO | England |
| Monte L. Bean Life Science Museum, Brigham Young University, Provo, Utah, USA | BYU | USA |
| Museo Argentino de Ciencias Naturales "Bernardino Rivadavia" (MACN). Herpetology National Collection (MACNHe) | MACN | Argentina |
| Museo de Anfibios y Reptiles, Fundación Herpetológica Gustavo Orcés | FHGO | Ecuador |
| Museo de Biodiversidad del Peru | MUBI | Peru |
| Museo de Biología de la Universidad Central de Venezuela, Caracas | MHNLS | Venezuela |
| Museo de Historia Natural de la Universidad Nacional de San Agustin de Arequipa | MUSA | Peru |
| Museo de Historia Natural de la Universidad Nacional de San Antonio Abad del Cusco | MHNC-A | Peru |
| Museo de Historia Natural Gustavo Orcés del Instituto de Ciencias Biológicas de la Escuela Politécnica Nacional del Ecuador | MEPN | Ecuador |
| Museo de Historia Natural Universidad Nacional Mayor de San Marcos | MUSM | Peru |
| Museo de Zoológica, Universidad Tecnica Particular de Loja | MUTPL | Peru |
| Museo de Zoología de la Universidad del Azuay | MZUA.AN | Ecuador |
| Museo de Zoología de la Universidad Nacional de la Amazonía Peruana | MZUNAP | Peru |
| Museo de Zoología de la Universidad Tecnológica Indoamérica | MZUTI | Ecuador |
| Museo de Zoología of the Pontificia Universidad Católica del Ecuador | QCAZ | Ecuador |
| Museo de Zoología, Universidad San Francisco de Quito | ZSFQ | Peru |
| Museo de Zoología, Universidad Técnica Particular de Loja | MUTPL | Ecuador |
| Museo Nacional de Ciencias Naturales | MNCN | Espanha |
| Museo Noel Kempff Mercado, Santa Cruz de la Sierra (amphibians) | MNKA | Bolivia |
| Museu de Biodiversidade do Cerrado | AAG-UFU | Brazil |
| Museu de Biologia Prof. Mello Leitão | MBML | Brazil |
| Museu de Ciências e Tecnologia, Pontifícia Universidade Católica do Rio Grande do Sul, Brazil | MCP | Brazil |
| Museu de História Natural da Universidade Federal de Alagoas | MUFAL | Brazil |
| Museu de Zoologia da Universidade Estadual de Campinas | ZUEC | Brazil |
| Museu de Zoologia João Moojen of the Universidade Federal de Viçosa | MZUFV | Brazil |
| Museu de Zoologia Universidade São Paulo | MZUSP | Brazil |
| Museu Nacional do Rio de Janeiro | MNRJ | Brazil |
| Museu Paraense Emilio Goeldi | MPEG | Brazil |
| Muséum d'histoire naturelle de la Ville de Genève | MHNG | Suisse |
| Museum National d?Histoire Naturelle (Paris, France) The reptiles and amphibians collection (RA) | MNHN | France |
| Museum of Comparative Zoology, Harvard University | MCZ | USA |
| Museum of Natural History University of Colorado | UCM | USA |
| Museum of Vertebrate Zoology | MVZ | USA |
| Museum of Zoology Senckenberg Dresden | MTD | Germany |
| Museum of Zoology, University of Michigan | UMMZ | USA |
| National Museum of Natural History, Smithsonian Institution | USNM | USA |
| National Museum Prague, Czech Republic | NMP-P6V | Czech Republic |
| Natural History Museum (London) Collection Specimens | NHMUK | England |
| Natural History Museum in Lima | UNMSM | Peru |
| Natural History Museum of Los Angeles County | LACM | USA |
| Natural History Museum, London | NHMLondon | England |
| New Brunswick Museum | NBMB | Canadá |
| Pontificia Universidad Javeriana - Coleccion de anfibios del Museo de Historia Natural | PUJ | Colombia |
| Pontifícia Universidade Católica do Rio Grande do Sul - Amphibian Collection of Museu de Ciências e Tecnologia | PUCRS | Brazil |
| Queensland Museum | QM | Australia |
| Royal Belgian Institute of Natural Sciences (Brussels, Belgium) | RBINS | Belgium |
| Royal Ontario Museum, Canada | ROM | Canadá |
| Secretaria de Estado do Meio Ambiente - Acre | SEMA-Acre | Brazil |
| Senckenberg Forschungsinstitut und Naturmuseum, Frankfurt (Germany) | SMF | Germany |
| Smithsonian Institution, National Museum of Natural History | USNM | USA |
| South Australian Museum | SAMA | Australia |
| Staatliches Museum für Naturkunde Stuttgart | SMNS | Germany |
| Texas Natural History Collections | TNHC | USA |
| The Cornell Lab of Ornithology - Macaulay Library | CLO | USA |
| Universidad de Antioquia - Coleccion de anfibios - Museo de Herpetologia | MHUA-A | Colombia |
| Universidad de la Amazonia - Coleccion de Anfibios del Museo de Historia Natura | UAM | Colombia |
| Universidad de La Salle - Coleccion de Anfibios | MLS | Colombia |
| Universidad de los Andes | ANDES | Colombia |
| Universidad del Valle, Coleccion de anfibios y reptiles del Laboratorio de Herpetologia | UV-C | Colombia |
| Universidad Nacional de San Agustín | UNSA | Peru |
| Universidade de Brasilia | UNB | Brazil |
| Universidade Estadual de Campinas | UNICAMP | Brazil |
| Universidade Estadual Paulista - Instituto de Biociências | UNESP-IBILCE | Brazil |
| Universidade Federal de Goiás | ZUFG | Brazil |
| Universidade Federal de Mato Grosso (Cuiabá) Coleção zoologica herpetologia | UFMT | Brazil |
| Universidade Federal de Mato Grosso do Sul - Coleção Zoológica de Referência/Amphibia | UFMS | Brazil |
| Universidade Federal de Mato Grosso (Sinop) Acervo Biologico da Amazônia Meridional | ABAM-H | Brazil |
| Universidade Federal de Rondônia | UNIR | Brazil |
| Universidade Federal do Acre | UFAC | Brazil |
| Universidade Federal do Acre, Rio Branco | UFAC-RB | Brazil |
| Universidade Federal do Amapá | UFAP | Brazil |
| Universidade Federal do Amazonas, Paulo Bürhnheim Zoological Collection | UFAM | Brazil |
| Universidade Federal do Para/ Laboratório Zoologia setor Amphibia | UFPA-LZA | Brazil |
| Universidade Federal Rural de Pernambuco/ Coleção herpetologica | CHP-UFRPE | Brazil |
| University of Florida Herpetology | UF | USA |
| University of Kansas - Herpetology Collection (KUBI) | KUBI | USA |
| Vertebrate Zoology Division - Herpetology, Yale Peabody Museum | YPM | USA |
| Zoologisches Forschungsmuseum Alexander Koenig | ZFMK | Germany |
