# Supplementary material for: Amazonian amphibians: diversity, spatial distribution patterns, conservation and sampling deficits
Source: Biodivers Data J. 2024 Oct 1;12:e109785. doi: 10.3897/BDJ.12.e109785 (PMC11471977; doi:10.3897/BDJ.12.e109785)
Supplement: Supplementary material 3 — Main inventories [file bdj-12-e109785-s003.docx]

**Supplementary Material 3**

**Amazon amphibians: diversity, distribution patterns, conservation and sampling deficits**

Marcos Penhacek, Thadeu Sobral de Souza, Jessie Pereira dos Santos, Vinicius Guerra & Domingos de Jesus Rodrigues

**Table S2.** Main inventories on amphibian diversity in the Amazon used in this database.

| Fonte | Localidade | Estado/Pais | Espécies | Táxons | Incerteza % | Referencia |
| --- | --- | --- | --- | --- | --- | --- |
| Artigo Check List - 01 | Reserva Biológica do Parazinho | Amapá/Brazil | 12 | 13 | 8 | Araújo and Costa-Campos 2014 |
| Artigo Check List - 02 | Floresta Nacional do Pau Rosa | Amazonas, Brazil | 31 | 37 | 16 | Ferreira et al. 2017 |
| Artigo Check List - 03 | Boca do Acre e Apauní | Amazonas, Brazil | 59 | 59 | 0 | França and Venâncio 2010 |
| Artigo Check List - 04 | Espigão do Oeste, Fazenda Jaburi | Rondônia/Brazil | 41 | 47 | 13 | Bernarde et al. 2007 |
| Artigo Check List - 05 | Floresta do Rio Moa | Acre/Brazil | 47 | 50 | 6 | Bernarde et al. 2013 |
| Artigo Check List - 06 | Resex Riozinho da Liberdade | Acre/Brazil | 77 | 83 | 7 | Bernarde et al. 2011 |
| Artigo Check List - 07 | Gurupi Biological Reserve | Maranhão/Brazil | 30 | 31 | 3 | Freitas et al. 2017 |
| Artigo Check List - 08 | Northern Peruvian dry forest | Cajamarca/Peru | 13 | 14 | 7 | Koch et al. 2018 |
| Artigo Check List - 09 | Urucu Petrol Basin | Amazonas, Brazil | 45 | 54 | 17 | da Costa Prudente et al. 2013 |
| Artigo Check List - 10 | Santa Cruz Forest Reserve | Loreto, Peru | 106 | 106 | 0 | Metcalf et al. 2020 |
| Artigo Check List - 11 | Cancão Municipal Natural Park | Amapá/Brazil | 47 | 49 | 4 | e Silva and Costa-Campos 2018 |
| Artigo Check List - 12 | Reserva Extrativista do Rio Cajari | Amapá/Brazil | 34 | 43 | 21 | Queiroz et al. 2011 |
| Artigo Check List - 13 | RESEX Chico Mendes | Acre/Brazil | 31 | 31 | 0 | França et al. 2017 |
| Artigo Check List - 14 | Reserva Extrativista Rio Gregório | Amapá/Brazil | 41 | 46 | 11 | Pantoja and de Fraga 2012 |
| Artigo Check List - 15 | Area Urbana de Altamira | Pará/Brazil | 11 | 15 | 27 | Knispel andBarros 2009 |
| Artigo Check List - 16 | Reserva Biológica do Tapirapé | Pará/Brazil | 34 | 35 | 3 | Bernardo et al. 2012 |
| Artigo Check List - 17 | Amapá National Forest | Amapá/Brazil | 44 | 53 | 17 | Benicio and Lima 2017 |
| Artigo Check List - 18 | P N Montanhas do Tumucumaque | Amapá/Brazil | 56 | 70 | 20 | Lima 2008 |
| Artigo Check List - 19 | Porto Walter | Acre/Brazil | 58 | 58 | 0 | da Fonseca et al. 2019 |
| Artigo Check List - 20 | Estação Ecológica Rio Acre | Acre/Brazil | 59 | 63 | 6 | Freitas et al. 2020 |
| Artigo Check List - 21 | Zona Urbana de Manaus | Amazonas, Brazil | 18 | 18 | 0 | Silva et al. 2011 |
| Artigo Check List - 22 | Baixo Rio Purus | Amazonas, Brazil | 63 | 75 | 16 | Waldez et al. 2013 |
| Artigo Check List - 23 | Bosque Municipal de Ouro Preto do Oeste | Rondônia/Brazil | 14 | 14 | 0 | Araujo et al. 2013 |
| Artigo Check List - 24 | PCH São João da Barra | Mato Grosso | 18 | 30 | 40 | Ávila, Ricardo and Kawashita-Ribeiro 2011 |
| Artigo Check List - 25 | Floresta Nacional do Trairão | Pará/Brazil | 31 | 35 | 11 | Mendes-Pinto and Souza 2011 |
| Artigo Check List - 26 | Reserva Extrativista Beija-Flor Brilho do Fogo | Amapá/Brazil | 23 | 25 | 8 | Santos et al. 2011 |
| Artigo Check List - 27 | Estação Ecológica Alto Maués | Amazonas, Brazil | 12 | 19 | 37 | Ferreira 2021 |
| Artigo Check List - 28 | Região da Guyana | Guyana | 66 | 69 | 4 | Cole et al. 2013 |
| Artigo Check List - 29 | Médio Rio Purus | Amazonas, Brazil | 55 | 59 | 7 | Ramalho et al. 2016 |
| Artigo Check List - 30 | Sipaliwini Nature Reserve | Suriname | 65 | 76 | 14 | Fouquet et al. 2015 |
| Artigo Check List - 31 | Caverna Planaltina | Pará/Brazil | 27 | 30 | 10 | Oliveira et al. 2013 |
| Artigo Check List - 32 | São Jorge I Farm | Acre/Brazil | 33 | 38 | 13 | Miranda et al. 2014 |
| Artigo Check List - 33 | Itacoatiara | Amazonas, Brazil | 27 | 29 | 7 | Menin et al. 2019 |
| Artigo Check List - 34 | Santa Isabel do Rio Negro | Amazonas, Brazil | 32 | 41 | 22 | Menin et al. 2017 |
| Artigo Check List - 35 | Serra da Mocidade | Roraima/Brazil | 18 | 23 | 22 | Leandro J.C.L. Moraes et al. 2017 |
| Artigo Check List - 36 | La Nube Biological Station | Puno Region | 15 | 18 | 17 | Llanqui et al. 2019 |
| Artigo Check List - 37 | Rio Preto da Eva | Amazonas, Brazil | 23 | 23 | 0 | Ilha and Dixo 2010 |
| Artigo Check List - 38 | Yurimaguas | Loreto, Peru | 11 | 11 | 0 | Philipp Böning et al. 2017 |
| Artigo Check List - 38 | Pacaya-Samiria | Loreto, Peru | 20 | 20 | 0 | Philipp Böning et al. 2017 |
| Artigo Check List - 38 | Nauta | Loreto, Peru | 19 | 19 | 0 | Philipp Böning et al. 2017 |
| Artigo Check List - 38 | Iquitos | Loreto, Peru | 18 | 18 | 0 | Philipp Böning et al. 2017 |
| Artigo Check List - 38 | Pevas | Loreto, Peru | 9 | 9 | 0 | Philipp Böning et al. 2017 |
| Artigo Check List - 38 | Janauari | Amazonas, Brazil | 15 | 15 | 0 | Philipp Böning et al. 2017 |
| Artigo Check List - 38 | Catalão | Amazonas, Brazil | 19 | 19 | 0 | Philipp Böning et al. 2017 |
| Artigo Check List - 39 | Jarú | Rondônia/Brazil | 18 | 19 | 5 | Piatti et al. 2012 |
| Artigo Check List - 40 | Madre de Dios | Perú | 106 | 114 | 7 | Von May et al 2009 |
| Artigo Check List - 41 | Região das Guianas Norte Pará | Pará/Brazil | 71 | 80 | 11 | Ávila-Pires et al. 2010 |
| Artigo Check List - 42 | Área de Proteção Ambiental do Rio Curiaú | Amapá/Brazil | 26 | 28 | 7 | Lima et al. 2017 |
| Artigo Check List - 43 | floating meadows along the Amazon river | Rondônia/Brazil | 14 | 21 | 33 | Boening et al. 2017 |
| Dissertação - 01 | RESEX do Cazumbá-Iracema | Acre/Brazil | 48 | 54 | 11 | Diógenes and Rocha 2019 |
| Dissertação - 02 | Rio Branco/Porto Acre | Acre/Brazil | 36 | 37 | 3 | de Paula 2018 |
| EIA - 01 | UHE Castanhal | Mato Grosso/Brazil | 32 | 43 | 26 | Nova Terra 2015 |
| EIA - 02 | UHE Colider | Mato Grosso/Brazil | 33 | 33 | 0 | JGP 2009 |
| Livro - 01 | Ferrovia Carajas | Maranhão/Pará/ Brazil | 61 | 71 | 14 | Rubem et al. 2020 |
| Livro - 02 | Reserva Extrativista Marinha Caeté-Taperaçu | Pará/Brazil | 13 | 14 | 7 | Fernandes and Silva 2016 |
| Livro - 03 | Reserva Adolpho Ducke | Amazonas, Brazil | 48 | 50 | 4 | Lima et al. 2012 |
| Livro - 04 | Perú: Ampiyacu, Apayacu, Yaguas, Medio Putumayo | Iquitos/Perú | 54 | 64 | 16 | Rodríguez and Knell 2003 |
| Tese - 01 | Parque Nacional de Pacaás Novos | Rondônia/Brazil | 53 | 62 | 15 | Zaqueo 2017 |
| Relatório 1 | UHE Sinop | Mato Grosso/Brazil | 43 | 47 | 9 | UFMT 2019 |
| Relatório 2 | UHE Belo Monte | Pará/Brazil | 73 | 103 | 29 | Biota 2017 |

**References**

Acosta-Galvis, A. R., & Pinzón, A. (2018). Una nueva rana nodriza (Anura: Dendrobatidae) de los bosques de niebla asociados a la cuenca del Orinoco de Colombia. Biota Colombiana, 19(1), 160-190.

Almendáriz, A., Brito, J., Batallas, D., Vaca-Guerrero, J., & Ron, S. R. (2017). Una especie nueva de rana del género Chiasmocleis (Microhylidae: Gastrophryninae) de la Cordillera del Cóndor, Ecuador. Papéis Avulsos de Zoologia, 57(10), 119-136.

Antoine, F. O. U. Q. U. E. T., Vacher, J., Courtois, E. A., Villette, B., Reizine, H., Gaucher, P., ... & Kok, P. (2018). On the brink of extinction: two new species of Anomaloglossus from French Guiana and amended definitions of Anomaloglossus degranvillei and A. surinamensis (Anura: Aromobatidae). Zootaxa, 4379(1), 001-023.

Araújo R.M.G., Carvalho K.A., Meneguelli A. Z. and Zandonadi A.P. (2013). Levantamento de Anfibios do Bosque Municipal de Ouro Preto do Oeste, Rondônia Brasil. Saberes da UNIJIPA.

Araújo, A. S., & Costa-Campos, C. E. (2014). Anurans of the Reserva Biológica do Parazinho, municipality of Macapá, state of Amapá, eastern Amazon. Check List, 10(6), 1414-1419.

Ávila, R. W., & Kawashita-Ribeiro, R. A. (2011). Herpetofauna of São João da Barra Hydroelectric Plant, state of Mato Grosso, Brazil. Check list, 7(6), 750-755.

Ávila-Pires, T. C. S. D., Hoogmoed, M. S., & Rocha, W. A. D. (2010). Notes on the Vertebrates of northern Pará, Brazil: a forgotten part of the Guianan Region, I. Herpetofauna.

Benício, R. A., & Lima, J. D. (2017). Anurans of Amapá National Forest, Eastern Amazonia, Brazil. Herpetology Notes, 10, 627-633.

Bernarde, P. S., Albuquerque, S. D., Miranda, D. B. D., & Turci, L. C. B. (2013). Herpetofauna of the forest of Lower Moa River, Cruzeiro do Sul, Acre-Brazil. Biota Neotropica, 13(1), 220-244.

Bernarde, P.S. Ambientes e temporada de vocalização da anurofauna no Município de Espigão do Oeste, Rondônia, Sudoeste da Amazônia - Brasil (Amphibia: Anura). Biota Neotrop.  May/Aug 2007 vol. 7, no. 2

Bernarde, P.S., Machado, R.A. & Turci, L.C.B. Herpetofauna of Igarapé Esperança area in the Reserva Extrativista Riozinho da Liberdade, Acre, Brazil. Biota Neotrop. 11(3).

Bernardo, P. H., Guerra-Fuentes, R. A., Matiazzi, W., & Zaher, H. (2012). Checklist of amphibians and reptiles of Reserva Biológica do Tapirapé, Pará, Brazil. Check List, 8(5), 839-846.

Boening, P., Wolf, S., Upton, K., Menin, M., Venegas, P. J., & Loetters, S. (2017). Amphibian diversity and its turnover in floating meadows along the Amazon River. Salamandra, 53, 379-388.

Brito, J., & Almendáriz, A. (2018). Una especie nueva de rana Pristimantis (Amphibia: Strabomantidae) de ojos rojos de la Cordillera de Cóndor, Ecuador.

Brito, J., Batallas, D., & Yánez-Muñoz, M. H. (2017). Ranas terrestres Pristimantis (Anura: Craugastoridae) de los bosques montanos del río Upano, Ecuador: Lista anotada, patrones de diversidad y descripción de cuatro especies nuevas. Neotropical Biodiversity, 3(1), 125-156.

Brito-Zapata, D., & Reyes-Puig, C. (2021). A new species of terrestrial-breeding frog Pristimantis (Anura: Strabomantidae) from the Cordillera del Cóndor, Zamora Chinchipe, Ecuador. Neotropical Biodiversity, 7(1), 213-222.

Brown, J. L., Siu-Ting, K., Von May, R., Twomey, E., Guillory, W. X., Deutsch, M. S., & Chavez, G. (2019). Systematics of the Ameerega rubriventris complex (Anura: Dendrobatidae) with descriptions of two new cryptic species from the East-Andean versant of Peru. Zootaxa, 4712(2), 211-235.

Caminer, M. A., Mila, B., Jansen, M., Fouquet, A., Venegas, P. J., Chavez, G., ... & Ron, S. R. (2017). Systematics of the Dendropsophus leucophyllatus species complex (Anura: Hylidae): Cryptic diversity and the description of two new species. PloS one, 12(3), e0171785.

Caminer, M. A., & Ron, S. R. (2020). Systematics of the Boana semilineata species group (Anura: Hylidae), with a description of two new species from Amazonian Ecuador. Zoological Journal of the Linnean Society, 190(1), 149-180.

Carrión-Olmedo, J. C., & Ron, S. R. (2021). A new cryptic species of the Pristimantis lacrimosus group (Anura, Strabomantidae) from the eastern slopes of the Ecuadorian Andes. Evolutionary Systematics, 5, 151.

Carvajal-Endara, S., Coloma, L. A., Morales-Mite, M. A., Guayasamin, J. M., Szekely, P., & Duellman, W. E. (2019). Phylogenetic systematics, ecology, and conservation of marsupial frogs (Anura: Hemiphractidae) from the Andes of southern Ecuador, with descriptions of four new biphasic species. Zootaxa, 4562(1), zootaxa-4562.

Carvalho, T. R. D., Simões, P. I., Gagliardi-Urrutia, G., Rojas-Runjaic, F. J., Haddad, C. F., & Castroviejo-Fisher, S. (2020). A New Forest-Dwelling Frog Species of the Genus Adenomera (Leptodactylidae) from Northwestern Brazilian Amazonia. Copeia, 108(4), 924-937.

Castillo-Urbina, E., Glaw, F., Aguilar-Puntriano, C., Vences, M., & Koehler, J. (2021). Genetic and morphological evidence reveal another new toad of the Rhinella festae species group (Anura: Bufonidae) from the Cordillera Azul in central Peru. Salamandra, 57, 181-195.

Castroviejo-Fisher, S., Peréz-Peña, P. E., Padial, J. M., & Guayasamin, J. M. (2012). A second species of the family Allophrynidae (Amphibia, Anura).(American Museum novitates, no. 3739).

Castroviejo-Fisher, S., Koehler, J., De La Riva, I., & Padial, J. M. (2017). A new morphologically cryptic species of Phyllomedusa (Anura: Phyllomedusidae) from Amazonian forests of northern Peru revealed by DNA sequences. Zootaxa, 4269(2), 245-264.

Catenazzi, A., Ttito, A., Diaz, M. I., & Shepack, A. (2017). Bryophryne phuyuhampatu sp. n., a new species of Cusco Andes frog from the cloud forest of the eastern slopes of the Peruvian Andes (Amphibia, Anura, Craugastoridae). ZooKeys, (685), 65.

Catenazzi, A., & Lehr, E. (2018). Pristimantis antisuyu sp. n. and Pristimantis erythroinguinis sp. n., two new species of terrestrial-breeding frogs (Anura, Strabomantidae) from the eastern slopes of the Andes in Manu National Park, Peru. Zootaxa, 4394(2), 185-206.

Catenazzi, A., & Ttito, A. (2018). Psychrophrynella glauca sp. n., a new species of terrestrial-breeding frogs (Amphibia, Anura, Strabomantidae) from the montane forests of the Amazonian Andes of Puno, Peru. PeerJ, 6, e4444.

Catenazzi, A., & Ttito, A. (2019). Noblella thiuni sp. n., a new (singleton) species of minute terrestrial-breeding frog (Amphibia, Anura, Strabomantidae) from the montane forest of the Amazonian Andes of Puno, Peru. PeerJ, 7, e6780.

Chasiluisa, V. D., Caminer, M. A., Varela-Jaramillo, A., & Ron, S. R. (2020). Description and phylogenetic relationships of a new species of treefrog of the Osteocephalus buckleyi species group (Anura: Hylidae). Neotropical Biodiversity, 6(1), 21-36.

Chávez G, Santa-Cruz R, Rodriguez D, Lehr E. 2015. Two new species of frogs of the genus Phrynopus (Anura: Terrarana: Craugastoridae) from the Peruvian Andes. Amphibian & Reptile Conservation 9(1) [Special Section]: 15?25 (e105).

Chávez, G., García-Ayachi, L. A., & Catenazzi, A. (2021). Beauty is in the eye of the beholder: Cruciform eye reveals new species of direct-developing frog (Strabomantidae, Pristimantis) in the Amazonian Andes. Evolutionary Systematics, 5, 81.

Cole, C. J., Townsend, C. R., Reynolds, R. P., MacCulloch, R. D., & Lathrop, A. (2013). Amphibians and reptiles of Guyana, South America: illustrated keys, annotated species accounts, and a biogeographic synopsis. Proceedings of the Biological Society of Washington, 125(4), 317-578.

Correia, L. L., Nunes, P. M. S., Gamble, T., Maciel, A. O., Marques-Souza, S., Fouquet, A., ... & Mott, T. (2018). A new species of Brasilotyphlus (Gymnophiona: Siphonopidae) and a contribution to the knowledge of the relationship between Microcaecilia and Brasilotyphlus. Zootaxa, 4527(2), 186-196.

Costa-Campos, C. E., Gama, S. L., Galeno, É. O., Silva, D. W., Corrêa, K. J. G., Almeida, D. P., & Santiago, A. G. (2014). New record and distribution map of Hypsiboas lanciformis (Cope, 1871) (Amphibia, Anura, Hylidae) in Eastern Amazonia, Brazil. Check List, 10(4), 960-961.

Cusi, J. C., Moravec, J., Lehr, E., & Gvo?dík, V. (2017). A new species of semiarboreal toad of the Rhinella festae group (Anura, Bufonidae) from the Cordillera Azul National Park, Peru. ZooKeys, (673), 21.

da Costa Prudente, A. L., Maschio, G., Sturaro, M., Travassos, A., & Santos-Costa, M. C. (2013). Anurans of the Urucu Petrol Basin, municipality of Coari, state of Amazonas, northern Brazil. Check List, 9, 601.

da Fonseca, W. L., da Silva, J. D., Abegg, A. D., da Rosa, C. M., & Bernarde, P. S. (2019). Herpetofauna of Porto Walter and surrounding areas, southwest Amazonia, Brazil. Herpetology Notes, 12, 91-107.

da Rocha, M. I. L. E. N. E., Ribeiro, C., & CS, Raniere Garcez (2016). Richness and abundance of anurans (amphibia) in pasture and secondary forest areas near to Porto Velho (Rondonia, Brazil). *Revista Colombiana de Ciencia Animal-RECIA*, *8*(1), 7-13.

da Silva Fróis, R. D. P., de Carvalho, J. C., & Ruz, E. J. H. (2018). Variation in vegetation cover affect the herpetofauna assembly composition at the Serra Azul, eastern Amazon. Revista de Biologia Neotropical/Journal of Neotropical Biology, 15(1), 9-21.

De Andrade, F. S., Da Silva, L. A., Koroiva, R., Fadel, R. M., & Santana, D. J. (2019). A new species of Pseudopaludicola Miranda-Ribeiro, 1926 (Anura: Leptodactylidae: Leiuperinae) from an Amazonia-Cerrado transitional zone, state of Tocantins, Brazil. Journal of Herpetology, 53(1), 68-80.

De Carvalho, T. R., Angulo, A., Barrera, D. A., Aguilar-Puntriano, C., & Haddad, C. F. (2020). Hiding in Plain Sight: A Fourth New Cryptic Species of the Adenomera andreae Clade (Anura: Leptodactylidae) from Southwestern Amazonia. Herpetologica, 76(3), 304-314.

De Carvalho, T. R., Angulo, A., Kokubum, M. N., Barrera, D. A., De Souza, M. B., Haddad, C. F., & Giaretta, A. A. (2019). A new cryptic species of the Adenomera andreae clade from southwestern Amazonia (Anura, Leptodactylidae). Herpetologica, 75(3), 233-246.

De Carvalho, T. R., Giaretta, A. A., Angulo, A., Haddad, C. F., & Peloso, P. L. (2019). A new Amazonian species of Adenomera (Anura: Leptodactylidae) from the Brazilian state of Pará: a tody-tyrant voice in a frog. American Museum Novitates, 2019(3919), 1-21.

de Freitas, M. A., Venâncio, N. M., Abegg, A. D., dos Santos Azevedo, W., de Oliveira Pereira, V., Zanotti, A. P., ... & Moura, G. J. B. (2020). Herpetofauna at the Rio Acre Ecological Station, Amazon Rainforest, Brazil. Herpetology Notes, 13, 33-48.

de Freitas, M. A., Vieira, R. S., Entiauspe-Neto, O. M., e Sousa, S. O., Farias, T., Sousa, A. G., & de Moura, G. J. B. (2017). Herpetofauna of the Northwest Amazon forest in the state of Maranhão, Brazil, with remarks on the Gurupi Biological Reserve. ZooKeys, (643), 141.

de Jesus Rodrigues, D., de Morais Lima, M., & Kawashita-Ribeiro, R. A. (2016). Amphibia, Anura, Hylidae, Cruziohyla craspedopus (Funkhouser, 1957): distribution extension, new state record and distribution map in Brazil. Check List, 7(2), 149-150.

De la Riva, I., Chaparro, J. C., Castroviejo-Fisher, S., & Padial, J. M. (2018). Underestimated anuran radiations in the high Andes: five new species and a new genus of Holoadeninae, and their phylogenetic relationships (Anura: Craugastoridae). Zoological Journal of the Linnean Society, 182(1), 129-172.

de Paula, Y. A. P. (2018). Padrões de diversidade e fenologia de anfíbios em fragmentos de floresta tropical no Sudoeste da Amazônia, Brasil. Dissertação mestrado Universidade Federal do Acre, 37p.

de Souza Queiroz, S., da Silva, A. R., dos Reis, F. M., Lima, J. D., & Lima, J. R. F. (2011). Anfíbios de uma área de castanhal da Reserva Extrativista do Rio Cajari, Amapá. Biota Amazônia (Biote Amazonie, Biota Amazonia, Amazonian Biota), 1(1), 1-18.

de Oliveira, E. A., da Silva, L. A., Silva, E. A. P., Guimarães, K. L. A., Penhacek, M., Martínez, J. G., ... & Hernández-Ruz, E. J. (2020). Four new species of Pristimantis Jiménez de la Espada, 1870 (Anura: Craugastoridae) in the eastern Amazon. PloS one, 15(3), e0229971.

Diógenes, Luã Carlos Rocha (2019). Riqueza, abundância e composição de anfíbios em dois ambientes nos arredores de uma comunidade extrativista no sudoeste da Amazônia: Dissertação mestrado Universidade Federal do Acre 40 f.

Duellman, W. E. (2019). The last one: A new species of Osteocephalus (Anura: Hylidae) from Colombia, with comments on the morphological and behavioral diversity within the genus. Phyllomedusa: Journal of Herpetology, 18(2), 141-157.

EIA UHE Castanheira Vol 2.pdf

e Silva, Y. B. S., & Costa-Campos, C. E. (2016). Hyalinobatrachium iaspidiense (Ayarzaguena, 1992) (Anura: Centrolenidae): first record in Amapá state, Brazil and geographic distribution map. Check List, 12(2), 1849.

e Silva, Y. B. S., & Costa-Campos, C. E. (2018). Anuran species composition of Cancão Municipal Natural Park, Municipality of Serra do Navio, Amapá state, Brazil. ZooKeys, (762), 131.

Fernandes, M. E. B., & Silva, R. P. D. (2016). Anfíbios e répteis. Os Manguezais da costa norte brasileira.

Ferrão M, Moravec J, Fraga R, Almeida AP, Kaefer IL, Lima AP (2017) A new species of Scinax from the Purus-Madeira interfluve, Brazilian Amazonia (Anura, Hylidae). ZooKeys 706: 137?162.

Ferrão, M., de Fraga, R., Moravec, J., Kaefer, I. L., & Lima, A. P. (2018). A new species of Amazonian snouted treefrog (Hylidae: Scinax) with description of a novel species-habitat association for an aquatic breeding frog. PeerJ, 6, e4321.

Ferrão, M., Lima, A. P., Ron, S., Santos, S. P. D., & Hanken, J. (2020). New Species of Leaf-litter Toad of the Rhinella margaritifera Species Group (Anura: Bufonidae) from Amazonia. Copeia, 108(4), 967-986.

Ferrão, M., Moravec, J., Hanken, J., & Lima, A. P. (2020). A new species of Dendropsophus (Anura, Hylidae) from southwestern Amazonia with a green bilobate vocal sac. ZooKeys, 942, 77.

Ferrão, M., Moravec, J., Kaefer, I. L., de Fraga, R., & Lima, A. P. (2018). New species of Scinax (Anura: Hylidae) with red-striped eyes from Brazilian Amazonia. Journal of Herpetology, 52(4), 472-488.

Ferreira et al. Amphibians and reptiles from Floresta Nacional de Pau-Rosa, Amazonas, Brazil: an important protected area at the heart of the Amazon

Ferreira, A. S. Rapid survey of the herpetofauna of Estação Ecológica Alto Maués: a rarely accessed area in the Brazilian Amazonia.

Fouquet, A., Jairam, R., Ouboter, P., & Kok, P. J. (2020). Two new species of Anomaloglossus (Anura: Aromobatidae) of the stepheni group from Suriname. Zootaxa, 4820(1), 147-164.

Fouquet, A., Jean-Pierre, V., Kadosoe, V., Ouboter, P., & Jairam, R. (2015). Checklist of the amphibians of the Sipaliwini area, Suriname. Herpetology Notes, 8, 63-68.

Fouquet, A., Leblanc, K., Fabre, A. C., Rodrigues, M. T., Menin, M., Courtois, E. A., ... & Kok, P. J. (2021). Comparative osteology of the fossorial frogs of the genus Synapturanus (Anura, Microhylidae) with the description of three new species from the Eastern Guiana Shield. Zoologischer Anzeiger, 293, 46-73.

Fouquet, A., Marinho, P., Réjaud, A., Carvalho, T. R., Caminer, M. A., Jansen, M., ... & Ron, S. (2021). Systematics and biogeography of the Boana albopunctata species group (Anura, Hylidae), with the description of two new species from Amazonia. Systematics and Biodiversity, 19(4), 375-399.

Fouquet, A., Vacher, J. P., Courtois, E. A., Deschamps, C., Ouboter, P., Jairam, R., ... & Kok, P. J. (2019). A new species of Anomaloglossus (Anura: Aromobatidae) of the stepheni group with the redescription of A. baeobatrachus (Boistel and de Massary, 1999), and an amended definition of A. leopardus Ouboter and Jairam, 2012. Zootaxa, 4576(3), zootaxa-4576.

França, D. P., Freitas, M. A. D., Ramalho, W. P., & Bernarde, P. S. (2017). Diversidade local e influência da sazonalidade sobre taxocenoses de anfíbios e répteis na Reserva Extrativista Chico Mendes, Acre, Brasil. Iheringia. Série Zoologia, 107.

França, F. G. R., & Venâncio, N. M. (2010). Reptiles and amphibians of a poorly known region in southwest Amazonia. Biotemas, 23(3), 71-84.

Guayasamin JM, Vieira J, Glor RE, Hutter CR. 2019. A new glassfrog (Centrolenidae: Hyalinobatrachium) from the Topo River Basin, Amazonian slopes of the Andes of Ecuador. Amphibian & Reptile Conservation 13(2) [General Section]: 133?144 (e194).

Guayasamin, J. M., Cisneros-Heredia, D. F., Maynard, R. J., Lynch, R. L., Culebras, J., & Hamilton, P. S. (2017). A marvelous new glassfrog (Centrolenidae, Hyalinobatrachium) from Amazonian Ecuador. ZooKeys, (673), 1.

Ilha, P., & Dixo, M. (2010). Anurans and Lizards, Rio Preto da Eva, Amazonas, Brazil. Check List, 6(1), 017-021.

Jansen, M., Santana, D. J., Teixeira, B. F. D. V., & Köhler, G. (2019). A new striped species of Dendropsophus (Anura: Hylidae) with a composite advertisement call and comments on the D. rubicundulus group. Vertebrate Zoology, 63(3), 227-246.

JGP 2009. Estudo de Impacto Ambiental, EIA UHE Colider Volume III

Jorge, R. F., Ferrão, M., & Lima, A. P. (2020). Out of Bound: A New Threatened Harlequin Toad (Bufonidae, Atelopus) from the Outer Borders of the Guiana Shield in Central Amazonia Described through Integrative Taxonomy. Diversity, 12(8), 310.

Kaefer, I. L., Rojas, R. R., Ferrão, M., Farias, I. P., & Lima, A. P. (2019). A new species of Amazophrynella (Anura: Bufonidae) with two distinct advertisement calls. Zootaxa, 4577(2), 316-334.

Knispel, S. R., & Barros, F. B. (2009). Anfíbios anuros da região urbana de Altamira (Amazônia Oriental), Pará, Brasil. Biotemas, 22(2), 191-194.

Koch, C., Venegas, P. J., Santa Cruz, R., & Boehme, W. (2018). Annotated checklist and key to the species of amphibians and reptiles inhabiting the northern Peruvian dry forest along the Andean valley of the Marañón River and its tributaries. Zootaxa, 4385(1), 1-101.

Kok, P. J., Nicolaï, M. P., Lathrop, A., & MacCulloch, R. D. (2018). Anomaloglossus meansi sp. n., a new Pantepui species of the Anomaloglossus beebei group (Anura, Aromobatidae). ZooKeys, (759), 99.

Lehr E, Moravec J (2017) A new species of Pristimantis (Amphibia, Anura, Craugastoridae) from a montane forest of the Pui Pui Protected Forest in central Peru (Región Junín). ZooKeys 645: 85?102.

Lehr E, von May R (2017) A new species of terrestrial-breeding frog (Amphibia, Craugastoridae, Pristimantis) from high elevations of the Pui Pui Protected Forest in central Peru. ZooKeys 660: 17?42.

Lehr, E. D. G. A. R., Von May, R., Moravec, J. I. Í., & Cusi, J. C. (2017). Three new species of Pristimantis (Amphibia, Anura, Craugastoridae) from upper montane forests and high Andean grasslands of the Pui Pui Protected Forest in central Peru. Zootaxa, 4299(3), 301-336.

Lehr, E., & von May, R. (2017). A new species of terrestrial-breeding frog (Amphibia, Craugastoridae, Pristimantis) from high elevations of the Pui Pui Protected Forest in central Peru. ZooKeys, (660), 17.

Lehr, E., Moravec, J., Cusi, J. C., & Gvo?dík, V. (2017). A new minute species of Pristimantis (Amphibia: Anura: Craugastoridae) with a large head from the Yanachaga-Chemillén National Park in central Peru, with comments on the phylogenetic diversity of Pristimantis occurring in the Cordillera Yanachaga. European Journal of Taxonomy, (325).

Lima, A. P., Guida, V. M. L., & Hödl, W. (2003). Agalychnis craspedopus: Geographic distribution. Herpetological Review, 34(4), 379-382.

Lima, J. D. (2008). A herpetofauna do Parque Nacional do Montanhas do Tumucumaque, Amapá, Brasil, Expedições I a IV. Inventários Biológicos Rápidos no Parque Nacional Montanhas do Tumucumaque, Amapá, Brasil. RAP Bulletin of Biological Assessment, 48, 38-50.

Lima, A. P., Magnusson, W. E., Menin, M., Erdtmann, L. K., Rodrigues, D. D. J., Keller, C., & Hödl, W. (2012). Guia de sapos da Reserva Adolpho Ducke-Amazônia Central.

Lima, J. R. F., Lima, J. D., Lima, S. D., Silva, R. B. L., & Andrade, G. V. D. (2017). Amphibians found in the Amazonian Savanna of the Rio Curiaú Environmental Protection Area in Amapá, Brazil. Biota Neotropica, 17(2).

Llanqui IB, Salas CY, Oblitas MP (2019) A preliminary checklist of amphibians and reptiles from the vicinity of La Nube Biological Station, Bahuaja-Sonene National Park, Peru. Check List 15 (5): 773?796.

Maciel A.O., Sampaio M.I.C., Hoogmoed M.S., Schneider H. 2018. Description of two new species of Rhinatrema (Amphibia: Gymnophiona) from Brazil and the return of Epicrionops niger to Rhinatrema. South American Journal of Herpetology 13:287?299.

Maciel, A. O., & Hoogmoed, M. S. (2018). A new species of Caecilia Linnaeus, 1758 (Amphibia: Gymnophiona: Caeciliidae) from French Guiana. Boletim do Museu Paraense Emílio Goeldi-Ciências Naturais, 13(1), 13-18.

Maciel, A. O., Mott, T., & Hoogmoed, M. S. (2009). A second species of Brasilotyphlus (Amphibia: Gymnophiona: Caeciliidae) from Brazilian Amazonia. Zootaxa, 2226(1), 19-27.

Maciel, A. O., Sampaio, M. I., Hoogmoed, M. S., & Schneider, H. (2018). Description of Two New Species of Rhinatrema (Amphibia: Gymnophiona) from Brazil and the Return of Epicrionops niger to Rhinatrema. South American Journal of Herpetology, 13(3), 287-299.

Mângia, S., Koroiva, R., & Santana, D. J. (2020). A new tiny toad species of Amazophrynella (Anura: Bufonidae) from east of the Guiana Shield in Amazonia, Brazil. PeerJ, 8, e9887.

Melo-Sampaio, P. R., De Oliveira, R. M., & Prates, I. (2018). A new nurse frog from Brazil (Aromobatidae: Allobates), with data on the distribution and phenotypic variation of western Amazonian species. South American Journal of Herpetology, 13(2), 131-149.

Melo-Sampaio, P. R., Ferrão, M., & de Lima Moraes, L. J. C. (2021). A NEW SPECIES OF OSTEOCEPHALUS STEINDACHNER, 1862 (ANURA, HYLIDAE), FROM BRAZILIAN AMAZONIA. Breviora, 572(1), 1-21.

Mendes-Pinto, T. J., & Souza, S. M. D. (2011). Preliminary assessment of amphibians and reptiles from Floresta Nacional do Trairão, with a new snake record for the Pará state, Brazilian Amazon. Salamandra, 47(4), 199-206.

Meneghelli, D., Messias, M. R., & Sampaio, P. R. M. (2011). Amphibia, Anura, Hylidae, Cruziohyla craspedopus (Funkhouser, 1957): distribution extension in southwestern Amazonia, state of Rondônia, Brazil. Check List, 7(6), 811-812.

Menin, M., de Carvalho, V. T., Almeida, A. P., Gordo, M., Oliveira, D. P., Luiz, L. F., ... & Hrbek, T. (2017). Amphibians from Santa Isabel do Rio Negro, Brazilian Amazonia. Phyllomedusa: Journal of Herpetology, 16(2), 183-199.

Menin, M., Ferreira, R. F. B., Melo, I. B., Gordo, M., Hattori, G. Y., & santanna, B. S. (2019). Anuran diversity in urban and rural zones of the Itacoatiara municipality, central Amazonia, Brazil. Acta Amazonica, 49(2), 122-130.

Metcalf, M., Marsh, A., Torres, E., Graham, D., & Gunnels, C. (2020). Herpetofauna of the Santa Cruz Forest Preserve in the Peruvian Amazon Basin. Herpetology Notes, 13, 753-767.

Moraes, L. J. C. L., Pavan, D., & Lima, A. P. (2019). A new nurse frog of Allobates masniger-nidicola complex (Anura, Aromobatidae) from the east bank of Tapajós River, eastern Amazonia. Zootaxa, 4648(3), 401-434.

Moraes, L. J., & Lima, A. P. (2021). A New Nurse Frog (Allobates, Aromobatidae) with a Cricket-Like Advertisement Call from Eastern Amazonia. Herpetologica, 77(2), 146-163.

Moraes, L. J., de Almeida, A. P., de Fraga, R., Rojas, R. R., Pirani, R. M., Silva, A. A., ... & Werneck, F. P. (2017). Integrative overview of the herpetofauna from Serra da Mocidade, a granitic mountain range in northern Brazil. ZooKeys, (715), 103.

Neves, M. O., da Silva, L. A., Akieda, P. S., Cabrera, R., Koroiva, R., & Santana, D. J. (2017). A new species of poison frog, genus Ameerega (Anura: Dendrobatidae), from the southern Amazonian rain forest. Salamandra, 53, 485-493.

Oliveira EA de, Rodrigues LR, Kaefer IL, Pinto KC, Hernández-Ruz EJ (2017) A new species of Pristimantis from eastern Brazilian Amazonia (Anura, Craugastoridae) ZooKeys 687: 101?129.

Oliveira, E. A., & Hernández-Ruz, E. J. (2017). New species of glassfrog, genus Hyalinobatrachium (Anura: Centrolenidae), for the Brazilian Amazon revealed by mitochondrial DNA and morphology. Int. J. Res. Stud. Biosci, 5(3), 41-52.

Oliveira, E. A., Ruz, E. H., & Barros, F. B. (2013). Herpetofauna de las proximidades de la Caverna Planaltina, Brasil Novo, Pará (Amazonia brasileña). Herpetotropicos, 9(1-2), 55-68.

Ortega-Andrade, H. M., Deichmann, J. L., & Chaparro, J. C. (2021). Two New Cryptic Pristimantis (Anura, Craugastoridae) from the Southern Amazon Basin of Peru with Taxonomic Comments on Pristimantis imitatrix (Duellman, 1978). South American Journal of Herpetology, 21(1), 41-64.

Páez NB, Ron SR (2019) Systematics of Huicundomantis, a new subgenus of Pristimantis (Anura, Strabomantidae) with extraordinary cryptic diversity and eleven new species. ZooKeys 868: 1?112.

Pantoja, D., & de Fraga, R. (2012). Herpetofauna of the Reserva Extrativista do Rio Gregório, Juruá Basin, southwest Amazonia, Brazil. Check List, 8, 360.

Peloso, P. L., De Oliveira, R. M., Sturaro, M. J., Rodrigues, M. T., Lima-Filho, G. R., Bitar, Y. O., ... & Aleixo, A. (2018). Phylogeny of map tree frogs, Boana semilineata species group, with a new Amazonian species (Anura: Hylidae). South American Journal of Herpetology, 13(2), 150-169.

Piatti, L., Sanches, V., Amaro, P., Araújo, J., & Bernarde, P. (2012). Anurans of a disturbed area in Jarú, Rondônia, Brazil. Check List, 8, 83.

Pitman, N., Smith, R. C., Vriesendorp, C., Moskovits, D. K., Piana, R., Knell, G., & Wachter, T. (Eds.). (2004). Perú: Ampiyacu, Apayacu, Yaguas, Medio Putumayo. Field Museum, Environmental and Conservation Programs.

Ramalho, W. P., Andrade, M. S., Matos, L. R. A. D., & Vieira, L. J. S. (2016). Amphibians of varzea environments and floating meadows of the oxbow lakes of the Middle Purus River, Amazonas, Brazil. Biota Neotropica, 16(1).

Reyes-Puig, C., Reyes-Puig, J. P., Velarde-Garcéz, D. A., Dávalos, N., Mancero, E., Navarrete, M. J., ... & Ron, S. R. (2019). A new species of terrestrial frog Pristimantis (Strabomantidae) from the upper basin of the Pastaza River, Ecuador. ZooKeys, 832, 113.

Reyes-Puig, J. P., Reyes-Puig, C., Ron, S., Ortega, J. A., Guayasamin, J. M., Goodrum, M., ... & Yánez-Muñoz, M. H. (2019). A new species of terrestrial frog of the genus Noblella Barbour, 1930 (Amphibia: Strabomantidae) from the Llanganates-Sangay Ecological Corridor, Tungurahua, Ecuador. PeerJ, 7, e7405.

Rivadeneira CD, Venegas PJ, Ron SR (2018) Species limits within the widespread Amazonian treefrog Dendropsophus parviceps with descriptions of two new species (Anura, Hylidae). ZooKeys 726: 25?77.

Rojas, R. R., Fouquet, A., Ron, S. R., Hernández-Ruz, E. J., Melo-Sampaio, P. R., Chaparro, J. C., ... & Hrbek, T. (2018). A Pan-Amazonian species delimitation: high species diversity within the genus Amazophrynella (Anura: Bufonidae). PeerJ, 6, e4941.

Rojas-Runjaic, F. J., Matta-Pereira, M. E., & Marca, E. (2018). Unveiling species diversity in collared frogs through morphological and bioacoustic evidence: a new Mannophryne (Amphibia, Aromobatidae) from Sierra de Aroa, northwestern Venezuela, and an amended definition and call description of M. herminae (Boettger, 1893). Zootaxa, 4461(4), 451-476.

RON, S. R. (2017). Nueva especie de rana bromelícola del género Pristimantis (Amphibia: Craugastoridae), meseta de la cordillera del Cóndor, Ecuador. Papéis Avulsos de Zoologia, 57(15), 177-195.

Ron, S. R., Caminer, M. A., Varela-Jaramillo, A., & Almeida-Reinoso, D. (2018). A new treefrog from Cordillera del Cóndor with comments on the biogeographic affinity between Cordillera del Cóndor and the Guianan Tepuis (Anura, Hylidae, Hyloscirtus). ZooKeys, (809), 97.

Ron, S. R., Carrión, J., Caminer, M. A., Sagredo, Y., Navarrete, M. J., Ortega, J. A., ... & Terán, C. (2020). Three new species of frogs of the genus Pristimantis (Anura, Strabomantidae) with a redefinition of the P. lacrimosus species group. ZooKeys, 993, 121.

Rubem A. P. Dornas, Samir G. Rolim 2020. Fauna de Vertebrados do Entorno da Estrada de Ferro Carajás. Belo Horizonte. Rupestre 212p.

Sanchez-Nivicela, J. C., Urgiles, V. L., Navarrete, M. J., Yanez-Munoz, M. H., & Ron, S. (2019). A bizarre new species of Lynchius (Amphibia, Anura, Strabomantidae) from the Andes of Ecuador and first report of Lynchius parkeri in Ecuador. Zootaxa, 4567(1), 001-024.

Santa-Cruz, R., von May, R., Catenazzi, A., Whitcher, C., López Tejeda, E., & Rabosky, D. L. (2019). A new species of terrestrial-breeding frog (Amphibia, Strabomantidae, Noblella) from the upper Madre de Dios watershed, Amazonian Andes and lowlands of southern Peru. Diversity, 11(9), 145.

Santana, D. J., da Silva, L. A., Sant?Anna, A. C., Shepard, D. B., & Mângia, S. (2021). A new species of Proceratophrys Miranda-Ribeiro, 1920 (Anura, Odontophrynidae) from Southern Amazonia, Brazil. PeerJ, 9, e12012.

Santos, F. P., Sanches, P. R., & Costa-Campos, C. E. (2019). Anurans and reptiles of the Reserva Extrativista Beija-Flor Brilho de Fogo, Amapa? state, eastern Amazon. Herpetology Notes, 12, 799-807.

Serrano-Rojas, S. J., Whitworth, A., Villacampa, J., Von May, R., Gutierrez, R. C., Padial, J. M., & Chaparro, J. C. (2017). A new species of poison-dart frog (Anura: Dendrobatidae) from Manu province, Amazon region of southeastern Peru, with notes on its natural history, bioacoustics, phylogenetics, and recommended conservation status. Zootaxa, 4221(1), 71-94.

Silva, E. P., Mendes-Pinto, T. J., Júnior, L. H. C., & Sales, M. E. P. (2011). Riqueza de espécies de anfíbios anuros em um fragmento florestal na área urbana de Manaus, Amazonas, Brasil. Revista de Biologia e Farmácia, 5(2), 131-144.

Simoes, P. I., Costa, J. C. L., Rojas-Runjaic, F. J., Gagliardi-Urrutia, G., Sturaro, M. J., Peloso, P. L., & Castroviejo-Fisher, S. (2018). A new species of Phyzelaphryne Heyer, 1977 (Anura: Eleutherodactylidae) from the Japurá River basin, with a discussion of the diversity and distribution of the genus. Zootaxa, 4532(2), 203-230.

Simoes, P. I., Gagliardi-Urrutia, L. A. G., Rojas-Runjaic, F. J., & Castroviejo-Fisher, S. (2018). A new species of nurse-frog (Aromobatidae, Allobates) from the Juami River basin, northwestern Brazilian Amazonia. Zootaxa, 4387(1), 109-133.

Souza JRD, Ferrão M, Hanken J, Lima AP. 2020. A new nurse frog (Anura: Allobates) from Brazilian Amazonia with a remarkably fast multi-noted advertisement call. PeerJ 8:e9979

Székely P, Székely D, Ordóñez-Delgado L, Armijos-Ojeda D, Vörös J (2021) Our unknown neighbor: A new species of rain frog of the genus Pristimantis (Amphibia: Anura: Strabomantidae) from the city of Loja, southern Ecuador. PLoS ONE 16(10):

Székely, P., Eguiguren, J. S., Ordóñez-Delgado, L., Armijos-Ojeda, D., & Székely, D. (2020). Fifty years after: A taxonomic revision of the amphibian species from the Ecuadorian biodiversity hotspot Abra de Zamora, with description of two new Pristimantis species. PloS one, 15(9), e0238306.

Székely, P., Székely, D., Ordóñez-Delgado, L., Armijos-Ojeda, D., & Vörös, J. (2021). Our unknown neighbor: A new species of rain frog of the genus Pristimantis (Amphibia: Anura: Strabomantidae) from the city of Loja, southern Ecuador. PloS one, 16(10), e0258454.

Taucce, P. P., Costa-Campos, C. E., Haddad, C. F., & de Carvalho, T. R. (2020). A New Amazonian Species of the Diminutive Frog Genus Adelophryne (Anura: Brachycephaloidea: Eleutherodactylidae) from the State of Amapá, Northern Brazil. Copeia, 108(4), 746-757.

Ttito, A., & Catenazzi, A. (2021). Pristimantis achupalla sp. n., a new minute species of direct-developing frog (Amphibia, Anura, Strabomantidae) inhabiting bromeliads of the montane forest of the Amazonian Andes of Puno, Peru. PeerJ, 9, e11878.

Urgiles VL, Székely P, Székely D, Christodoulides N, Sanchez-Nivicela JC, Savage AE (2019) Genetic delimitation of Pristimantis orestes (Lynch, 1979) and P. saturninoi Brito et al., 2017 and description of two new terrestrial frogs from the Pristimantis orestes species group (Anura, Strabomantidae). ZooKeys 864: 111?146.

Urgiles, V. L., Posse, V., Timbe, B. A., Astudillo, P. X., & Sanchez-Nivicela, J. C. (2017). A new terrestrial frog (Anura: Craugastoridae) from the montane cloud forests of the southeastern Ecuadorian Andes. Zootaxa, 4318(3), 520-530.

Valencia, J. H., Duenas, M. R., Szekely, P., Batallas, D., Pulluquitin, F., & Ron, S. R. (2017). A new species of direct-developing frog of the genus Pristimantis (Anura: Terrarana: Craugastoridae) from Cordillera del Cóndor, Ecuador, with comments on threats to the anuran fauna of the region. Zootaxa, 4353(3), 447-466.

Valencia, J. H., Valladares-Suntasig, F., Tipantiza-Tuguminago, L., & Duenas, M. R. (2019). A new species of terrestrial-breeding frog of the genus Pristimantis (Anura: Terrarana: Craugastoridae) from the eastern Andean slopes of the southern Ecuador. Zootaxa, 4658(3), zootaxa-4658.

Velasquez, C. L. (2011). Influência de fatores ambientais sobre a distribuição e abundância de anuros na Amazônia Meridional.

Venâncio, N., Miranda, D., & de Albuquerque, S. (2014). Rapid survey of the herpetofauna in an area of forest management in eastern Acre, Brazil. Check List, 10, 893.

Venegas, P. J., Barboza, A. C., Riva, I., & Padial, J. M. (2018). A new species of Phrynopus from the northeastern Andes of Peru, its phylogenetic position, and notes on the relationships of Holoadeninae (Anura: Craugastoridae). Zootaxa, 4446(4), 501-524.

Venegas, P. J., García-Ayachi, L. A., & Catenazzi, A. (2022). Two New Species of Pristimantis (Anura: Strabomantidae) from Amazonas Department in Northeastern Peru. Taxonomy, 2(1), 20-40.

Venegas, P. J., García-Ayachi, L. A., Echevarría, L. Y., Paluh, D. J., Chávez?Arribasplata, J. C., Marchelie, A., & Catenazzi, A. (2021). A new species of marsupial frog (Anura, Gastrotheca) from the Cordillera de Colán in northeastern Peru. Vertebrate Zoology, 71, 201.

Von May, R., Siu-Ting, K., Jacobs, J. M., Medina-Mueller, M., Gagliardi, G., Rodriguez, L. O., & Donnelly, M. A. (2009). Species diversity and conservation status of amphibians in Madre de Dios, Southern Peru. Herpetological Conservation and Biology, 4(1), 14-29.

Waldez, F., Menin, M., & Vogt, R. C. (2013). Diversidade de anfíbios e répteis Squamata na região do baixo rio Purus, Amazônia Central, Brasil. Biota Neotropica, 13(1), 300-316.

Wilkinson, M., Reynolds, R. P., & Jacobs, J. F. (2021). A new genus and species of rhinatrematid caecilian (Amphibia: Gymnophiona: Rhinatrematidae) from Ecuador. Herpetological Journal, 31(1).

Yánez-Muñoz, M. H., Veintimilla-Yánez, D., Batallas, D., & Cisneros-Heredia, D. F. (2019). A new giant Pristimantis (Anura, Craugastoridae) from the paramos of the Podocarpus National Park, southern Ecuador. ZooKeys, 852, 137.

Zaqueo, K. D. (2017). Anurofauna do Parque Nacional de Pacaás Novos, Rondônia, Brasil.
