# Supplementary material for: Amazonian amphibians: diversity, spatial distribution patterns, conservation and sampling deficits
Source: Biodivers Data J. 2024 Oct 1;12:e109785. doi: 10.3897/BDJ.12.e109785 (PMC11471977; doi:10.3897/BDJ.12.e109785)
Supplement: Supplementary material 4 — Disregarded species [file bdj-12-e109785-s004.docx]

**Supplementary Material 4**

**Amazon amphibians: diversity, distribution patterns, conservation and sampling deficits**

Marcos Penhacek, Thadeu Sobral de Souza, Jessie Pereira dos Santos, Vinicius Guerra & Domingos de Jesus Rodrigues

**Text S3.** Species disregarded due to inconsistencies in their known distributions.

*Adenomera marmorata, Allobates rufalus, A. pumilio, A. spumaponensis, Agalychius callidrias, Ameerega bracata, Andinobates minutus, Atelopus cruciger, A. varius, Boana albomarginata, B. crepitans, B. faber, B. picturata, B. polytaenia, B. pulchella, Bokemanohyla hylax, Brachycephalus ephippidium, Bufo bufo, Bufates viridis, Caecilia leococephala, Caecilia thompsoni, Chiasmocleis bicegoi, C. leucosticta, Cochranella granulosa, Colosthetus inginatus, C. latinatus, Craugastor gollmeri, C. lineatus, C. rugosus, Dendropsophus ebracatus, D. micros, D. oliveirae, D. sarboni, D. semiculus, Elachistocleis ovalis, Eleutherodactylus inoptatus, E. martinicensis, E. warreni, E. petersi, Epipedobates boulengeri, Holoaden luederwaldti, Hoplobatrachus tigerinus, Hylodes aspers, Hylomantis granulosa, Incilius nebulifer, Leptodactylus labrosus, L. melanonotus, L. mystacinus, L. natalensis, L. savagei, Mannophryne trinitatis, Nannophryne variegata, Melanophryniscus moreirae, Plasmohyla cruzi, Phyllomedusa marginata, P. trinitatis, Physalaemus gracilis, P. maculiventris, Pithecopus azurea, Pristimantis cruentus, P. inoptatus, P. lymeni, Pseudacris cadaverina, Pseudopaludicola falcipes, Rhinella arenarum, R. crucefer, R. rubescensis, R. ornata, Scinax granulatus, S. hayli, S. squalirostris, S. staufferi, S. strigilatus, Sclerophrys camerunisis, Strabomantis bufoniformes, Odontophryne americanos, O. cultripes, Oephaga sylvatica, Oreobates heterodactylus, Telmatobius marmorata, Trachycephalus mesophaerus, Vitreorana eurignatha, V. uranoscopa.*
