# Supplementary material for: Amazonian amphibians: diversity, spatial distribution patterns, conservation and sampling deficits
Source: Biodivers Data J. 2024 Oct 1;12:e109785. doi: 10.3897/BDJ.12.e109785 (PMC11471977; doi:10.3897/BDJ.12.e109785)
Supplement: Supplementary material 5 — Number of amphibian records (presence only) for the Amazon [file bdj-12-e109785-s005.docx]

**Supplementary Material 5**

**Amazon amphibians: diversity, distribution patterns, conservation and sampling deficits**

Marcos Penhacek, Thadeu Sobral de Souza, Jessie Pereira dos Santos, Vinicius Guerra & Domingos de Jesus Rodrigues

**Table S4.** Number of amphibian records (presence only) for the Amazon obtained from searches of different data sources and after each filtering phase, as well as percentage of valid records, number of sample sites and richness

| **Source** | **Download** | **Taxonomic Filter** | **Geographic coordenate Filter** | **Biogeographic limits filter** | **Validated records** | **Sites** | **Richness** |
| --- | --- | --- | --- | --- | --- | --- | --- |
| GBIF | 324,582 | 283,032 | 160,632 | 53,259 | **16.4%** | **2,481** | **653** |
| SiBBr | 234,203 | 167,343 | 5,867 | 3,761 | **1.6%** | **93** | **139** |
| SpeciesLink | 180,625 | 127,132 | 86,202 | 37,293 | **20.6%** | **1,785** | **592** |
| VertNet | 68,570 | 67,535 | 36,202 | 29,931 | **5.5%** | **1,186** | **560** |
| SISBIO | 49,005 | 8,824 | 8,785 | 8,617 | **17.6%** | **1,798** | **254** |
| Gray literature | 39,045 | 22,730 | 22,730 | 22,724 | **58.2%** | **90** | **184** |
| Scientific papers | 3,496 | 3,496 | 3,496 | 3,397 | **97.2%** | **518** | **413** |
| Fieldwork | 3,460 | 2,568 | 2,437 | 1,696 | **49.1%** | **170** | **89** |
| **Total** | **902,986** | **682,660** | **326,351** | **160,643** | **17.8%** | **7,148** | **947** |
