# Supplementary material for: Amazonian amphibians: diversity, spatial distribution patterns, conservation and sampling deficits
Source: Biodivers Data J. 2024 Oct 1;12:e109785. doi: 10.3897/BDJ.12.e109785 (PMC11471977; doi:10.3897/BDJ.12.e109785)
Supplement: Supplementary material 6 — Amphibian species diversity in the Amazon [file bdj-12-e109785-s006.docx]

**Supplementary Material 7**

**Amazon amphibians: diversity, distribution patterns, conservation and sampling deficits**

Marcos Penhacek, Thadeu Sobral de Souza, Jessie Pereira dos Santos, Vinicius Guerra & Domingos de Jesus Rodrigues

**Table S5.** Amphibian species diversity in the Amazon. Conservation status: Least concern (LC), Not Applicable (NA), Data Deficient (DD), Near Threatened (NT), Vulnerable (VU), Endangered (EN), Critically Endangered (CR). Distribution biome: Atlantic Forest (AF), Amazon (AM), Andes (AN), Caatinga (CA), Cerrado (CE), Chaco (CH), Caribbean (CB), North of South America (NSA**) Northwest of South America (NW*), Paramo (PA), Subparamo (SPA), Pacifico (PC), Pampas (PM), Pantanal (PN). * species distributed in different biomes in the northwest of South and Central America, reaching as far as the southern United States, ** species distributed in the north of South America, reaching islands in Central America such as Trinidad and Tobago. Countries: BO = Bolívia, BR =Brasil, CO = Colômbia, EC = Equador, GY = Guiana, FG = Guiana Francesa, PE = Peru, SR = Suriname, and VE = Venezuela.

| **Species** | **Distribuition Biome** | **Distribuition country** | **Status IUCN** | **Occurrence sites** |
| --- | --- | --- | --- | --- |
| **Anura** |  |  |  |  |
| **Allophrynidae** |  |  |  |  |
| *Allophryne resplendens* | AM | PE | LC | 3 |
| *Allophryne ruthveni* | AM | BR, FG, GY, SR, VE | LC | 85 |
| **Aromobatidae** |  |  |  |  |
| *Allobates amissibilis* | AM | GY | VU | 1 |
| *Allobates brunneus* | AM, CE | BR, CO, EC, FG, GY, PE, SR, VE | LC | 39 |
| *Allobates caeruleodactylus* | AM | BR | LC | 5 |
| *Allobates conspicuus* | AM | PE | LC | 5 |
| *Allobates crombiei* | AM | BR | LC | 163 |
| *Allobates femoralis* | AM | BR, CO, EC, FG, GY, PE, SR | LC | 458 |
| *Allobates flaviventris* | AM | BR | LC | 2 |
| *Allobates fratisenescus* | AM | EC | VU | 1 |
| *Allobates fuscellus* | AM | BR | LC | 3 |
| *Allobates gasconi* | AM | BR | LC | 4 |
| *Allobates granti* | AM | FG, SR | LC | 5 |
| *Allobates grillicantus* | AM | BR | NA | 2 |
| *Allobates grillisimilis* | AM | BR | LC | 2 |
| *Allobates hodli* | AM | BR | LC | 3 |
| *Allobates insperatus* | AM | CO, EC | LC | 20 |
| *Allobates juami* | AM | BR | LC | 1 |
| *Allobates kingsburyi* | AM | CO, EC | LC | 12 |
| *Allobates magnussoni* | AM | BR | LC | 19 |
| *Allobates marchesianus* | AM | BO, BR, CO, EC, PE, SR, VE | LC | 98 |
| *Allobates masniger* | AM | BR | LC | 3 |
| *Allobates melanolaemus* | AM | PE | LC | 6 |
| *Allobates myersi* | AM | BR, CO | LC | 5 |
| *Allobates nidicola* | AM | BR | LC | 10 |
| *Allobates nunciatus* | AM | BR | LC | 7 |
| *Allobates ornatus* | AM | PE | DD | 3 |
| *Allobates paleovarzensis* | AM | BR, CO | LC | 7 |
| *Allobates peruvianus* | AM | BR | DD | 8 |
| *Allobates subfolionidificans* | AM | BR, PE | LC | 6 |
| *Allobates sumtuosus* | AM | BR, GY, SR | LC | 22 |
| *Allobates tapajos* | AM | BR | LC | 8 |
| *Allobates tinae* | AM | BR | LC | 5 |
| *Allobates trilineatus* | AM | BO, BR, CO, PE | LC | 41 |
| *Allobates vanzolinius* | AM | BR | LC | 1 |
| *Allobates velocicantus* | AM | BR | DD | 3 |
| *Allobates zaparo* | AM | EC, PE | LC | 35 |
| *Anomaloglossus apiau* | AM | BR, VE | NT | 8 |
| *Anomaloglossus baeobatrachus* | AM | BR, FG, SR | LC | 25 |
| *Anomaloglossus beebei* | AM | BR, FG, GY, SR | EN | 12 |
| *Anomaloglossus blanci* | AM | FG | EN | 20 |
| *Anomaloglossus degranvillei* | AM | FG, GY, SR | CR | 17 |
| *Anomaloglossus dewynteri* | AM | FG | CR | 2 |
| *Anomaloglossus kaiei* | AM | GY | EN | 8 |
| *Anomaloglossus leopardus* | AM | SR | LC | 1 |
| *Anomaloglossus meansi* | AM | GY | NA | 3 |
| *Anomaloglossus megacephalus* | AM | GY | DD | 7 |
| *Anomaloglossus mitaraka* | AM | SR | NA | 1 |
| *Anomaloglossus praderioi* | AM | GY | EN | 5 |
| *Anomaloglossus rufulus* | AM | VE | NT | 1 |
| *Anomaloglossus saramaka* | AM | SR | NA | 3 |
| *Anomaloglossus shrevei* | AM | VE | NT | 4 |
| *Anomaloglossus stepheni* | AM | BR, FG, PE, SR | LC | 29 |
| *Anomaloglossus surinamensis* | AM | SR | LC | 2 |
| *Anomaloglossus vacheri* | AM | SR | NA | 1 |
| *Hyloxalus pumilus* | AM | EC | CR | 1 |
| *Mannophryne molinai* | AM | BR | EN | 1 |
| *Rheobates palmatus* | AM, NR* | CO | LC | 4 |
| **Bufonidae** |  |  |  |  |
| *Amazophrynella bilinguis* | AM | BR | NA | 1 |
| *Amazophrynella bokermanni* | AM | BR | LC | 13 |
| *Amazophrynella gardai* | AM | BR | DD | 2 |
| *Amazophrynella manaos* | AM | BR | LC | 1 |
| *Amazophrynella matses* | AM | BR | LC | 3 |
| *Amazophrynella minuta* | AM | BR, CO, EC, FG, GY, PE | LC | 130 |
| *Amazophrynella teko* | AM | BR, CO, EC, FG, GY, PE, SR | LC | 48 |
| *Amazophrynella vote* | AM | BR | NA | 7 |
| *Atelopus barbotini* | AM | FG | NA | 6 |
| *Atelopus bomolochos* | AM | EC | CR | 5 |
| *Atelopus boulengeri* | AM | EC | CR | 3 |
| *Atelopus dimorphus* | AM | PE | DD | 1 |
| *Atelopus epikeisthos* | AM | PE | EN | 1 |
| *Atelopus flavescens* | AM | BR, FG | VU | 11 |
| *Atelopus franciscus* | AM | FG | LC | 1 |
| *Atelopus halihelos* | AM | EC | CR | 3 |
| *Atelopus hoogmoedi* | AM | BR, FG, GY, SR | NA | 32 |
| *Atelopus ignescens* | AM, AN | CO, EC | CR | 12 |
| *Atelopus manauensis* | AM | BR | NA | 4 |
| *Atelopus nepiozomus* | AM, PA | EC | EN | 6 |
| *Atelopus pachydermus* | AM | EC | CR | 4 |
| *Atelopus palmatus* | AM | EC | CR | 16 |
| *Atelopus pastuso* | AM | EC | CR | 1 |
| *Atelopus petersi* | AM | EC | CR | 7 |
| *Atelopus petriruizi* | AM | CO | CR | 1 |
| *Atelopus planispina* | AM | EC | CR | 4 |
| *Atelopus podocarpus* | AM | PE | CR | 1 |
| *Atelopus pulcher* | AM | BR, CO, EC, PE | VU | 23 |
| *Atelopus rugulosus* | AM | PE | CR | 1 |
| *Atelopus seminiferus* | AM | PE | EN | 1 |
| *Atelopus spumarius* | AM | BR, CO, EC, FG, GY, PE, SR | VU | 59 |
| *Atelopus tricolor* | AM | PE | CR | 6 |
| *Dendropsophus minimus* | AM | BR | NA | 1 |
| *Incilius coniferus* | AM, NR* | EC | LC | 2 |
| *Metaphryniscus sosai* | AM | VE | NT | 1 |
| *Nannophryne corynetes* | AM | PE | EN | 2 |
| *Oreophrynella cryptica* | AM | VE | NT | 1 |
| *Oreophrynella macconnelli* | AM | GY | VU | 9 |
| *Oreophrynella nigra* | AM | VE | VU | 2 |
| *Oreophrynella quelchii* | AM | BR, GY, VE | VU | 8 |
| *Oreophrynella vasquezi* | AM | VE | VU | 2 |
| *Osornophryne bufoniformis* | AM | EC | NT | 5 |
| *Osornophryne guacamayo* | AM | EC | EN | 4 |
| *Rhaebo blombergi* | AM, PC | EC, VE | NT | 4 |
| *Rhaebo ceratophrys* | AM | BR, CO, EC, PE | LC | 18 |
| *Rhaebo ecuadorensis* | AM | CO | NA | 1 |
| *Rhaebo glaberrimus* | AM | BR, CO, EC, PE | LC | 45 |
| *Rhaebo guttatus* | AM | BO, BR, CO, EC, FG, GY, PE, SR, VE | LC | 356 |
| *Rhaebo haematiticus* | AM, NR* | CO | LC | 2 |
| *Rhaebo nasicus* | AM | BR, GY | LC | 13 |
| *Rhinella acutirostris* | AM | BR | LC | 7 |
| *Rhinella alata* | AM, NR* | CO, EC, PE | DD | 16 |
| *Rhinella arborescandens* | AM | PE | EN | 1 |
| *Rhinella beebei* | AM, NA* | CO, VE | LC | 7 |
| *Rhinella castaneotica* | AM | BR, CO | LC | 365 |
| *Rhinella chullachaki* | AM | PE | NA | 1 |
| *Rhinella dapsilis* | AM | BR, CO, EC, PE | LC | 26 |
| *Rhinella diptycha* | AF, AM, CA, CE, CH, PM, PN | BO, BR | LC | 35 |
| *Rhinella exostosica* | AM | BR | NA | 4 |
| *Rhinella festae* | AM | EC, PE | LC | 20 |
| *Rhinella granulosa* | AF, AM, CA, CE, NA*, PM, PN | BO, BR, CO, EC, GY, PE, SR, VE | LC | 116 |
| *Rhinella humboldti* | AM, NA* | CO, VE | NA | 8 |
| *Rhinella inca* | AM | PE | LC | 5 |
| *Rhinella lescurei* | AM | BR | LC | 1 |
| *Rhinella lilyrodriguezae* | AM | PE | NA | 2 |
| *Rhinella magnussoni* | AM | BR | LC | 4 |
| *Rhinella major* | AM, CE, CH, PN | BO, BR, PE | NA | 68 |
| *Rhinella margaritifera* | AM, NR* | BO, BR, CO, EC, FG, GY, PE, SR, VE | LC | 737 |
| *Rhinella marina* | AM, IN* | BO, BR, CO, EC, FG, GY, PE, SR, VE | LC | 966 |
| *Rhinella merianae* | AM | BR, GY, SR, VE | NA | 27 |
| *Rhinella mirandaribeiroi* | AM, CE | BR | NA | 8 |
| *Rhinella ocellata* | AM, CE | BR | LC | 5 |
| *Rhinella poeppigii* | AM | BO, BR, PE | LC | 36 |
| *Rhinella proboscidea* | AM | BR, CO | LC | 46 |
| *Rhinella roqueana* | AM | CO, EC, PE | LC | 10 |
| *Rhinella rubropunctata* | AM, TF | BO | VU | 1 |
| *Rhinella spinulosa* | AM, AN, PC | BO, PE | LC | 15 |
| *Rhinella trifolium* | AM, AN | PE | NA | 1 |
| *Rhinella veraguensis* | AM | BO, PE | LC | 17 |
| **Centrolenidae** |  |  |  |  |
| *Centrolene azulae* | AM | PE | DD | 1 |
| *Centrolene buckleyi* | AM | CO, EC | VU | 12 |
| *Centrolene geckoidea* | AM | EC | NA | 1 |
| *Centrolene hybrida* | AM | CO | LC | 1 |
| *Centrolene lemniscata* | AM | PE | NA | 3 |
| *Centrolene medemi* | AM | CO, EC | EN | 3 |
| *Centrolene muelleri* | AM | PE | DD | 1 |
| *Centrolene pipilata* | AM | EC | CR | 10 |
| *Centrolene sanchezi* | AM | CO, EC | DD | 2 |
| *Centrolene solitaria* | AM | CO | EN | 1 |
| *Cochranella geijskesi* | AM | SR | LC | 2 |
| *Cochranella resplendens* | AM | CO, EC, PE | LC | 8 |
| *Espadarana audax* | AM | CO, EC, PE | LC | 20 |
| *Espadarana prosoblepon* | AM, NR* | CO | LC | 3 |
| *Hyalinobatrachium adespinosai* | AM | EC | NA | 1 |
| *Hyalinobatrachium bergeri* | AM | BO, PE | LC | 11 |
| *Hyalinobatrachium cappellei* | AM | BR, CO, GY, SR, VE | NA | 61 |
| *Hyalinobatrachium carlesvilai* | AM | BR, PE | NA | 5 |
| *Hyalinobatrachium iaspidiense* | AM | BR, FG, SR | DD | 12 |
| *Hyalinobatrachium kawense* | AM | SR | LC | 1 |
| *Hyalinobatrachium mondolfii* | AM | BR, PE, SR | LC | 5 |
| *Hyalinobatrachium muiraquitan* | AM | BR | NA | 1 |
| *Hyalinobatrachium munozorum* | AM | BR, CO, EC, PE | LC | 18 |
| *Hyalinobatrachium pellucidum* | AM | EC, PE | NT | 14 |
| *Hyalinobatrachium taylori* | AM | BR, FG, SR | LC | 9 |
| *Hyalinobatrachium yaku* | AM | EC | NA | 3 |
| *Nymphargus anomalus* | AM | EC | CR | 2 |
| *Nymphargus cariticommatus* | AM | EC | DD | 2 |
| *Nymphargus chancas* | AM | PE | DD | 1 |
| *Nymphargus cochranae* | AM | EC | VU | 9 |
| *Nymphargus garciae* | AM, AN | EC | VU | 3 |
| *Nymphargus laurae* | AM | EC | CR | 1 |
| *Nymphargus mariae* | AM | EC, PE | LC | 3 |
| *Nymphargus megacheirus* | AM | EC | EN | 9 |
| *Nymphargus nephelophila* | AM | CO | DD | 1 |
| *Nymphargus ocellatus* | AM | PE | DD | 1 |
| *Nymphargus oreonympha* | AM | CO | LC | 1 |
| *Nymphargus phenax* | AM | PE | EN | 2 |
| *Nymphargus pluvialis* | AM | PE | EN | 3 |
| *Nymphargus posadae* | AM | EC, PE | LC | 2 |
| *Nymphargus siren* | AM | EC | VU | 13 |
| *Nymphargus truebae* | AM | PE | CR | 7 |
| *Rulyrana flavopunctata* | AM | CO, EC | LC | 6 |
| *Rulyrana mcdiarmidi* | AM | PE | DD | 2 |
| *Rulyrana saxiscandens* | AM | PE | EN | 4 |
| *Rulyrana spiculata* | AM | EC, PE | NT | 10 |
| *Teratohyla adenocheira* | AM | BR | DD | 19 |
| *Teratohyla midas* | AM | BR, CO, EC, PE | LC | 35 |
| *Vitreorana gorzulae* | AM | GY | LC | 8 |
| *Vitreorana ritae* | AM | BR, CO, EC, FG, PE, SR, VE | DD | 48 |
| **Ceratophryidae** |  |  |  |  |
| *Ceratophrys calcarata* | AM, CB | CO, EC | LC | 2 |
| *Ceratophrys cornuta* | AM | BO, BR, CO, EC, FG, GY, PE, SR | LC | 129 |
| **Craugastoridae** |  |  |  |  |
| *Barycholos ternetzi* | AM, CE | BR | LC | 5 |
| *Bryophryne cophites* | AM, PA | PE | EN | 10 |
| *Bryophryne gymnotis* | AM | PE | LC | 1 |
| *Bryophryne hanssaueri* | AM | PE | LC | 4 |
| *Bryophryne phuyuhampatu* | AM | PE | LC | 1 |
| *Ceuthomantis smaragdinus* | AM | GY | VU | 3 |
| *Dischidodactylus duidensis* | AM | VE | NT | 1 |
| *Geobatrachus walkeri* | AM, CB | CO | EN | 1 |
| *Lynchius flavomaculatus* | AM | EC | DD | 7 |
| *Lynchius megacephalus* | AM | EC | NA | 1 |
| *Microkayla chapi* | AM | PE | NA | 1 |
| *Niceforonia brunnea* | AM | EC | EN | 2 |
| *Niceforonia dolops* | AM | CO, EC | VU | 11 |
| *Niceforonia elassodiscus* | AM | EC | NT | 8 |
| *Niceforonia fallaciosa* | AM | PE | DD | 2 |
| *Niceforonia lucida* | AM, AN | PE | EN | 2 |
| *Niceforonia nigrovittata* | AM | CO, EC, PE | LC | 47 |
| *Niceforonia peraccai* | AM | EC | DD | 10 |
| *Noblella lochites* | AM | EC | NT | 1 |
| *Noblella losamigos* | AM | PE | NA | 11 |
| *Noblella lynchi* | AM | PE | EN | 1 |
| *Noblella myrmecoides* | AM | BR, EC, PE | LC | 18 |
| *Noblella naturetrekii* | AM | EC | NA | 4 |
| *Noblella peruviana* | AM | BR, PE | DD | 5 |
| *Noblella thiuni* | AM | PE | NA | 1 |
| *Oreobates cruralis* | AM | BO, PE | LC | 8 |
| *Oreobates gemcare* | AM | PE | LC | 2 |
| *Oreobates granulosus* | AM | EC | LC | 2 |
| *Oreobates lehri* | AM | PE | EN | 3 |
| *Oreobates quixensis* | AM | BR, CO, EC, PE | LC | 178 |
| *Oreobates saxatilis* | AM | PE | LC | 10 |
| *Pristimantis aaptus* | AM | CO | LC | 6 |
| *Pristimantis academicus* | AM | BR, PE | NA | 7 |
| *Pristimantis acerus* | AM | EC | EN | 6 |
| *Pristimantis achatinus* | AM, CH, PA | CO, EC | LC | 2 |
| *Pristimantis achupalla* | AM | PE | NA | 1 |
| *Pristimantis acuminatus* | AM | BO, BR, CO, EC, GY, PE | LC | 44 |
| *Pristimantis albujai* | AM | EC | NA | 1 |
| *Pristimantis altamazonicus* | AM | BO, BR, CO, EC, PE | LC | 111 |
| *Pristimantis altamnis* | AM | CO, EC | LC | 15 |
| *Pristimantis amaguanae* | AM | EC | NA | 1 |
| *Pristimantis andinogigas* | AM | EC | NA | 1 |
| *Pristimantis aniptopalmatus* | AM | PE | LC | 1 |
| *Pristimantis antisuyu* | AM | PE | NA | 6 |
| *Pristimantis ardalonychus* | AM | PE | EN | 4 |
| *Pristimantis ashaninka* | AM | PE | LC | 1 |
| *Pristimantis atillo* | AM | EC | NA | 6 |
| *Pristimantis atratus* | AM | EC | EN | 7 |
| *Pristimantis attenboroughi* | AM | PE | NT | 4 |
| *Pristimantis aureolineatus* | AM | BR, EC | LC | 5 |
| *Pristimantis barrigai* | AM | EC | NA | 1 |
| *Pristimantis baryecuus* | AM | EC | EN | 11 |
| *Pristimantis bearsei* | AM | PE | DD | 3 |
| *Pristimantis bicantus* | AM | EC | NA | 1 |
| *Pristimantis bipunctatus* | AM | PE | LC | 2 |
| *Pristimantis bogotensis* | AM | CO | LC | 1 |
| *Pristimantis boucephalus* | AM | PE | DD | 1 |
| *Pristimantis brevicrus* | AM | EC | NA | 4 |
| *Pristimantis bromeliaceus* | AM | EC, PE | LC | 12 |
| *Pristimantis buccinator* | AM | PE | LC | 5 |
| *Pristimantis buckleyi* | AM | CO, EC | LC | 10 |
| *Pristimantis cajamarcensis* | AM, SP, PC | EC | LC | 1 |
| *Pristimantis cajanuma* | AM | EC | NA | 7 |
| *Pristimantis calcaratus* | AM | CO | VU | 4 |
| *Pristimantis carvalhoi* | AM | BO, BR, CO, EC, PE | LC | 29 |
| *Pristimantis chiastonotus* | AM | BR, FG, SR | LC | 63 |
| *Pristimantis chloronotus* | AM, SP | EC | LC | 32 |
| *Pristimantis chomskyi* | AM | EC | NA | 1 |
| *Pristimantis churuwiai* | AM | EC | NA | 3 |
| *Pristimantis citriogaster* | AM | EC, PE | DD | 7 |
| *Pristimantis colodactylus* | AM | EC | LC | 5 |
| *Pristimantis condor* | AM | EC, PE | LC | 6 |
| *Pristimantis conspicillatus* | AM | BR, CO, EC, FG, PE, SR | LC | 118 |
| *Pristimantis corniger* | AM | CO | EN | 1 |
| *Pristimantis corrugatus* | AM | PE | LC | 1 |
| *Pristimantis cosnipatae* | AM | PE | CR | 8 |
| *Pristimantis cremnobates* | AM | EC | EN | 13 |
| *Pristimantis croceoinguinis* | AM | BR, CO, EC, PE | LC | 53 |
| *Pristimantis cruciocularis* | AM | PE | LC | 1 |
| *Pristimantis cryophilius* | AM, PA, SP | EC | EN | 3 |
| *Pristimantis cryptomelas* | AM, SP | EC | NT | 3 |
| *Pristimantis curtipes* | AM, AN | CO, EC | LC | 18 |
| *Pristimantis danae* | AM | PE | LC | 24 |
| *Pristimantis delius* | AM | BR, CO, EC, PE | DD | 10 |
| *Pristimantis dendrobatoides* | AM | GY | LC | 3 |
| *Pristimantis devillei* | AM, AN | EC | EN | 40 |
| *Pristimantis diadematus* | AM | BR, CO, EC, PE | LC | 58 |
| *Pristimantis divnae* | AM | PE | LC | 2 |
| *Pristimantis dorsopictus* | AM, PA | CO | VU | 1 |
| *Pristimantis epacrus* | AM | CO | LC | 1 |
| *Pristimantis eriphus* | AM | EC | VU | 3 |
| *Pristimantis ernesti* | AM | EC | VU | 1 |
| *Pristimantis eurydactylus* | AM | PE | LC | 2 |
| *Pristimantis exoristus* | AM | PE | DD | 2 |
| *Pristimantis fenestratus* | AM | BO, BR, EC, PE | LC | 514 |
| *Pristimantis festae* | AM, PA, SP | EC | EN | 16 |
| *Pristimantis frater* | AM, AN, OR | CO | LC | 1 |
| *Pristimantis gagliardi* | AM | PE | NA | 2 |
| *Pristimantis galdi* | AM | EC | LC | 13 |
| *Pristimantis ganonotus* | AM | EC | DD | 1 |
| *Pristimantis giorgii* | AM | BR | NA | 2 |
| *Pristimantis gladiator* | AM | EC | VU | 4 |
| *Pristimantis glandulosus* | AM, PA | EC | EN | 27 |
| *Pristimantis gloria* | AM | EC | NA | 1 |
| *Pristimantis gutturalis* | AM | BR, FG, SR | LC | 10 |
| *Pristimantis huicundo* | AM, PA | CO | DD | 1 |
| *Pristimantis humboldti* | AM | PE | DD | 1 |
| *Pristimantis ignicolor* | AM | EC | EN | 2 |
| *Pristimantis imitatrix* | AM | PE | LC | 5 |
| *Pristimantis incanus* | AM | EC | EN | 5 |
| *Pristimantis incertus* | AM, NA* | CO, VE | LC | 12 |
| *Pristimantis incomptus* | AM | EC, PE | LC | 14 |
| *Pristimantis infraguttatus* | AM | PE | DD | 2 |
| *Pristimantis inguinalis* | AM | BR, GY, SR | LC | 6 |
| *Pristimantis inusitatus* | AM | EC | VU | 2 |
| *Pristimantis jester* | AM | GY | LC | 5 |
| *Pristimantis katoptroides* | AM | EC | LC | 2 |
| *Pristimantis kichwarum* | AM | BR, CO, EC, PE | LC | 20 |
| *Pristimantis kirklandi* | AM | EC | DD | 1 |
| *Pristimantis kiruhampatu* | AM | PE | NA | 1 |
| *Pristimantis lacrimosus* | AM | BR, CO, EC, PE | LC | 61 |
| *Pristimantis lancinii* | AM, PA | VE | VU | 1 |
| *Pristimantis lanthanites* | AM | BR, CO, EC, FG, PE | LC | 91 |
| *Pristimantis latidiscus* | AM, NR* | EC | LC | 3 |
| *Pristimantis latro* | AM | BR, EC | NA | 8 |
| *Pristimantis ledzeppelin* | AM | EC | NA | 1 |
| *Pristimantis leoni* | AM, AN, PC | EC | LC | 11 |
| *Pristimantis leucopus* | AM | EC | EN | 12 |
| *Pristimantis librarius* | AM | CO, EC | DD | 11 |
| *Pristimantis limoncochensis* | AM | EC | NT | 4 |
| *Pristimantis lindae* | AM | PE | LC | 2 |
| *Pristimantis lirellus* | AM | PE | LC | 7 |
| *Pristimantis lividus* | AM | EC | EN | 7 |
| *Pristimantis lojanus* | AM | EC | NA | 7 |
| *Pristimantis luscombei* | AM | BR, CO, PE | DD | 5 |
| *Pristimantis lythrodes* | AM | CO | LC | 1 |
| *Pristimantis malkini* | AM | BR, CO, EC, PE | LC | 35 |
| *Pristimantis mallii* | AM | EC | NA | 3 |
| *Pristimantis marmoratus* | AM | BR, FG, GY, VE | LC | 31 |
| *Pristimantis martiae* | AM | BR, CO, EC, PE | LC | 29 |
| *Pristimantis matildae* | AM | EC | NA | 2 |
| *Pristimantis medemi* | AM | CO, PE, VE | LC | 19 |
| *Pristimantis melanogaster* | AM | PE | NT | 2 |
| *Pristimantis mendax* | AM | PE | LC | 10 |
| *Pristimantis metabates* | AM | PE | EN | 1 |
| *Pristimantis minutulus* | AM | PE | DD | 6 |
| *Pristimantis muranunka* | AM | EC | NA | 3 |
| *Pristimantis muscosus* | AM | PE | NT | 1 |
| *Pristimantis myersi* | AM, PA | CO | LC | 9 |
| *Pristimantis nangaritza* | AM | EC | NA | 2 |
| *Pristimantis nankints* | AM | EC | NA | 2 |
| *Pristimantis nelsongalloi* | AM | EC | NA | 2 |
| *Pristimantis nephophilus* | AM | PE | NT | 3 |
| *Pristimantis nigrogriseus* | AM | EC | VU | 8 |
| *Pristimantis nimbus* | AM | EC | NA | 2 |
| *Pristimantis ockendeni* | AM | BR, CO, EC, PE | LC | 143 |
| *Pristimantis okmoi* | AM | PE | NA | 2 |
| *Pristimantis olivaceus* | AM | PE | LC | 7 |
| *Pristimantis omeviridis* | AM | EC | LC | 1 |
| *Pristimantis orcus* | AM | PE | LC | 2 |
| *Pristimantis orestes* | AM, SP | EC | EN | 1 |
| *Pristimantis ornatus* | AM | PE | EN | 1 |
| *Pristimantis orphnolaimus* | AM | EC | LC | 4 |
| *Pristimantis padiali* | AM | PE | LC | 3 |
| *Pristimantis paisa* | AM | CO | LC | 1 |
| *Pristimantis pastazensis* | AM | EC | EN | 1 |
| *Pristimantis pataikos* | AM | PE | DD | 1 |
| *Pristimantis paulpittmani* | AM | PE | NA | 8 |
| *Pristimantis paululus* | AM | EC | LC | 8 |
| *Pristimantis percnopterus* | AM | PE | LC | 6 |
| *Pristimantis peruvianus* | AM | BR, CO, EC, PE | LC | 68 |
| *Pristimantis petersi* | AM, NR* | EC | NT | 10 |
| *Pristimantis petersioides* | AM | EC | NA | 17 |
| *Pristimantis pharangobates* | AM | PE | LC | 3 |
| *Pristimantis pictus* | AM | BR | NA | 6 |
| *Pristimantis platydactylus* | AM | BO, PE | LC | 5 |
| *Pristimantis pluvian* | AM | BR | NA | 2 |
| *Pristimantis prolatus* | AM | EC | EN | 13 |
| *Pristimantis proserpens* | AM | EC, PE | VU | 6 |
| *Pristimantis pseudoacuminatus* | AM | EC | LC | 16 |
| *Pristimantis pugnax* | AM | CO, EC | CR | 14 |
| *Pristimantis pulvinatus* | AM | GY, VE | LC | 11 |
| *Pristimantis pycnodermis* | AM | EC | EN | 13 |
| *Pristimantis quaquaversus* | AM | EC, PE | LC | 44 |
| *Pristimantis quintanai* | AM | EC | NA | 3 |
| *Pristimantis reichlei* | AM | BO, BR, PE | NA | 59 |
| *Pristimantis rhabdolaemus* | AM | PE | LC | 13 |
| *Pristimantis riveti* | AM | EC | NT | 7 |
| *Pristimantis romeroae* | AM | EC | NA | 1 |
| *Pristimantis rubicundus* | AM | EC | EN | 7 |
| *Pristimantis rufioculis* | AM | PE | VU | 1 |
| *Pristimantis sagittulus* | AM | PE | LC | 2 |
| *Pristimantis salaputium* | AM | PE | LC | 6 |
| *Pristimantis saltissimus* | AM | BR, GY | LC | 8 |
| *Pristimantis samaniegoi* | AM | EC | NA | 2 |
| *Pristimantis savagei* | AM | CO | NT | 2 |
| *Pristimantis schultei* | AM | PE | VU | 2 |
| *Pristimantis serendipitus* | AM | PE | EN | 1 |
| *Pristimantis simonsii* | AM | PE | VU | 1 |
| *Pristimantis sinschi* | AM | PE | NA | 2 |
| *Pristimantis sira* | AM | PE | NA | 2 |
| *Pristimantis skydmainos* | AM | BR, EC, PE | LC | 19 |
| *Pristimantis spinosus* | AM | EC | NT | 11 |
| *Pristimantis supernatis* | AM | CO, EC | VU | 17 |
| *Pristimantis tamsitti* | AM | CO | VU | 1 |
| *Pristimantis tantanti* | AM | PE | LC | 1 |
| *Pristimantis toftae* | AM | BO, PE | LC | 35 |
| *Pristimantis trachyblepharis* | AM | EC | LC | 18 |
| *Pristimantis unistrigatus* | AM, AN | CO, EC, PE | LC | 11 |
| *Pristimantis urichi* | AM, NA* | FG | LC | 1 |
| *Pristimantis variabilis* | AM | BR, CO, EC, PE | LC | 25 |
| *Pristimantis ventrimarmoratus* | AM | BO, CO, EC, PE | LC | 34 |
| *Pristimantis versicolor* | AM, AN | EC, PE | LC | 5 |
| *Pristimantis vertebralis* | AM | EC | VU | 1 |
| *Pristimantis vilarsi* | AM | BR, CO, VE | LC | 32 |
| *Pristimantis walkeri* | AM, AN, PC | EC | LC | 1 |
| *Pristimantis waoranii* | AM | EC | DD | 3 |
| *Pristimantis w-nigrum* | AM, PC | CO, EC | LC | 45 |
| *Pristimantis yantzaza* | AM | EC | NA | 16 |
| *Pristimantis zeuctotylus* | AM | BR, CO, FG, GY, SR, VE | LC | 42 |
| *Pristimantis zimmermanae* | AM | BR, CO | LC | 17 |
| *Psychrophrynella bagrecito* | AM | PE | CR | 2 |
| *Psychrophrynella glauca* | AM | PE | NA | 1 |
| *Psychrophrynella usurpator* | AM | PE | NT | 6 |
| *Psychrophrynella wettsteini* | AM | BO | VU | 1 |
| *Strabomantis cornutus* | AM | CO, EC, PE | VU | 18 |
| *Strabomantis sulcatus* | AM | BR, CO, EC, PE | LC | 56 |
| *Yunganastes fraudator* | AM, AN | BO | VU | 1 |
| *Yunganastes mercedesae* | AM | PE | LC | 1 |
| **Dendrobatidae** |  |  |  |  |
| *Adelphobates castaneoticus* | AM | BR | LC | 43 |
| *Adelphobates galactonotus* | AM | BR, CO | LC | 220 |
| *Adelphobates quinquevittatus* | AM | BR, CO, EC, FG, PE | LC | 95 |
| *Ameerega altamazonica* | AM | PE | NA | 1 |
| *Ameerega bassleri* | AM | PE | VU | 9 |
| *Ameerega bilinguis* | AM | CO, EC | LC | 11 |
| *Ameerega boliviana* | AM | BO | NT | 1 |
| *Ameerega cainarachi* | AM | PE | EN | 3 |
| *Ameerega flavopicta* | AM, CE | BR, VE | LC | 9 |
| *Ameerega hahneli* | AM | BR, CO, EC, FG, GY, PE, SR | LC | 197 |
| *Ameerega imasmari* | AM | PE | NA | 2 |
| *Ameerega ingeri* | AM | CO | DD | 4 |
| *Ameerega macero* | AM | BR, PE | LC | 12 |
| *Ameerega munduruku* | AM | BR | NA | 7 |
| *Ameerega panguana* | AM | PE | NA | 3 |
| *Ameerega parvula* | AM | CO, EC, PE | LC | 71 |
| *Ameerega petersi* | AM | BR, PE | LC | 10 |
| *Ameerega picta* | AM, CE, PN | BO, BR, CO, EC, GY, PE, SR, VE | LC | 104 |
| *Ameerega planipaleae* | AM | PE | CR | 1 |
| *Ameerega pulchripecta* | AM | BR | DD | 11 |
| *Ameerega shihuemoy* | AM | PE | EN | 9 |
| *Ameerega silverstonei* | AM | PE | EN | 3 |
| *Ameerega simulans* | AM | PE | LC | 3 |
| *Ameerega trivittata* | AM | BO, BR, CO, GY, PE, SR | LC | 279 |
| *Andinobates abditus* | AM | EC | CR | 3 |
| *Colostethus poecilonotus* | AM | PE | DD | 1 |
| *Dendrobates leucomelas* | AM | BR, GY, VE | LC | 21 |
| *Dendrobates tinctorius* | AM | BR, FG, SR | LC | 46 |
| *Excidobates mysteriosus* | AM | PE | EN | 3 |
| *Hyloxalus alessandroi* | AM | PE | NA | 1 |
| *Hyloxalus anthracinus* | AM | EC | CR | 7 |
| *Hyloxalus bocagei* | AM | CO, EC, PE | LC | 50 |
| *Hyloxalus cevallosi* | AM | EC | EN | 3 |
| *Hyloxalus craspedoceps* | AM | PE | DD | 1 |
| *Hyloxalus delatorreae* | AM, AN | EC | CR | 1 |
| *Hyloxalus elachyhistus* | AM, AN | PE | LC | 3 |
| *Hyloxalus eleutherodactylus* | AM | PE | DD | 1 |
| *Hyloxalus exasperatus* | AM, AN | EC | DD | 2 |
| *Hyloxalus faciopunctulatus* | AM | CO, PE | DD | 4 |
| *Hyloxalus fuliginosus* | AM | CO, EC, PE | DD | 28 |
| *Hyloxalus idiomelus* | AM | PE | DD | 7 |
| *Hyloxalus infraguttatus* | AM, AN | EC | NT | 2 |
| *Hyloxalus insulatus* | AM | PE | VU | 3 |
| *Hyloxalus leucophaeus* | AM | PE | DD | 1 |
| *Hyloxalus maculosus* | AM | EC | DD | 5 |
| *Hyloxalus marmoreoventris* | AM | EC | DD | 1 |
| *Hyloxalus mittermeieri* | AM | PE | DD | 2 |
| *Hyloxalus nexipus* | AM | EC, PE | LC | 23 |
| *Hyloxalus parcus* | AM | EC | DD | 2 |
| *Hyloxalus peculiaris* | AM | EC | DD | 1 |
| *Hyloxalus picachos* | AM | CO | NA | 1 |
| *Hyloxalus pulchellus* | AM, AN, PA | EC, SR | NT | 49 |
| *Hyloxalus pumilus* | AM | EC | DD | 1 |
| *Hyloxalus saltuarius* | AM | CO | DD | 1 |
| *Hyloxalus sauli* | AM | EC, PE | LC | 12 |
| *Hyloxalus shuar* | AM | EC | NT | 17 |
| *Hyloxalus sordidatus* | AM | PE | DD | 2 |
| *Hyloxalus subpunctatus* | AM, NA*, NR* | CO | LC | 1 |
| *Hyloxalus sylvaticus* | AM | PE | EN | 1 |
| *Hyloxalus vertebralis* | AM, AN | EC | CR | 6 |
| *Leucostethus argyrogaster* | AM | PE | LC | 1 |
| *Leucostethus fugax* | AM | EC, PE | DD | 5 |
| *Minyobates steyermarki* | AM | VE | CR | 1 |
| *Oophaga histrionica* | AM, PA | CO, EC | CR | 2 |
| *Ranitomeya amazonica* | AM | BR, CO, PE | DD | 16 |
| *Ranitomeya cyanovittata* | AM | BR | NA | 4 |
| *Ranitomeya defleri* | AM | BR, CO | LC | 4 |
| *Ranitomeya fantastica* | AM | PE | VU | 4 |
| *Ranitomeya imitator* | AM | PE | LC | 10 |
| *Ranitomeya reticulata* | AM | BR, PE | LC | 6 |
| *Ranitomeya sirensis* | AM | BR, PE | LC | 20 |
| *Ranitomeya toraro* | AM | BR | NA | 11 |
| *Ranitomeya uakarii* | AM | BR | LC | 1 |
| *Ranitomeya vanzolinii* | AM | BR, CO | LC | 4 |
| *Ranitomeya variabilis* | AM | BR, CO, EC, PE | DD | 13 |
| *Ranitomeya ventrimaculata* | AM | BR, CO, EC, FG, PE | LC | 37 |
| *Ranitomeya yavaricola* | AM | BR | DD | 1 |
| **Eleutherodactylidae** |  |  |  |  |
| *Adelophryne adiastola* | AM | BR, CO | LC | 3 |
| *Adelophryne amapaensis* | AM | BR | NA | 1 |
| *Adelophryne gutturosa* | AM | BR, GY | LC | 9 |
| *Adelophryne patamona* | AM | GY | DD | 5 |
| *Phyzelaphryne miriamae* | AM | BR | LC | 17 |
| *Phyzelaphryne nimio* | AM | BR | NA | 9 |
| **Hemiphractidae** |  |  |  |  |
| *Gastrotheca abdita* | AM | PE | DD | 1 |
| *Gastrotheca aguaruna* | AM | PE | NT | 6 |
| *Gastrotheca andaquiensis* | AM | CO, EC | LC | 12 |
| *Gastrotheca cuencana* | AM | EC | NA | 1 |
| *Gastrotheca dysprosita* | AM | PE | DD | 1 |
| *Gastrotheca espeletia* | AM | EC | EN | 10 |
| *Gastrotheca excubitor* | AM | PE | VU | 16 |
| *Gastrotheca gemma* | AM | PE | NA | 3 |
| *Gastrotheca griswoldi* | AM | PE | LC | 4 |
| *Gastrotheca guentheri* | AM, PC | EC | DD | 2 |
| *Gastrotheca longipes* | AM | EC, PE | LC | 20 |
| *Gastrotheca marsupiata* | AM | BO, PE | LC | 16 |
| *Gastrotheca monticola* | AM | PE | LC | 6 |
| *Gastrotheca nebulanastes* | AM | PE | EN | 4 |
| *Gastrotheca nicefori* | AM, NR* | CO | LC | 4 |
| *Gastrotheca ochoai* | AM | PE | EN | 3 |
| *Gastrotheca oresbios* | AM | PE | EN | 2 |
| *Gastrotheca orophylax* | AM | CO, EC | VU | 15 |
| *Gastrotheca ossilaginis* | AM | BR | DD | 1 |
| *Gastrotheca pacchamama* | AM | PE | EN | 1 |
| *Gastrotheca phalarosa* | AM | PE | DD | 1 |
| *Gastrotheca phelloderma* | AM | PE | VU | 2 |
| *Gastrotheca pseustes* | AM | EC | NT | 7 |
| *Gastrotheca riobambae* | AM | EC | EN | 5 |
| *Gastrotheca ruizi* | AM | CO | NT | 1 |
| *Gastrotheca spectabilis* | AM | PE | DD | 1 |
| *Gastrotheca splendens* | AM | BO | LC | 1 |
| *Gastrotheca stictopleura* | AM | PE | EN | 3 |
| *Gastrotheca testudinea* | AM | EC, PE | LC | 17 |
| *Gastrotheca turnerorum* | AM | EC | NA | 1 |
| *Gastrotheca weinlandii* | AM | CO, EC, PE | LC | 13 |
| *Hemiphractus bubalus* | AM | CO, EC, PE | NT | 22 |
| *Hemiphractus helioi* | AM | BR, PE | LC | 8 |
| *Hemiphractus johnsoni* | AM | EC, PE | EN | 11 |
| *Hemiphractus proboscideus* | AM | BR, CO, EC, PE | LC | 22 |
| *Hemiphractus scutatus* | AM | BR, EC, PE | LC | 30 |
| *Stefania ackawaio* | AM | GY | VU | 3 |
| *Stefania ayangannae* | AM | GY | VU | 7 |
| *Stefania coxi* | AM | GY | VU | 3 |
| *Stefania evansi* | AM | GY | DD | 25 |
| *Stefania ginesi* | AM | VE | NT | 3 |
| *Stefania goini* | AM | VE | NT | 1 |
| *Stefania marahuaquensis* | AM | VE | NT | 2 |
| *Stefania riae* | AM | VE | NT | 2 |
| *Stefania roraimae* | AM | GY | EN | 5 |
| *Stefania scalae* | AM | GY, VE | LC | 2 |
| *Stefania tamacuarina* | AM | BR | DD | 1 |
| *Stefania woodleyi* | AM | GY | DD | 14 |
| **Hylidae** |  |  |  |  |
| *Boana albopunctata* | AF, AM, CE | BO, BR, PE | LC | 55 |
| *Boana balzani* | AM | PE | LC | 3 |
| *Boana benitezi* | AM | BR, VE | DD | 13 |
| *Boana boans* | AM, NA*, NR* | BR, CO, EC, FG, GY, PE, SR, VE | LC | 476 |
| *Boana calcarata* | AM | BR, CO, EC, FG, GY, PE, SR | LC | 246 |
| *Boana callipleura* | AM | BO, PE | LC | 13 |
| *Boana cinerascens* | AM | BO, BR, CO, EC, FG, GY, PE, SR, VE | LC | 367 |
| *Boana courtoisae* | AM | BR, FG, SR | NA | 9 |
| *Boana dentei* | AM | BR, FG | LC | 17 |
| *Boana eucharis* | AM | BR | NA | 4 |
| *Boana fasciata* | AM | BO, BR, CO, EC, FG, GY, PE, SR | LC | 386 |
| *Boana geographica* | AM | BO, BR, CO, EC, FG, GY, PE, SR, VE | LC | 432 |
| *Boana gracilis* | AM | BR, PE | NA | 3 |
| *Boana hobbsi* | AM | BR, CO, VE | LC | 10 |
| *Boana hutchinsi* | AM | CO | LC | 1 |
| *Boana icamiaba* | AM | BR | NA | 13 |
| *Boana lanciformis* | AM | BO, BR, CO, EC, PE, VE | LC | 300 |
| *Boana lemai* | AM | GY | LC | 7 |
| *Boana leucocheila* | AM | BR | LC | 34 |
| *Boana maculateralis* | AM | PE | NA | 1 |
| *Boana melanopleura* | AM | PE | LC | 1 |
| *Boana microderma* | AM | BR, CO, PE | LC | 18 |
| *Boana multifasciata* | AM, CE | BO, BR, CO, EC, FG, GY, SR, VE | LC | 318 |
| *Boana nigra* | AM | EC | NA | 5 |
| *Boana nympha* | AM | BR, EC, PE | LC | 6 |
| *Boana ornatissima* | AM | BR, FG, SR | LC | 7 |
| *Boana palaestes* | AM | PE | EN | 1 |
| *Boana picturata* | AM, PC | EC | LC | 1 |
| *Boana punctata* | AF, AM, CE, PN | BO, BR, CO, EC, FG, PE, SR | LC | 252 |
| *Boana raniceps* | AF, AM, CA, CE, PM, PN | BO, BR, SR | LC | 188 |
| *Boana roraima* | AM | GY | LC | 8 |
| *Boana semilineata* | AF, AM | BR | LC | 1 |
| *Boana sibleszi* | AM | GY, VE | LC | 13 |
| *Boana wavrini* | AM | BR, SR, VE | LC | 54 |
| *Boana xerophylla* | AM, NA*, NR* | BR, CO, SR | NA | 3 |
| *Corythomantis greeningi* | AF, AM, CA, CE | BR | LC | 1 |
| *Dendropsophus acreanus* | AM | BO, BR, PE | LC | 35 |
| *Dendropsophus aperomeus* | AM | PE | LC | 3 |
| *Dendropsophus arndti* | AM | BO | NA | 2 |
| *Dendropsophus bifurcus* | AM | CO, EC, PE | LC | 74 |
| *Dendropsophus bilobatus* | AM | BR | NA | 2 |
| *Dendropsophus bokermanni* | AM | BR, CO, EC, PE | LC | 46 |
| *Dendropsophus branneri* | AF, AM | BR | LC | 8 |
| *Dendropsophus brevifrons* | AM | BR, CO, EC, FG, GY, PE, SR | LC | 114 |
| *Dendropsophus cachimbo* | AM | BR | DD | 1 |
| *Dendropsophus counani* | AM | BR | NA | 2 |
| *Dendropsophus cruzi* | AM, CE | BR | LC | 16 |
| *Dendropsophus elianeae* | AF, AM, CE, PN | BR | LC | 1 |
| *Dendropsophus frosti* | AM | BR | LC | 2 |
| *Dendropsophus gaucheri* | AM | BR, SR | LC | 3 |
| *Dendropsophus haraldschultzi* | AM | BR, CO, PE | LC | 26 |
| *Dendropsophus joannae* | AM | BR, PE | DD | 8 |
| *Dendropsophus juliani* | AM | BR | LC | 2 |
| *Dendropsophus kamagarini* | AM | BO, BR, PE | NA | 13 |
| *Dendropsophus koechlini* | AM | BR, PE | LC | 22 |
| *Dendropsophus kubricki* | AM | PE | NA | 3 |
| *Dendropsophus leali* | AM | BO, BR, CO, EC, PE | LC | 47 |
| *Dendropsophus leucophyllatus* | AM | BO, BR, CO, EC, FG, GY, PE, SR | LC | 286 |
| *Dendropsophus manonegra* | AM | CO | LC | 6 |
| *Dendropsophus mapinguari* | AM | BR | NA | 1 |
| *Dendropsophus marmoratus* | AM | BR, CO, EC, GY, PE, SR, VE | LC | 145 |
| *Dendropsophus mathiassoni* | AM | CO | LC | 9 |
| *Dendropsophus melanargyreus* | AM, CE, PN | BO, BR | LC | 39 |
| *Dendropsophus microcephalus* | AM, CA, NR* | BR, CO, EC, GY, PE, VE | LC | 73 |
| *Dendropsophus minimus* | AM | BO, BR, CO, EC, GY, PE, SR, VE | DDLC | 228 |
| *Dendropsophus minusculus* | AF, AM, CE, OR | BR, FG, GY | LC | 30 |
| *Dendropsophus minutus* | AF, AM, CA, CE, PM, PN | BO, EC | LC | 3 |
| *Dendropsophus miyatai* | AM | BR, CO, EC, PE | LC | 11 |
| *Dendropsophus nanus* | AF, AM, CA, CE, PM, PN | BO, BR, FG | LC | 113 |
| *Dendropsophus ozzyi* | AM | BR | NA | 2 |
| *Dendropsophus parviceps* | AM | BR, CO, EC, PE, VE | LC | 165 |
| *Dendropsophus pauiniensis* | AM | BR | LC | 1 |
| *Dendropsophus reticulatus* | AM | BR, CO, EC, PE | NA | 9 |
| *Dendropsophus rhodopeplus* | AM | BR, CO, EC, PE | LC | 110 |
| *Dendropsophus riveroi* | AM | BO, BR, CO, EC, PE | LC | 40 |
| *Dendropsophus rossalleni* | AM | BR, CO, EC, PE | LC | 44 |
| *Dendropsophus rozenmani* | AM | BO | NA | 1 |
| *Dendropsophus rubicundulus* | AF, AM, CE, PN | BR | LC | 4 |
| *Dendropsophus sarayacuensis* | AM | BR, CO, EC, PE, VE | LC | 113 |
| *Dendropsophus schubarti* | AM | BO, BR, PE | LC | 20 |
| *Dendropsophus shiwiarum* | AM | CO | NA | 1 |
| *Dendropsophus soaresi* | AF, AM, CA, CE | BR | LC | 1 |
| *Dendropsophus timbeba* | AM | BR, PE | LC | 18 |
| *Dendropsophus tintinnabulum* | AM | BR, CO | DD | 3 |
| *Dendropsophus triangulum* | AM | BR, CO, EC, PE | LC | 167 |
| *Dendropsophus vraemi* | AM | PE | NA | 1 |
| *Dendropsophus walfordi* | AM | BR | LC | 37 |
| *Dendropsophus xapuriensis* | AM | BR | LC | 7 |
| *Dryaderces inframaculata* | AM | BR | DD | 20 |
| *Dryaderces pearsoni* | AM | BO, PE | LC | 5 |
| *Hyla imitator* | AM | BR | DD | 1 |
| *Hyloscirtus alytolylax* | AM, AN | EC | NT | 1 |
| *Hyloscirtus armatus* | AM, AN | PE | NT | 11 |
| *Hyloscirtus hillisi* | AM | EC | NA | 3 |
| *Hyloscirtus lindae* | AM | CO, EC | LC | 4 |
| *Hyloscirtus pacha* | AM | EC | DD | 3 |
| *Hyloscirtus pantostictus* | AM | EC | CR | 6 |
| *Hyloscirtus phyllognathus* | AM, AN | CO, EC, PE | LC | 59 |
| *Hyloscirtus psarolaimus* | AM | EC | VU | 4 |
| *Hyloscirtus staufferorum* | AM | EC | EN | 2 |
| *Hyloscirtus torrenticola* | AM | CO, EC | VU | 7 |
| *Lysapsus bolivianus* | AM | BO, BR | DD | 14 |
| *Lysapsus laevis* | AM | BR, GY | LC | 25 |
| *Lysapsus limellum* | AM, CE, CH, PM, PN | BO, BR, GY | LC | 69 |
| *Myersiohyla chamaeleo* | AM | BR, VE | NA | 8 |
| *Myersiohyla liliae* | AM | GY | EN | 3 |
| *Myersiohyla loveridgei* | AM | VE | NT | 2 |
| *Myersiohyla neblinaria* | AM | VE | LC | 3 |
| *Nesorohyla kanaima* | AM | GY | EN | 16 |
| *Nyctimantis rugiceps* | AM | EC, PE | LC | 11 |
| *Osteocephalus alboguttatus* | AM | EC, PE | LC | 9 |
| *Osteocephalus buckleyi* | AM | BO, BR, CO, EC, GY, PE, VE | LC | 73 |
| *Osteocephalus cabrerai* | AM | BR, CO, EC, FG, GY, PE | LC | 23 |
| *Osteocephalus cannatellai* | AM | CO, EC | NA | 3 |
| *Osteocephalus castaneicola* | AM | BR | LC | 5 |
| *Osteocephalus deridens* | AM | BR, CO, EC, PE | LC | 21 |
| *Osteocephalus fuscifacies* | AM | EC, PE | LC | 4 |
| *Osteocephalus helenae* | AM | BR, GY | DD | 5 |
| *Osteocephalus heyeri* | AM | BR | LC | 2 |
| *Osteocephalus leoniae* | AM | PE | LC | 1 |
| *Osteocephalus leprieurii* | AM | BO, BR, CO, EC, FG, GY, PE, VE | LC | 383 |
| *Osteocephalus melanops* | AM | BR | NA | 2 |
| *Osteocephalus mimeticus* | AM | BR, PE | NA | 12 |
| *Osteocephalus mutabor* | AM | EC, PE | LC | 10 |
| *Osteocephalus omega* | AM | CO | NA | 1 |
| *Osteocephalus oophagus* | AM | BR, FG, GY, SR | LC | 80 |
| *Osteocephalus planiceps* | AM | BR, CO, EC, PE, VE | LC | 68 |
| *Osteocephalus sangay* | AM | EC | NA | 4 |
| *Osteocephalus subtilis* | AM | BR | LC | 3 |
| *Osteocephalus taurinus* | AM, CE, OR | BO, BR, CO, EC, FG, GY, PE, SR, VE | LC | 806 |
| *Osteocephalus verruciger* | AM | CO, EC, PE | LC | 55 |
| *Osteocephalus vilarsi* | AM | BR | NA | 3 |
| *Osteocephalus yasuni* | AM | BR, CO, EC, PE | LC | 15 |
| *Pseudis laevis* | AM | BR | LC | 1 |
| *Pseudis paradoxa* | AF, AM, NA*, NR* | BO, BR, CO, GY, PE, SR | LC | 28 |
| *Pseudis tocantins* | AM, CE | BR | LC | 2 |
| *Scarthyla goinorum* | AM | BR, CO, PE | LC | 57 |
| *Scinax acuminatus* | AM, CE, PN | BO, CO, GY | LC | 3 |
| *Scinax baumgardneri* | AM | BR, VE | DD | 3 |
| *Scinax blairi* | AM | CO | LC | 3 |
| *Scinax boesemani* | AM | BR, FG, GY, SR, VE | LC | 115 |
| *Scinax boulengeri* | AM, NR* | BR, CO, GY | LC | 5 |
| *Scinax chiquitanus* | AM | PE | LC | 6 |
| *Scinax constrictus* | AM, CE | BR | LC | 4 |
| *Scinax cruentomma* | AM | BR, CO, EC, FG, PE | NA | 59 |
| *Scinax exiguus* | AM | VE | LC | 1 |
| *Scinax funereus* | AM | BR, CO, EC, PE, SR | LC | 42 |
| *Scinax fuscomarginatus* | AF, AM, CA, CE, PN | BO, BR, PE, SR, VE | LC | 68 |
| *Scinax fuscovarius* | AF, AM, CE, PN | BO, BR | LC | 15 |
| *Scinax garbei* | AM | BO, BR, CO, EC, PE | LC | 272 |
| *Scinax ictericus* | AM | BR, CO, PE | LC | 21 |
| *Scinax iquitorum* | AM | BR, PE | LC | 2 |
| *Scinax madeirae* | AM | CO | NA | 1 |
| *Scinax nasicus* | AF, AM, CE, PM, PN | BO, BR | LC | 5 |
| *Scinax nebulosus* | AM | BR, GY, SR | LC | 146 |
| *Scinax onca* | AM | BR | NA | 4 |
| *Scinax oreites* | AM | PE | LC | 14 |
| *Scinax pedromedinae* | AM | BO, BR, PE | LC | 21 |
| *Scinax proboscideus* | AM | BR, GY | LC | 4 |
| *Scinax rostratus* | AM, NR* | BR, CO, PE, VE | LC | 39 |
| *Scinax ruber* | AF, AM, CA, CE, NA*, NR* | BO, BR, CO, EC, FG, GY, PE, SR, VE | LC | 653 |
| *Scinax ruberoculatus* | AM | BR, FG | NA | 8 |
| *Scinax sateremawe* | AM | BR | NA | 1 |
| *Scinax strussmannae* | AM | BR | NA | 1 |
| *Scinax wandae* | AM, AN, OR | CO | LC | 6 |
| *Scinax x-signatus* | AM, AN, CA, CE, CB, NA* | BR, GY, SR | LC | 41 |
| *Sphaenorhynchus carneus* | AM | BR, CO, EC, PE | LC | 51 |
| *Sphaenorhynchus dorisae* | AM | BR, CO, EC, PE | LC | 60 |
| *Sphaenorhynchus lacteus* | AM | BO, BR, CO, EC, GY, PE, SR | LC | 157 |
| *Tepuihyla edelcae* | AM | VE | LC | 7 |
| *Tepuihyla exophthalma* | AM | GY | LC | 3 |
| *Tepuihyla rodriguezi* | AM | GY, VE | NT | 6 |
| *Tepuihyla shushupe* | AM | BR | LC | 1 |
| *Tepuihyla tuberculosa* | AM | CO, EC, PE | LC | 4 |
| *Tepuihyla warreni* | AM | GY | EN | 5 |
| *Trachycephalus coriaceus* | AM | BR, EC, GY, PE, SR | LC | 47 |
| *Trachycephalus cunauaru* | AM | BR, PE | NA | 13 |
| *Trachycephalus hadroceps* | AM | BR, SR | LC | 3 |
| *Trachycephalus macrotis* | AM | EC | NA | 1 |
| *Trachycephalus resinifictrix* | AM | BR, CO, EC, PE, SR, VE | LC | 88 |
| *Trachycephalus typhonius* | AF, AM, CA, CE, NA*, NR*, PM, PN | BO, BR, CO, EC, FG, GY, PE, SR, VE | LC | 463 |
| *Trachycephalus venezolanus* | AM | BR, VE | NA | 3 |
| **Leptodactylidae** |  |  |  |  |
| *Adenomera andreae* | AM | BO, BR, CO, EC, FG, GY, PE, SR, VE | LC | 578 |
| *Adenomera chicomendesi* | AM | BR, PE | NA | 3 |
| *Adenomera glauciae* | AM | BR | NA | 5 |
| *Adenomera guarayo* | AM | PE | NA | 2 |
| *Adenomera heyeri* | AM | BR, FG, SR | LC | 7 |
| *Adenomera hylaedactyla* | AM, CE | BO, BR, CO, EC, FG, GY, PE, SR, VE | LC | 361 |
| *Adenomera lutzi* | AM | GY | EN | 2 |
| *Adenomera martinezi* | AM, CE | BR | LC | 5 |
| *Adenomera nana* | AF, AM | BR | LC | 1 |
| *Adenomera phonotriccus* | AM | BR | NA | 3 |
| *Edalorhina nasuta* | AM | PE | DD | 1 |
| *Edalorhina perezi* | AM | BR, CO, EC, PE | LC | 127 |
| *Engystomops freibergi* | AM | BR, PE | LC | 146 |
| *Engystomops petersi* | AM | BO, BR, CO, EC, FG, PE | LC | 128 |
| *Engystomops pustulosus* | AM, NR* | VE | LC | 1 |
| *Hydrolaetare dantasi* | AM | BR | LC | 3 |
| *Hydrolaetare schmidti* | AM | BO, BR, CO, PE | LC | 13 |
| *Leptodactylus bolivianus* | AM, CE, NR* | BO, BR, CO, GY, PE, SR, VE | LC | 94 |
| *Leptodactylus bufonius* | AM, CH, PN | BO | LC | 3 |
| *Leptodactylus colombiensis* | AM, AN, OR | CO | LC | 3 |
| *Leptodactylus didymus* | AM | BR, PE | LC | 24 |
| *Leptodactylus diedrus* | AM | BR, PE, VE | LC | 3 |
| *Leptodactylus discodactylus* | AM | BR, CO, EC, PE | LC | 53 |
| *Leptodactylus elenae* | AM, CE, CH, PN | BO, BR | LC | 5 |
| *Leptodactylus fragilis* | AM, NR* | CO | LC | 2 |
| *Leptodactylus fuscus* | AD* | BO, BR, CO, EC, GY, SR, VE | LC | 212 |
| *Leptodactylus griseigularis* | AM | BR, PE | LC | 20 |
| *Leptodactylus guianensis* | AM | BR, GY, SR, VE | LC | 26 |
| *Leptodactylus insularum* | AM, NR* | CO | LC | 2 |
| *Leptodactylus intermedius* | AM | CO | NA | 2 |
| *Leptodactylus knudseni* | AM | BR, CO, EC, FG, GY, PE, SR, VE | LC | 220 |
| *Leptodactylus labyrinthicus* | AF, AM, CA, CE, CH, PN | BR, PE | LC | 56 |
| *Leptodactylus latinasus* | AF, AM, CE, CH, PN | BO | LC | 1 |
| *Leptodactylus latrans* | AF, AM, CA, CE, PM | BR | LC | 25 |
| *Leptodactylus leptodactyloides* | AM | BO, BR, CO, EC, FG, GY, PE, SR, VE | LC | 149 |
| *Leptodactylus lithonaetes* | AM | CO, VE | LC | 2 |
| *Leptodactylus longirostris* | AM | BR, FG, GY, SR, VE | LC | 65 |
| *Leptodactylus lutzi* | AM | GY | EN | 18 |
| *Leptodactylus macrosternum* | AF, AM, CA, CE, CH, PM, PN | BO, BR, GY, SR | LC | 174 |
| *Leptodactylus myersi* | AM | BR, GY, SR | LC | 18 |
| *Leptodactylus mystaceus* | AF, AM, CA, CE | BO, BR, CO, EC, FG, GY, PE, SR, VE | LC | 428 |
| *Leptodactylus paraensis* | AM | BR | LC | 124 |
| *Leptodactylus pascoensis* | AM | PE | DD | 2 |
| *Leptodactylus pentadactylus* | AM | BO, BR, CO, EC, FG, PE, SR | LC | 446 |
| *Leptodactylus petersii* | AM | BO, BR, CO, EC, FG, GY, PE, SR, VE | LC | 458 |
| *Leptodactylus podicipinus* | AM, CE, CH, PN | BO, BR, CO, EC, PE, SR | LC | 153 |
| *Leptodactylus pustulatus* | AM, CA, CE | BR | LC | 16 |
| *Leptodactylus rhodomystax* | AM | BR, CO, EC, FG, GY, PE, SR | LC | 332 |
| *Leptodactylus rhodonotus* | AM | BR, CO, PE | LC | 60 |
| *Leptodactylus riveroi* | AM | BR, CO, SR, VE | LC | 53 |
| *Leptodactylus rugosus* | AM | GY, VE | LC | 16 |
| *Leptodactylus stenodema* | AM | BR, CO, EC, FG, PE, SR | LC | 37 |
| *Leptodactylus troglodytes* | AF, AM, CA, CE | BR | LC | 6 |
| *Leptodactylus validus* | AM, NA* | BR, CO, FG, GY, SR, VE | LC | 36 |
| *Leptodactylus vastus* | AF, AM, CA, CE | BR | LC | 1 |
| *Leptodactylus wagneri* | AM, CE, PC | BO, BR, CO, EC, PE, SR, VE | LC | 164 |
| *Lithodytes lineatus* | AM, AN, OR | BO, BR, CO, EC, GY, PE, SR | LC | 313 |
| *Physalaemus albonotatus* | AM, CE, CH, PN | BR | LC | 4 |
| *Physalaemus biligonigerus* | AF, AM, CE, CH, PM, PN | BO, BR, EC | LC | 4 |
| *Physalaemus centralis* | AM, CE | BR | LC | 26 |
| *Physalaemus cuvieri* | AF, AM, CA, CE, PM, PN | BO, BR, CO, GY | LC | 84 |
| *Physalaemus ephippifer* | AM | BR, SR, VE | LC | 180 |
| *Physalaemus fischeri* | AM | CO, VE | LC | 4 |
| *Physalaemus kroyeri* | AF, AM, CA | BR | LC | 3 |
| *Physalaemus nattereri* | AM, CE, PN | BR | LC | 7 |
| *Physalaemus petersi* | AM | FG, PE | LC | 4 |
| *Pleurodema brachyops* | AM, NR* | BR, GY, VE | LC | 18 |
| *Pleurodema fuscomaculatum* | AM | BO, BR | DD | 4 |
| *Pleurodema marmoratum* | AM, AN | BO, PE | VU | 12 |
| *Pseudopaludicola boliviana* | AM, CE, CH | BO, BR, CO, GY, VE | LC | 27 |
| *Pseudopaludicola canga* | AM | BR | DD | 8 |
| *Pseudopaludicola ceratophyes* | AM | PE | LC | 1 |
| *Pseudopaludicola hyleaustralis* | AM | BR | NA | 2 |
| *Pseudopaludicola jazmynmcdonaldae* | AM | BR | NA | 2 |
| *Pseudopaludicola mystacalis* | AM, CA, CE, PN | BR | LC | 2 |
| *Pseudopaludicola pusilla* | AM, NR* | BO, GY, SR, VE | LC | 4 |
| *Pseudopaludicola saltica* | AM, CE, PN | BR | LC | 3 |
| *Pseudopaludicola ternetzi* | AM, CE | BR | LC | 1 |
| **Microhylidae** |  |  |  |  |
| *Adelastes hylonomos* | AM | BR, GY, VE | DD | 5 |
| *Chiasmocleis albopunctata* | AF, AM, CE, PN | BO, BR | LC | 3 |
| *Chiasmocleis anatipes* | AM | BR, EC, PE | LC | 5 |
| *Chiasmocleis antenori* | AM | BR, CO, EC, PE | LC | 15 |
| *Chiasmocleis avilapiresae* | AM | BR | LC | 53 |
| *Chiasmocleis bassleri* | AM | BR, CO, EC, PE | LC | 95 |
| *Chiasmocleis carvalhoi* | AM | BR, CO, PE | LC | 24 |
| *Chiasmocleis haddadi* | AM | SR | NA | 1 |
| *Chiasmocleis hudsoni* | AM | BR, FG, VE | LC | 47 |
| *Chiasmocleis jimi* | AM | BR, GY | DD | 13 |
| *Chiasmocleis magnova* | AM | PE | LC | 6 |
| *Chiasmocleis parkeri* | AM | EC | NA | 7 |
| *Chiasmocleis royi* | AM | BR, PE | NA | 8 |
| *Chiasmocleis shudikarensis* | AM | BR, GY, SR | LC | 21 |
| *Chiasmocleis supercilialba* | AM | BR, PE | NA | 4 |
| *Chiasmocleis tridactyla* | AM | BR, PE | LC | 4 |
| *Chiasmocleis ventrimaculata* | AM | BR, CO, EC, PE | LC | 45 |
| *Ctenophryne geayi* | AM | BR, CO, EC, FG, GY, PE | LC | 124 |
| *Dermatonotus muelleri* | AF, AM, CE, CH, PN | BO, BR | LC | 4 |
| *Elachistocleis bicolor* | AF, AM, CE, CH, PM, PN | BO, BR, CO, PE | LC | 34 |
| *Elachistocleis bumbameuboi* | AM | BR | DD | 2 |
| *Elachistocleis carvalhoi* | AM | BR | LC | 10 |
| *Elachistocleis cesarii* | AF, AM, CE | BR | NA | 1 |
| *Elachistocleis helianneae* | AM | BR | LC | 8 |
| *Elachistocleis magna* | AM | BR | NA | 3 |
| *Elachistocleis matogrosso* | AM, CE, PN | BR | LC | 1 |
| *Elachistocleis muiraquitan* | AM | BR | NA | 10 |
| *Elachistocleis surinamensis* | AM, NA* | GY | LC | 4 |
| *Elachistocleis surumu* | AM | BR | DD | 5 |
| *Hamptophryne alios* | AM | PE | DD | 3 |
| *Hamptophryne boliviana* | AM | BO, BR, CO, EC, GY, PE, SR, VE | LC | 129 |
| *Otophryne pyburni* | AM | BR, CO, FG, GY, SR, VE | LC | 14 |
| *Otophryne robusta* | AM | CO, GY, VE | LC | 14 |
| *Otophryne steyermarki* | AM | GY | LC | 4 |
| *Synapturanus ajuricaba* | AM | BR | NA | 4 |
| *Synapturanus mesomorphus* | AM | GY | NA | 7 |
| *Synapturanus mirandaribeiroi* | AM | BR, CO, FG | LC | 15 |
| *Synapturanus rabus* | AM | BR, CO, EC, PE | LC | 11 |
| *Synapturanus salseri* | AM | BR, CO, GY, VE | LC | 17 |
| *Synapturanus zombie* | AM | BR, FG | NA | 4 |
| *Syncope antenori* | AM | CO, EC, PE | LC | 9 |
| *Syncope carvalhoi* | AM | CO, EC, PE | LC | 8 |
| **Odontophrynidae** |  |  |  |  |
| *Proceratophrys concavitympanum* | AM | BR | DD | 36 |
| *Proceratophrys cristiceps* | AF, AM, CA | BR | LC | 2 |
| *Proceratophrys korekore* | AM | BR | NA | 4 |
| *Proceratophrys rondonae* | AM | BR | NA | 1 |
| **Phyllomedusidae** |  |  |  |  |
| *Agalychnis buckleyi* | AM | BO, BR, CO, EC, PE, SR, VE | LC | 59 |
| *Agalychnis hulli* | AM | EC, PE | LC | 6 |
| *Callimedusa atelopoides* | AM | BR, PE | LC | 18 |
| *Callimedusa baltea* | AM | PE | EN | 1 |
| *Callimedusa duellmani* | AM | PE | DD | 6 |
| *Callimedusa ecuatoriana* | AM | EC | VU | 1 |
| *Callimedusa perinesos* | AM | CO, EC | EN | 19 |
| *Callimedusa tomopterna* | AM | BR, CO, EC, FG, PE, SR, VE | LC | 185 |
| *Cruziohyla craspedopus* | AM | BR, CO, EC, PE | LC | 19 |
| *Phyllomedusa bicolor* | AM | BR, CO, FG, GY, PE, SR, VE | LC | 171 |
| *Phyllomedusa boliviana* | AM, AN, CE | BO, BR, CO, PE | LC | 6 |
| *Phyllomedusa camba* | AM | BO, BR, PE | LC | 74 |
| *Phyllomedusa chaparroi* | AM | PE | NA | 8 |
| *Phyllomedusa coelestis* | AM | CO | LC | 1 |
| *Phyllomedusa ecuatoriana* | AM | EC | VU | 1 |
| *Phyllomedusa sauvagii* | AM, CE, CH, PN | BO | LC | 2 |
| *Phyllomedusa tarsius* | AM | BR, CO, EC, GY, PE | LC | 78 |
| *Phyllomedusa vaillantii* | AM | BO, BR, CO, EC, FG, GY, PE, SR, VE | LC | 351 |
| *Pithecopus azureus* | AM, CE, CH, PN | BO, BR | DD | 2 |
| *Pithecopus hypochondrialis* | AM, CA, CE | BO, BR, CO, GY, SR | LC | 194 |
| *Pithecopus palliatus* | AM | BO, BR, EC, PE | LC | 54 |
| **Pipidae** |  |  |  |  |
| *Pipa arrabali* | AM | BR, GY | LC | 40 |
| *Pipa aspera* | AM | FG, SR | LC | 3 |
| *Pipa carvalhoi* | AF, AM, CA | EC | LC | 1 |
| *Pipa pipa* | AM | BR, CO, EC, GY, PE, SR, VE | LC | 128 |
| *Pipa snethlageae* | AM | BR, CO, PE | LC | 10 |
| **Ranidae** |  |  |  |  |
| *Lithobates palmipes* | AF, AM, CE, NR* | BO, BR, CO, EC, GY, PE, VE | LC | 150 |
| *Lithobates vaillanti* | AM, NR* | CO | LC | 1 |
| ***Strabomantidae*** |  |  |  |  |
| *Phrynopus bracki* | AM | PE | DD | 2 |
| *Phrynopus daemon* | AM | PE | EN | 2 |
| *Phrynopus dagmarae* | AM | PE | EN | 3 |
| *Phrynopus juninensis* | AM, AN | PE | CR | 2 |
| *Phrynopus kauneorum* | AM | PE | EN | 1 |
| *Phrynopus mariellaleo* | AM | PE | NA | 1 |
| *Phrynopus montium* | AM | PE | EN | 1 |
| *Phrynopus peraccai* | AM | EC | DD | 2 |
| *Phrynopus tribulosus* | AM | PE | LC | 2 |
| *Phrynopus vestigiatus* | AM | PE | EN | 1 |
| **Telmatobiidae** |  |  |  |  |
| *Telmatobius atahualpai* | AM | PE | VU | 1 |
| *Telmatobius brevirostris* | AM | PE | EN | 1 |
| *Telmatobius jelskii* | AM, AN | PE | NT | 3 |
| *Telmatobius macrostomus* | AM, AN | PE | EN | 1 |
| *Telmatobius mendelsoni* | AM | PE | CR | 2 |
| *Telmatobius niger* | AM, AN | EC, PE | CR | 3 |
| *Telmatobius rimac* | AM, AN | PE | VU | 1 |
| *Telmatobius simonsi* | AM | BO | CR | 1 |
| *Telmatobius timens* | AM | PE | CR | 9 |
| *Telmatobius truebae* | AM | PE | VU | 6 |
| **Caudata** |  |  |  |  |
| **Plethodontidae** |  |  |  |  |
| *Bolitoglossa adspersa* | AM | CO | NT | 1 |
| *Bolitoglossa altamazonica* | AM | BO, BR, CO, EC, PE | LC | 39 |
| *Bolitoglossa caldwellae* | AM | BR | NA | 2 |
| *Bolitoglossa digitigrada* | AM | PE | DD | 1 |
| *Bolitoglossa equatoriana* | AM | EC | LC | 10 |
| *Bolitoglossa palmata* | AM | CO, EC | LC | 8 |
| *Bolitoglossa paraensis* | AM | BR | DD | 7 |
| *Bolitoglossa peruviana* | AM | EC, PE | DD | 21 |
| *Bolitoglossa tapajonica* | AM | BR | NA | 3 |
| **Gymnophiona** |  |  |  |  |
| **Caeciliidae** |  |  |  |  |
| *Atretochoana eiselti* | AM | BR | DD | 1 |
| *Caecilia abitaguae* | AM | EC | DD | 2 |
| *Caecilia attenuata* | AM | EC | DD | 1 |
| *Caecilia bokermanni* | AM | EC | LC | 1 |
| *Caecilia disossea* | AM | BR, EC, PE | LC | 9 |
| *Caecilia dunni* | AM | EC | DD | 2 |
| *Caecilia gracilis* | AM | BR, FG | LC | 5 |
| *Caecilia inca* | AM | EC, PE | DD | 2 |
| *Caecilia marcusi* | AM | BR | LC | 1 |
| *Caecilia museugoeldi* | AM | FG | NA | 2 |
| *Caecilia orientalis* | AM | EC | LC | 14 |
| *Caecilia subdermalis* | AM | CO | LC | 1 |
| *Caecilia subnigricans* | AM | EC | LC | 1 |
| *Caecilia tentaculata* | AF, AM, NR* | BR, CO, EC, FG, GY, PE, SR | DD | 28 |
| *Nectocaecilia petersii* | AM | BR | LC | 1 |
| *Oscaecilia bassleri* | AM | CO, EC, PE | LC | 19 |
| **Rhinatrematidae** |  |  |  |  |
| *Amazops amazops* | AM | EC | NA | 1 |
| *Epicrionops bicolor* | AM, PC | PE | LC | 3 |
| *Epicrionops petersi* | AM | EC | LC | 5 |
| *Rhinatrema bivittatum* | AM | BR, FG | LC | 6 |
| *Rhinatrema gilbertogili* | AM | BR | NA | 2 |
| *Rhinatrema nigrum* | AM | GY | LC | 4 |
| *Rhinatrema ron* | AM | BR | NA | 3 |
| *Rhinatrema shiv* | AM | GY | EN | 2 |
| *Rhinatrema uaiuai* | AM | BR | NA | 1 |
| **Siphonopidae** |  |  |  |  |
| *Brasilotyphlus dubium* | AM | BR | NA | 3 |
| *Brasilotyphlus guarantanus* | AM | BR | NA | 3 |
| *Microcaecilia albiceps* | AM | CO, EC, PE | LC | 9 |
| *Microcaecilia marvaleewakeae* | AM | BR | NA | 3 |
| *Microcaecilia rabei* | AM | GY, SR | DD | 2 |
| *Microcaecilia taylori* | AM | BR, FG, SR | LC | 6 |
| *Microcaecilia unicolor* | AM | BR, FG | LC | 8 |
| *Siphonops annulatus* | AF, AM | BO, BR, CO, EC, PE | LC | 33 |
| **Typhlonectidae** |  |  |  |  |
| *Chthonerpeton onorei* | AM | EC | DD | 1 |
| *Potomotyphlus kaupii* | AM | BR, CO, EC, PE, VE | LC | 17 |
| *Typhlonectes compressicauda* | AM | BR, CO, FG, GY, PE | LC | 32 |
| *Typhlonectes natans* | AM, AN, CB | FG | LC | 1 |
