# Supplementary material for: Amazonian amphibians: diversity, spatial distribution patterns, conservation and sampling deficits
Source: Biodivers Data J. 2024 Oct 1;12:e109785. doi: 10.3897/BDJ.12.e109785 (PMC11471977; doi:10.3897/BDJ.12.e109785)

**Supplementary Material 7**

**Amazon amphibians: diversity, distribution patterns, conservation and sampling deficits**

Marcos Penhacek, Thadeu Sobral de Souza, Jessie Pereira dos Santos, Vinicius Guerra & Domingos de Jesus Rodrigues

**Figure S1.** Number of amphibians from the Amazon domain. Sample sites (A) and records (B).


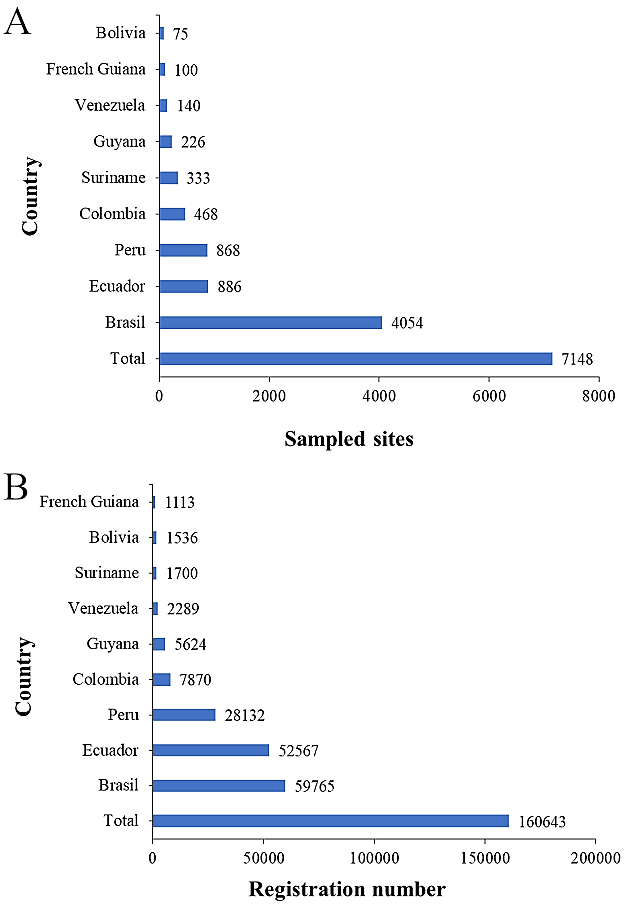

Supplement: Supplementary material 7 — Number of amphibians from the Amazon domain [file bdj-12-e109785-s007.docx]
