# Supplementary material for: Amazonian amphibians: diversity, spatial distribution patterns, conservation and sampling deficits
Source: Biodivers Data J. 2024 Oct 1;12:e109785. doi: 10.3897/BDJ.12.e109785 (PMC11471977; doi:10.3897/BDJ.12.e109785)
Supplement: Supplementary material 8 — Richness and endemism of amphibian species [file bdj-12-e109785-s008.docx]

**Supplementary Material 8**

**Amazon amphibians: diversity, distribution patterns, conservation and sampling deficits**

Marcos Penhacek, Thadeu Sobral de Souza, Jessie Pereira dos Santos, Vinicius Guerra & Domingos de Jesus Rodrigues

**Table S1.** Richness and endemism of amphibian species, area size (Km²), main rivers and country(ies) of each of the 52 drainage sub-basins considered in this study for the Amazon domain.

| **Basins** | **Tributaries** | **Richness** | **Endemic** | **Área (km^2^)** | **Coutry** |
| --- | --- | --- | --- | --- | --- |
| Basins_01 | Gurupi | 65 | 2 | 93.892 | Brazil |
| Basins_02 | Capim Guama | 76 | 1 | 80.583 | Brazil |
| Basins_03 | Belem | 44 | 1 | 107.423 | Brazil |
| Basins_04 | Tocantis | 100 | 7 | 166.01 | Brazil |
| Basins_05 | Vila Nova | 74 | 0 | 30.536 | Brazil |
| Basins_06 | Araguari | 96 | 3 | 42.205 | Brazil |
| Basins_07 | Oyapok | 81 | 5 | 91.27 | Brazil French Guiana |
| Basins_08 | Maroni | 55 | 5 | 66.116 | French Guiana Suriname |
| Basins_09 | Suriname | 65 | 3 | 65.339 | French Guiana Suriname |
| Basins_10 | Jaru | 41 | 0 | 59.206 | Brazil |
| Basins_11 | Xingu | 118 | 5 | 470.153 | Brazil |
| Basins_12 | Pari | 39 | 0 | 39.645 | Brazil |
| Basins_13 | Curua | 54 | 0 | 28.004 | Brazil |
| Basins_14 | Curua Uma | 34 | 0 | 31.116 | Brazil |
| Basins_15 | Tapajos | 141 | 11 | 373.973 | Brazil |
| Basins_16 | Trombetas | 73 | 0 | 155.316 | Brazil |
| Basins_17 | Courentyne | 79 | 4 | 64.002 | Suriname |
| Basins_18 | Berbice | 53 | 0 | 30.444 | Guyana |
| Basins_19 | Essequibo | 127 | 28 | 149.054 | Guyana |
| Basins_20 | Amacuru Aruta | 33 | 0 | 36.342 | Guyana Venezuela |
| Basins_21 | Maués Açu | 62 | 4 | 132784 | Brazil |
| Basins_22 | Amazonas | 138 | 3 | 231.023 | Brazil |
| Basins_23 | Uatumã | 78 | 1 | 67.409 | Brazil |
| Basins_24 | Negro (baixo) | 86 | 2 | 51.738 | Brazil |
| Basins_25 | Unini | 1 | 0 | 28.35 | Brazil |
| Basins_26 | Jauoperi | 8 | 0 | 39.646 | Brazil |
| Basins_27 | Rio Branco | 82 | 2 | 189.946 | Brazil |
| Basins_28 | Negro Demini | 21 | 1 | 35.659 | Brazil |
| Basins_29 | Aracã | 1 | 0 | 39.592 | Brazil |
| Basins_30 | Negro (alto) | 60 | 0 | 135.694 | Brazil |
| Basins_31 | Negro (médio) | 46 | 2 | 126.851 | Brazil Colômbia Venezuela |
| Basins_32 | Uapés | 36 | 1 | 64.085 | Brazil Colômbia |
| Basins_33 | Caroni | 24 | 4 | 83879 | Venezuela |
| Basins_34 | Caura | 7 | 1 | 70.661 | Venezuela |
| Basins_35 | Orinoco Ventuani | 36 | 8 | 100.53 | Venezuela |
| Basins_36 | Orinoco (médio) | 38 | 2 | 46.061 | Colômbia Venezuela |
| Basins_37 | Guaviare | 84 | 7 | 150.811 | Colômbia Venezuela |
| Basins_38 | Madeira (baixo) | 55 | 0 | 55.085 | Brazil |
| Basins_39 | Roosevelt | 85 | 0 | 138.552 | Brazil |
| Basins_40 | Madeira (médio) | 81 | 0 | 77.693 | Brazil |
| Basins_41 | Jiparaná | 76 | 0 | 69.266 | Brazil |
| Basins_42 | Madeira (alto) | 125 | 4 | 93.08 | Brazil |
| Basins_43 | Itenez O Guaporé | 62 | 0 | 144.535 | Bolívia |
| Basins_44 | Mamoré | 20 | 1 | 46.681 | Bolívia |
| Basins_45 | Mamoré o Grande | 62 | 6 | 145.982 | Bolívia |
| Basins_46 | Beni | 218 | 31 | 253.264 | Bolívia Peru |
| Basins_47 | Purus | 165 | 6 | 377.156 | Brazil |
| Basins_48 | Jurua | 152 | 7 | 188.919 | Brazil |
| Basins_49 | Jupará Coquetá | 200 | 21 | 267.351 | Colômbia |
| Basins_50 | Patumaio | 345 | 82 | 509.177 | Ecuador Peru |
| Basins_51 | Ucayali | 191 | 33 | 241.692 | Brazil Peru |
| Basins_52 | Maranõn | 351 | 134 | 292.199 | Ecuador Peru |
