# Supplementary material for: Amazonian amphibians: diversity, spatial distribution patterns, conservation and sampling deficits
Source: Biodivers Data J. 2024 Oct 1;12:e109785. doi: 10.3897/BDJ.12.e109785 (PMC11471977; doi:10.3897/BDJ.12.e109785)

**Supplementary Material 10**

**Amazon amphibians: diversity, distribution patterns, conservation and sampling deficits**

Marcos Penhacek, Thadeu Sobral de Souza, Jessie Pereira dos Santos, Vinicius Guerra & Domingos de Jesus Rodrigues

**Figure S10.** Sum of occurrences for richness, distribution and abundance of amphibians in the Amazon. Sources: GBIF=1, VertNet=2, SpeciesLink=3, SiBBr=4, SISBIO=5, Scientific Articles=6, Gray Literature=7 and Fieldwork=8.


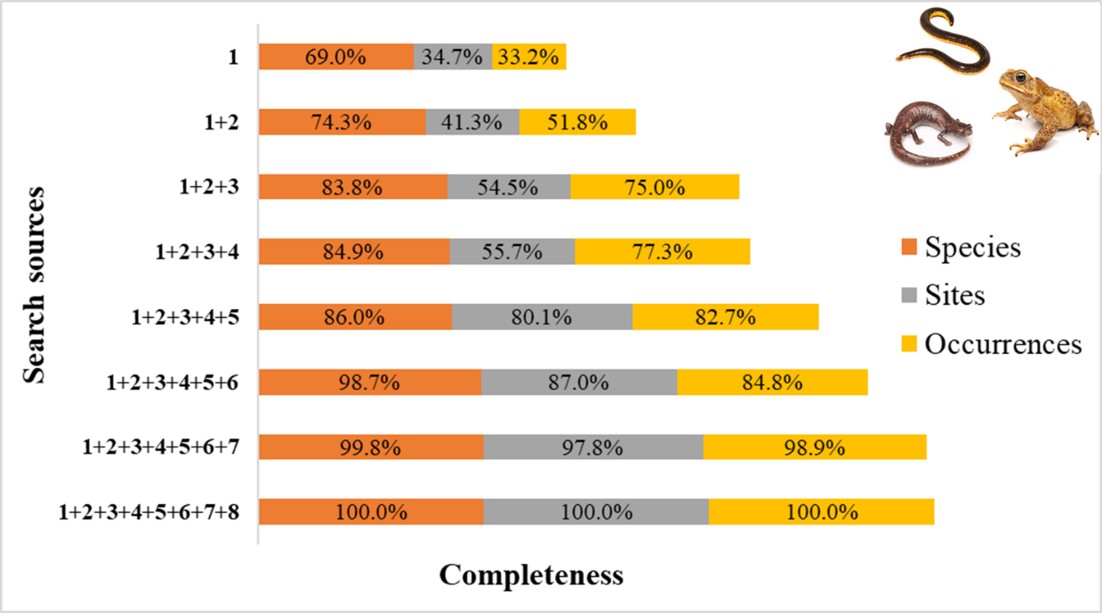

Supplement: Supplementary material 9 — Relationship between the total richness and number of endemic species [file bdj-12-e109785-s009.docx]
